# Synthesis of Bioactive Compounds from 3-Carene (II): Synthesis, Antifungal Activity and 3D-QSAR Study of (*Z*)- and (*E*)-3-Caren-5-One Oxime Sulfonates

**DOI:** 10.3390/molecules24030477

**Published:** 2019-01-29

**Authors:** Guo-Qiang Kang, Wen-Gui Duan, Gui-Shan Lin, You-Pei Yu, Xiao-Yu Wang, Sun-Zhong Lu

**Affiliations:** 1 School of Chemistry and Chemical Engineering, Guangxi University, Nanning 530004, Guangxi, China; m15877185573@163.com (G.-Q.K.); Yqj974548931@163.com (Y.-P.Y.); wxygxu@163.com (X.-Y.W.); 2 Guangxi Academy of Forestry, Nanning 530002, Guangxi, China; dwg105@gxu.edu.cn

**Keywords:** 3-carene, 3-caren-5-one oxime sulfonate, *Z*-*E* stereoisomers, antifungal activity, 3D-QSAR

## Abstract

A series of novel (*Z*)- and (*E*)-3-caren-5-one oxime sulfonates were designed and synthesized in search of potent antifungal agents. The structures of the intermediates and target compounds were confirmed by UV-Vis, FTIR, NMR, and ESI-MS. The in vitro antifungal activity of the target compounds was preliminarily evaluated against *Cercospora arachidicola*, *Physalospora piricola*, *Alternaria solani*, *Rhizoeotnia solani*, *Bipolaris maydis* and *Colleterichum orbicalare* at 50 µg/mL. The bioassay results indicated that the target compounds exhibited the best antifungal activity against *P. piricola*, in which compounds **4b**, **4f**, **4m**, **4e**, **4j**, **4l**, **4y**, **4d**, and **4p** had excellent inhibition rates of 100%, 100%, 100%, 92.9%, 92.9%, 92.9%, 92.9%, 85.7%, and 85.7%, respectively, showing much better antifungal activity than that of the commercial fungicide chlorothanil. Both the compounds **4y** and **4x** displayed outstanding antifungal activity of 100% against *B. myadis*, and the former also displayed outstanding antifungal activity of 100% against *R. solani*. In order to design more effective antifungal compounds against *P. piricola*, the analysis of three-dimensional quantitative structure-activity relationship (3D-QSAR) was carried out using the CoMFA method, and a reasonable and effective 3D-QSAR model (r^2^ = 0.990, q^2^ = 0.569) has been established.

## 1. Introduction

3-Carene, a naturally occurring bicyclic monoterpene, is a constituent of many turpentine oils and essential oils [1,2]. This biomass resource is particularly interesting as it has two active groups in the molecule, namely the carbon-carbon double bond and the gem-dimethylcyclopropane ring. It was reported that 3-carene showed a broad spectrum of activities, such as antimicrobial [3,4,5], antioxidant [4,5,6,7], anticancer [8,9], semiochemical [10,11], and fumigant properties [12,13]. 3-Carene-5-one can be prepared by selective allylic oxidation of 3-carene without breaking its natural skeleton, and this functional oxygen-containing derivative is a vital intermediate for manufacturing value-added chemicals. On the other hand, oxime sulfonates are widely applied to the fields of medicine and agrochemical because of their various biological properties, such as anti-proliferation [14,15], insecticidal [16,17,18], antifungal [19,20] and antibacterial activities [19].

Our research group has recently reported the synthesis of a series of structurally modified 3-carene derivatives and found that some of the title compounds exhibited excellent antifungal activity [21]. Herein, as continuation of our work on natural product-based bioactive compounds [22,23,24,25,26,27], a series of novel 3-caren-5-one oxime sulfonates were designed and synthesized by integrating bioactive oxime sulfonates into the skeleton of 3-carene. Structural characterization and antifungal evaluation of all the target compounds were carried out as well. Futhermore, through the 3D-QSAR analysis of the antifungal activity against *P. piricola*, the structure-activity quantitative relationship of the target compounds was found out. The effective 3D-QSAR model provided a theoretical basis for the subsequent optimization of the series of compounds and the discovery of new potential antifungal agents with high activity.

## 2. Results and Discussion

### 2.1. Synthesis and Characterization

**Scheme 1:**
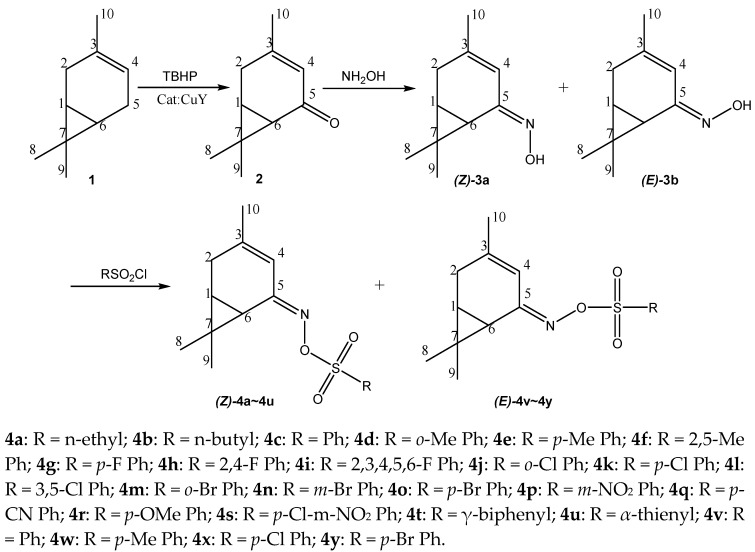
Synthesis of 3-caren-5-one oxime sulfonates (*Z*)-**4a**–**4u** and (*E*)-**4v**–**4y**.

As illustrated in the Scheme 1, 3-carene (**1**) was oxidized selectively using *tert*-butyl hydroperoxide (TBHP) as oxidant to obtain 3-caren-5-one (**2**).

3-Caren-5-one oxime (**3**, isomeric mixture) was prepared by oximation of compound **2** with NH_2_OH·HCl in a mixed solvent (EtOH:H_2_O = 5:1, *v*/*v*). Since the three-membered ring in the 3-carene molecule is very sensitive to ring-opening reactions under acidic conditions, sodium acetate was added to the reaction system to form a buffer system. Because the two oxime isomers possessed very similar R_f_ values and (*Z*)-**3a** was the dominant product (**3a**:**3b** = 7:1), the separation conditions were improved on the basis of a literature method [21]. It was found that (*E*)-**3b** can be separated easily from (*Z*)-**3a** by silica gel column chromatography after most of (*Z*)-**3a** was recrystallized from cyclohexane. The structures of the two stereo-isomers were confirmed by FTIR, ^1^H-NMR and ^13^C-NMR spectroscopy. In the ^1^H-NMR spectrum, the olefinic proton (C_4_-H) in the (*Z*)-**3a** showed a signal at 5.87 ppm, but the signal of another isomer (*E*)-**3b** was shifted to 6.64 ppm, which was in agreement with our previous work [21]. The ^13^C-NMR signals were assigned by HMBC and DEPT spectra.

Finally, 3-caren-5-one oxime sulfonates (*Z*)-**4a**–**4u** and (*E*)-**4v**–**4y** were synthesized by *O*- sulfonylation reaction of the corresponding oximes with sulfonyl chlorides. The crude products of (*Z*)-**4a**–**4u** were purified by silica gel chromatography, and (*E*)-**4v**–**4y** were purified by recrystallization from acetone-water. The target oxime sulfonate compounds were characterized by UV-Vis, FTIR, ^1^H-NMR, ^13^C-NMR, and ESI-MS. All the related spectra can be found in the Supplementary Materials. In the IR spectra, the moderate absorption bands at about 1652 cm^−1^ were attributed to the stretching vibrations of the C=N in the (*Z*)-isomers, while the corresponding signals of (*E*)-isomers were shifted to about 1641 cm^−1^. The strong absorption bands at about 1371 and 1195 cm^−1^ were assigned to the vibrations of O=S=O in the sulfonate moiety. In the ^1^H-NMR spectra, the protons of benzene ring showed signals at 6.99–8.84 ppm. The olefinic protons of the 3-carene scaffold in the (*Z*)-isomers showed signals at 5.75–5.94 ppm but the (*E*)-isomers showed signals at about 6.49 ppm. The ^13^C-NMR spectra of the target compounds showed peaks for the olefinic carbons of the 3-carene moiety at about 117 ppm (C_4_) and 149 ppm (C_3_) for the (*Z*)-isomers, however, the corresponding signals for the (*E*)-isomers showed peaks at about 112 ppm (C_4_) and 154 ppm (C_3_). In addition, the molecular weights of the intermediates and target compounds were confirmed by the ESI-MS.

### 2.2. Antifungal Activity

The antifungal activities of the target compounds (*Z*)-**4a**–**4u** and (*E*)-**4v**–**4y** were evaluated by *in vitro* method against speckle on peanut (*Cercospora arachidicola*), apple ring rot (*Physalospora piricola*), tomato early blight (*Alternaria solani*), rice sheath blight (*Rhizoeotnia solani*), corn southern leaf blight (*Bipolaris maydis*), and watermelon anthracnose (*Colleterichum orbicalare*) at 50 µg/mL. The results are listed in Table 1.

The bioassay results indicated that, at 50 µg/mL, all the target compounds presented obviously different antifungal activity against the six tested fungi. On the whole, most of the target compounds exhibited the best antifungal activity against *P. piricola*, in which compounds (*Z*)-**4b** (R = *n*-butyl), (*Z*)-**4f** (R = 2,5-Me Ph), (*Z*)-**4m** (R = *o*-Br Ph), (*Z*)-**4e** (R = *p*-Me Ph), (*Z*)-**4j (**R = *o*-Cl Ph), (*Z*)-**4l** (R = 3,5-Cl Ph), (*E*)-**4y** (R = *p*-Br Ph), (*Z*)-**4d** (R = *o*-Me Ph), and (*Z*)-**4p** (R = *m*-NO_2_ Ph) had significant inhibition rates of 100%, 100%, 100%, 92.9%, 92.9%, 92.9%, 92.9%, 85.7%, and 85.7%, respectively, showing much better antifungal activity than that of the commercial fungicide chlorothanil with the inhibition rate of 75.0%. It was found that both compounds (*E*)-**4y** (R = *p*-Br Ph) and (*E*)-**4x** (R = *p*-Cl Ph) displayed outstanding antifungal activity against *B. myadis*, with inhibition rate of 100%, and the former also displayed outstanding antifungal activity against *R. solani*, with inhibition rate of 100%, showing better antifungal activity than the positive control. Furthermore, the target compound (*Z*)-**4b** (R = n-butyl) exhibited better antifungal activity of 87.1% against *C. arachidicola* than that of the positive control. Therefore, compounds (*Z*)-**4b** (R = n-butyl), (*Z*)-**4f** (R = 2,5-Me Ph), (*Z*)-**4m** (R = *o*-Br Ph), (*E*)-**4y** (R = *p*-Br Ph), and (*E*)-**4x** (R = *p*-Cl Ph) are lead compounds worthy of further investigation.

Meanwhile, some compounds presented significant differences in antifungal activity between the (*Z*)- and (*E*)-isomers. For instance, compound (*E*)-**4y** (R = *p*-Br Ph) showed 7.8-, 6.5-, and 4.9-fold higher antifungal bioactivity against *B. myadis*, *P. piricola*, and *R. solani*, respectively, than its stereoisomer (*Z*)-**4o** (R = *p*-Br Ph). The obvious difference in antifungal activity between *E-Z* isomers is very meaningful to photoisomerization and drug resistance which require further studies.

### 2.3. CoMFA Analysis

**Figure 1:**
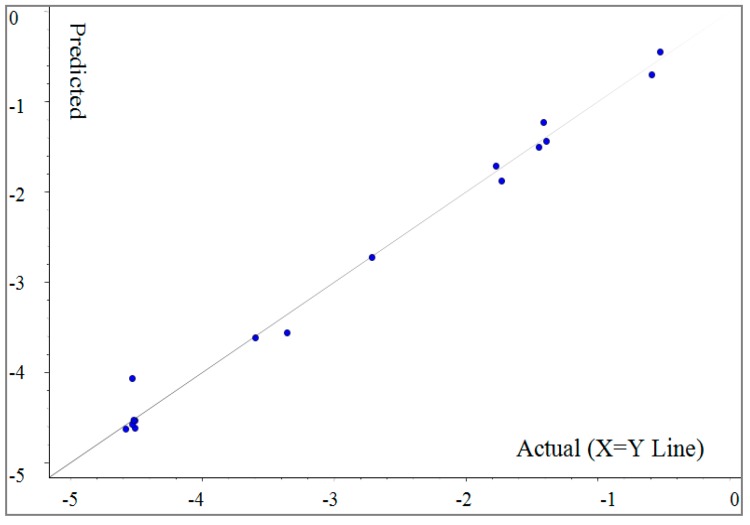
Predicted vs experimental ED values of CoMFA.

3D-QSAR analysis of the antifungal activity against *P. piricola* of the target compounds was carried out using the CoMFA method. The experimental and predicted activities values of training set were shown in Table 2. A predictive CoMFA model with the conventional correlation coefficient r^2^ = 0.990 and the cross-validated coefficient q^2^ = 0.569 was established (Table 3). As shown in Figure 1, all data were concentrated near the X = Y line, also indicating that the CoMFA model was reasonable and effective.

**Figure 2:**
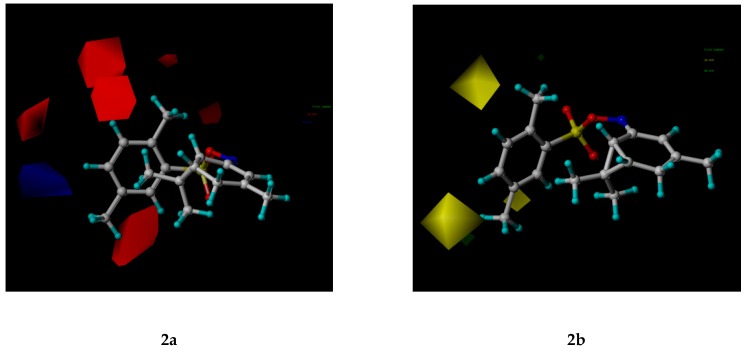
Contours of CoMFA analysis: (**2a**) Contours of electrostatic contribution were represented in red and blue; (**2b**) Contours of steric contribution were represented in yellow and green.

The electrostatic and steric contribution maps of CoMFA are shown in Figure 2. The contribution of the electrostatic field was 95% while the steric field was 5%, indicating that the electrostatic field of the compounds held a much greater influence on the antifungal activity against *P. piricola*. As shown in Figure 2a, the electrostatic contours were represented in distinguishable colors: red indicated that an increase in the negative charge will lead to an increase in the activity, while the blue contour defines the opposite. Therefore, the title compounds bearing an electron-withdrawing group at 3-position of benzene ring, such as (*Z*)-**4l** (R = 3,5-Cl Ph) and (*Z*)-**4p** (R = *m*-NO_2_ Ph), displayed higher antifungal activity against *P. piricola.*

In contrast, the introduction of electron-donating groups at the 4-position would be favorable to increase the antifungal activity. For example, compound (*Z*)-**4e** (R = *p*-Me Ph) showed higher antifungal activity against *P. piricola* than compound (*Z*)-**4g** (R = *p*-F Ph). In the Fig. 2b, the steric field contours were represented with different colors: yellow indicated that the introduction of bulky groups at 3-positions or 4-position of benzene ring was detrimental to the antifungal activity. For instance, compounds (*Z*)-**4k** (R = *p*-Cl Ph) and (*Z*)-**4n** (R = *m*-Br Ph) with a substituent group in 3-positions and 4-position showed lower antifungal activity than compounds (*Z*)-**4j** (R = *o*-Cl Ph) and (*Z*)-**4m** (R = *o*-Br Ph).

**Figure 3:**
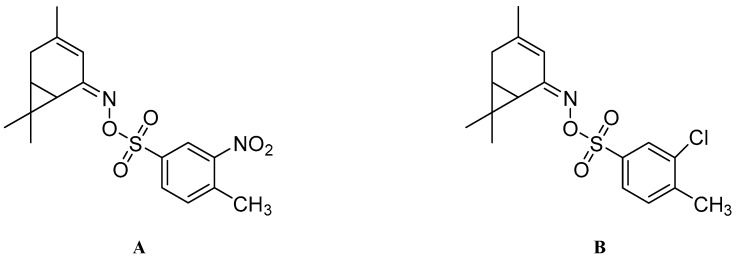
The proposed new molecules based on the established CoMFA model: (**A**) (*Z*)-3-Caren-5-one *O*-(3-nitro-4-methylbenzenesulfonyl) oxime; (**B**) (*Z*)-3-Caren-5-one *O*-(3-chloro-4-methylbenzenesulfonyl) oxime.

The established CoMFA model could be used to predict the activity of new candidate compounds prior to their synthesis. Herein, according to the results of 3D-QSAR analysis above, two new molecules with modification on phenyl gourps were proposed (Figure 3). As was expected, the designed new compounds **A** (ED’’ = −0.27) and **B** (ED’’ = −0.68) showed excellent predicted values, which indicated that the proposed structures deserves further study.

## 3. Experimental Section

### 3.1. General Information

The GC analysis was conducted on an Agilent 6890 GC (Agilent Technologies Inc., Santa Clara, CA, USA) equipped with a HP-1 column (30 m, 0.530 mm, 0.88 µm) and FID. IR spectra were recorded on a Nicolet iS50 FT-IR spectrometer (Thermo Scientific Co., Ltd., Madison, WI, USA) using the KBr pellet method. NMR spectra were recorded in CDCl_3_ solutions on a Bruker Avance III HD 600 MHz spectrometer (Bruker Co., Ltd., Zurich, Switzerland) and chemical shifts are expressed in ppm (δ) downfield relative to TMS as an internal standard. MS spectra were obtained by means of the electrospray ionization (ESI) method on TSQ Quantum Access MAX HPLC-MS instrument (Thermo Scientific Co., Ltd., Waltham, MA, USA). The UV spectra were measured on Shimadzu UV-1800 spectrophotometer (Shimadzu Corp., Kyoto, Japan). Melting points were determined on MP420 automatic melting point apparatus (Hanon Instruments Co., Ltd., Jinan, China) and were not corrected. Microwave irradiation-assisted synthesis was carried out on XO-SM50 ultrasonic microwave reaction system (Nanjing Xianou Instrument Manufacturing Co., Ltd., Nanjing, China). 3-Carene (GC purity 98%, isomeric mixture) was provided by Wuzhou Pine Chemicals Co., Ltd. (Wuzhou, Guangxi, China). Other reagents were purchased from commercial suppliers and used as received.

### 3.2. Synthesis of 3-caren-5-one *(**2**)*

3-Caren-5-one was synthesized by selective allylic oxidation of 3-carene using *tert*-butyl hydroperoxide (TBHP) as oxidant and Cu(II)HY zeolite as catalyst, according to our previous work [21]. UV-Vis (EtOH) λ_max_ (log ε): 226.50 (4.16) nm; IR (KBr, cm^−1^): 3029 (w, =C-H), 1655 (s, C=O); ^1^H- NMR (600 MHz, CDCl_3_) δ: 5.83 (s, 1H, C_4_-H), 2.64 (dd, *J* = 20.8, 8.2 Hz, 1H, C_2_-H_a_), 2.33 (d, *J* = 20.8 Hz, 1H, C_2_-H_b_), 1.87 (s, 3H, C_3_-CH_3_), 1.56 (d, *J* = 7.8 Hz, 1H, C_6_-H), 1.45 (t, *J* = 8.0 Hz, 1H, C_1_-H), 1.19 (s, 3H, C_7_-CH_3_), 1.04 (s, 3H, C_7_-CH_3_); ^13^C-NMR (150 MHz, CDCl_3_) δ: 196.67 (C_5_), 158.96 (C_3_), 126.40 (C_4_), 32.85 (C_6_), 28.43 (C_9_), 27.87 (C_2_), 25.86 (C_1_), 23.68 (C_10_), 22.57 (C_7_), 14.38 (C_8_); ESI-MS *m/z*: 151.17 [M + H]^+^.

### 3.3. Synthesis of 3-caren-5-one oxime *(**3**)*

3-Caren-5-one oxime was synthesized according to our previous work [21]. But the separation condition was improved. (*Z*)-**3a** had the highest yield when cyclohexane was screened as the crystallization solvent. 3-Caren-5-one (1.5 g, 10 mmol), NaOAc (2.41 g, 30 mmol) and NH_2_OH·HCl (1.04 g, 15 mmol) were dissolved in EtOH (25 mL) and H_2_O (5 mL). The reaction mixture was refluxed for 3 h. Upon completion, the solvent EtOH was removed by rotary evaporation, and the residue was extracted by EtOAc. The combined organic phase was washed with saturated NaCl solution three times, dried over Na_2_SO_4_, and concentrated under reduced pressure. (*Z*)-**3a** was obtained by recrystallization and filtration from cyclohexane (Yield: 68%). The filtrate was purified by silica gel column chromatography using a mixed solvent as eluent (EtOAc–petroleum ether = 1:15, *v/v*) to obtain another isomer (*E*)-**3b** (Yield: 9%).

*(Z)-3-Caren-5-one oxime* ((*Z*)-**3a**) was obtained as a white needle crystal. m.p. 96.7–98.1 °C UV-Vis (EtOH) λ_max_ (log ε): 239.70 (4.28) nm; IR (KBr, cm^−1^): 3464-3078 (s, br, O-H), 3033 (w, =C-H), 1659 (m, C=N), 1614 (m); ^1^H-NMR (600 MHz, CDCl_3_) δ: 9.03 (s, 1H, O-H), 5.87 (s, 1H, C_4_-H), 2.45 (dd, *J* = 20.0, 8.2 Hz, 1H, C_2_-H_a_), 2.12 (d, *J* = 20.0 Hz, 1H, C_2_-H_b_), 1.97 (d, *J* = 8.3 Hz, 1H, C_6_-H), 1.76 (s, 3H, C_3_-CH_3_), 1.23 (s, 3H, C_7_-CH_3_), 1.19 (t, *J* = 8.3 Hz, 1H, C_1_-H), 0.89 (s, 3H, C_7_-CH_3_); ^13^C-NMR (150 MHz, CDCl_3_) δ: 155.00 (C_5_), 142.27 (C_3_), 119.32 (C_4_), 27.97 (C_9_), 27.08 (C_2_), 23.39 (C_10_), 22.37 (C_1_), 20.71 (C_7_), 19.67 (C_6_), 14.59 (C_8_); ESI-MS *m/z*: 166.14 [M + H]^+^.

*(E)-3-Caren-5-one oxime* ((*E*)-**3b**) was obtained as a white plate-like crystal. m.p. 73.5–75.0 °C UV-Vis (EtOH) λ_max_ (log ε): 235.80 (3.89) nm; IR (KBr, cm^−1^): 3432–3081 (s, br, O-H), 3015 (w, =C-H), 1644 (m, C=N), 1608 (m); ^1^H-NMR (600 MHz, CDCl_3_) δ: 8.98 (s, 1H, O-H), 6.64 (s, 1H, C_4_-H), 2.48 (dd, *J* = 20.4, 8.0 Hz, 1H, C_2_-H_a_), 2.16 (d, *J* = 20.5 Hz, 1H, C_2_-H_b_), 1.82 (s, 3H, C_3_-CH_3_), 1.49 (d, *J* = 8.7 Hz, 1H, C_6_-H), 1.19 (t, *J* = 8.3 Hz, 1H, C_1_-H), 1.13 (s, 3H, C_7_-CH_3_), 0.88 (s, 3H, C_7_-CH_3_); ^13^C-NMR (150 MHz, CDCl_3_) δ: 152.05 (C_5_), 147.32 (C_3_), 112.59 (C_4_), 28.03 (C_9_), 27.71 (C_2_), 23.86 (C_10_), 23.54 (C_6_), 21.82 (C_1_), 19.61 (C_7_), 14.15 (C_8_); ESI-MS *m/z*: 166.14 [M + H]^+^.

### 3.4. General Procedure for Synthesis of 3-Caren-5-One Oxime Sulfonates (Z)-***4a***–***4u*** and (E)-***4v***–***4y***

A solution of sulfonyl chloride (1.1 mmol) in dry DCM (3 mL) was added slowly to a mixture of **3a** or **3b** (1 mmol) and ten drops of triethylamine in dry DCM (3 mL) with ice-bath cooling. The reaction process was monitored by TLC. Upon completion, saturated aqueous NaHCO_3_(5 mL) was added to the reaction mixture. The organic layer was separated, washed with deionized water, dried over Na_2_SO_4_, and concentrated under reduced pressure. The crude products of (*Z*)-**4a**–**4u** were further purified by silica gel chromatography (Yield: 65%–70%) and (*E*)-**4v**–**4y** were purified by recrystallization from acetone-water (Yield: 38%–44%).

*(Z)-3-Caren-5-one O-ethylsulfonyl oxime* ((*Z*)-**4a**). White solid. UV-Vis (EtOH) λ_max_ (log ε): 238.51 (4.38) nm; IR (KBr, cm^−1^): 3020 (w, =C-H), 1647 (m, C=N), 1363, 1173 (s, O=S=O); ^1^H-NMR (600 MHz, CDCl_3_) δ: 5.93 (s, 1H, C_4_-H), 3.36 (qd, *J* = 7.4, 1.7 Hz, 2H, C_11_-H), 2.54 (dd, *J* = 20.4, 8.3 Hz, 1H, C_2_-H_a_), 2.21 (d, *J* = 20.5 Hz, 1H, C_2_-H_b_), 1.98 (d, *J* = 8.1 Hz, 1H, C_6_-H), 1.83 (s, 3H, C_3_-CH_3_), 1.42 (t, *J* = 7.4 Hz, 3H, C_12_-H), 1.32 (t, *J* = 8.1 Hz, 1H, C_1_-H), 1.24 (s, 3H, C_7_-CH_3_), 0.91 (s, 3H, C_7_-CH_3_); ^13^C-NMR (150 MHz, CDCl_3_) δ: 162.82, 149.21, 117.02, 43.43, 27.99, 27.24, 23.70, 23.31, 22.37, 20.64, 14.51, 8.07; ESI-MS *m*/*z*: 258.05 [M + H]^+^.

*(Z)-3-Caren-5-one O-butylsulfonyl oxime* ((*Z*)-**4b**). Colorless syrup. UV-Vis (EtOH) λ_max_ (log ε): 238.87 (4.12) nm; IR (KBr, cm^−1^): 1649 (m, C=N), 1368, 1172 (s, O=S=O); ^1^H-NMR (600 MHz, CDCl_3_) δ: 5.94 (s, 1H, C_4_-H), 3.57–3.16 (m, 2H, C_11_-H), 2.54 (dd, *J* = 20.4, 8.3 Hz, 1H, C_2_-H_a_), 2.21 (d, *J* = 20.4 Hz, 1H, C_2_-H_b_), 1.98 (d, *J* = 8.1 Hz, 1H, C_6_-H), 1.90–1.81 (m, 5H, C_12_-H, C_3_-CH_3_), 1.53–1.43 (m, 2H, C_13_-H), 1.32 (t, *J* = 8.2 Hz, 1H, C_1_-H), 1.24 (s, 3H, C_7_-CH_3_), 0.96 (t, *J* = 7.4 Hz, 3H, C_15_-H), 0.91 (s, 3H, C_7_-CH_3_); ^13^C-NMR (150 MHz, CDCl_3_) δ: 162.72, 149.13, 117.07, 48.64, 27.99, 27.24, 25.32, 23.70, 23.31, 22.36, 21.42, 20.65, 14.51, 13.53; ESI-MS *m*/*z*: 286.10 [M + H]^+^.

*(Z)-3-Caren-5-one O-(benzenesulfonyl) oxime* ((*Z*)-**4c**). White solid. UV-Vis (EtOH) λ_max_ (log ε): 239.55 (4.45) nm; IR (KBr, cm^−1^): 3074, 3009 (w, Ar-H, =C-H), 1652, 1575, 1476 (m, C=N, Ar), 1371, 1192 (s, O=S=O); ^1^H-NMR (600 MHz, CDCl_3_) δ: 7.99 (d, *J* = 8.0 Hz, 2H, C_12_-H, C_16_-H), 7.63 (t, *J* = 7.4 Hz, 1H, C_14_-H), 7.53 (t, *J* = 7.8 Hz, 2H, C_13_-H, C_15_-H), 5.83 (s, 1H, C_4_-H), 2.47 (dd, *J* = 20.4, 8.3 Hz, 1H, C_2_-H_a_), 2.13 (d, *J* = 20.4 Hz, 1H, C_2_-H_b_), 1.92 (d, *J* = 8.1 Hz, 1H, C_6_-H), 1.76 (s, 3H, C_3_-CH_3_), 1.25 (t, *J* = 8.2 Hz, 1H, C_1_-H), 1.21 (s, 3H, C_7_-CH_3_), 0.73 (s, 3H, C_7_-CH_3_); ^13^C-NMR (150 MHz, CDCl_3_) δ: 162.85, 148.89, 136.18, 133.59, 128.84, 128.68, 117.06, 27.93, 27.15, 23.63, 23.19, 22.19, 20.60, 14.26; ESI-MS *m*/*z*: 306.06 [M + H]^+^.

*(Z)-3-Caren-5-one O-(2-methylbenzenesulfonyl) oxime* ((*Z*)-**4d**). Colorless syrup. UV-Vis (EtOH) λ_max_ (log ε): 238.74 (4.38) nm; IR (KBr, cm^−1^): 3066, 3015 (w, Ar-H, =C-H), 1652, 1578 1476 (m, C=N, Ar), 1365, 1186 (s, O=S=O); ^1^H-NMR (600 MHz, CDCl_3_) δ: 8.06 (dd, *J* = 7.9, 1.1 Hz, 1H, Ar-H), 7.49 (td, *J* = 7.5, 1.2 Hz, 1H, Ar-H), 7.36–7.32 (m, 2H, Ar-H), 5.77 (s, 1H, C_4_-H), 2.68 (s, 3H, C_12_-CH_3_), 2.48 (dd, *J* = 20.5, 8.3 Hz, 1H, C_2_-H_a_), 2.14 (d, *J* = 20.4 Hz, 1H, C_2_-H_b_), 1.98 (d, *J* = 8.1 Hz, 1H, C_6_-H), 1.74 (s, 3H, C_3_-CH_3_), 1.28 (t, *J* = 8.1 Hz, 1H, C_1_-H), 1.24 (s, 3H, C_7_-CH_3_), 0.79 (s, 3H, C_7_-CH_3_); ^13^C-NMR (150 MHz, CDCl3) δ: 162.22, 148.60, 138.23, 134.74, 133.59, 132.17, 131.01, 126.07, 117.16, 27.95, 27.10, 23.58, 23.15, 22.13, 20.54, 20.43, 14.37; ESI-MS *m*/*z*: 320.06 [M + H]^+^.

*(Z)-3-Caren-5-one O-(4-methylbenzenesulfonyl) oxime* ((*Z*)-**4e**). White solid. UV-Vis (EtOH) λ_max_ (log ε): 232.25 (4.36) nm; IR (KBr, cm^−1^): 3072, 3021 (w, Ar-H, =C-H), 1649, 1598, 1575, 1450 (m, C=N, Ar), 1365, 1186 (s, O=S=O); ^1^H-NMR (600 MHz, CDCl_3_) δ: 7.87 (d, *J* = 8.3 Hz, 2H, C_12_-H, C_16_-H), 7.32 (d, *J* = 8.0 Hz, 2H, C_13_-H, C_15_-H), 5.83 (s, 1H, C_4_-H), 2.46 (dd, *J* = 20.4, 8.3 Hz, 1H, C_2_-H_a_), 2.43 (s, 3H, C_14_-CH_3_), 2.13 (d, *J* = 20.4 Hz, 1H, C_2_-H_b_), 1.92 (d, *J* = 8.1 Hz, 1H, C_6_-H), 1.76 (s, 3H, C_3_-CH_3_), 1.25 (t, *J* = 8.2 Hz, 1H, C_1_-H), 1.21 (s, 3H, C_7_-CH_3_), 0.74 (s, 3H, C_7_-CH_3_); ^13^C-NMR (150 MHz, CDCl_3_) δ: 162.68, 148.67, 144.56, 133.18, 129.48, 128.72, 117.18, 27.94, 27.15, 23.62, 23.17, 22.15, 21.67, 20.60, 14.28; ESI-MS *m*/*z*: 320.07 [M + H]^+^.

*(Z)-3-Caren-5-one O-(2,5-dimethylbenzenesulfonyl) oxime* ((*Z*)-**4f**). White solid. UV-Vis (EtOH) λ_max_ (log ε): 236.42 (4.43) nm; IR (KBr, cm^−1^): 3027, 3008 (w, Ar-H, =C-H), 1643, 1578, 1500, 1453 (m, C=N, Ar), 1361, 1175 (s, O=S=O); ^1^H-NMR (600 MHz, CDCl_3_) δ: 7.88 (s, 1H, C_16_-H), 7.29 (d, *J* = 7.7 Hz, 1H, Ar-H), 7.20 (d, *J* = 7.7 Hz, 1H, Ar-H), 5.79 (s, 1H, C_4_-H), 2.62 (s, 3H, C_12_-H), 2.48 (dd, *J* = 20.4, 8.4 Hz, 1H, C_2_-H_a_), 2.38 (s, 3H, C_15_-H), 2.14 (d, *J* = 20.5 Hz, 1H, C_2_-H_b_), 1.98 (d, *J* = 8.1 Hz, 1H, C_6_-H), 1.75 (s, 3H, C_3_-CH_3_), 1.28 (t, *J* = 8.2 Hz, 1H, C_1_-H), 1.24 (s, 3H, C_7_-CH_3_), 0.80 (s, 3H, C_7_-CH_3_); ^13^C-NMR (150 MHz, CDCl_3_) δ: 162.14, 148.51, 135.99, 135.00, 134.35, 132.08, 131.29, 117.22, 27.94, 27.10, 23.58, 23.13, 22.10, 20.77, 20.52, 19.91, 14.37; ESI-MS *m*/*z*: 334.07 [M + H]^+^.

*(Z)-3-Caren-5-one O-(4-fluorobenzenesulfonyl) oxime* ((*Z*)-**4g**). White solid. UV-Vis (EtOH) λ_max_ (log ε): 239.71 (4.34) nm; IR (KBr, cm^−1^): 3077, 3018 (w, Ar-H, =C-H), 1658, 1595, 1493 (m, C=N, Ar), 1374, 1192 (s, O=S=O); ^1^H-NMR (600 MHz, CDCl_3_) δ: 8.01 (dd, *J* = 8.7, 5.1 Hz, 2H, C_12_-H, C_16_-H), 7.21 (t, *J* = 8.5 Hz, 2H, C_13_-H, C_15_-H), 5.82 (s, 1H, C_4_-H), 2.48 (dd, *J* = 20.5, 8.3 Hz, 1H, C_2_-H_a_), 2.15 (d, *J* = 20.4 Hz, 1H, C_2_-H_b_), 1.92 (d, *J* = 8.1 Hz, 1H, C_6_-H), 1.78 (s, 3H, C_3_-CH_3_), 1.27 (t, *J* = 8.2 Hz, 1H, C_1_-H), 1.22 (s, 3H, C_7_-CH_3_), 0.76 (s, 3H, C_7_-CH_3_); ^13^C-NMR (150 MHz, CDCl_3_) δ: 166.59, 164.90, 162.98, 149.17, 132.15, 132.13, 131.62, 131.55, 116.94, 116.24, 116.09, 27.95, 27.17, 23.65, 23.26, 22.28, 20.64, 14.33; ESI-MS *m*/*z*: 324.05 [M + H]^+^.

*(Z)-3-Caren-5-one O-(2,4-difluorobenzenesulfonyl) oxime* ((*Z*)-**4h**). White solid. UV-Vis (EtOH) λ_max_ (log ε): 239.21 (4.30) nm; IR (KBr, cm^−1^): 3060, 3020 (w, Ar-H, =C-H), 1658, 1601, 1485 (m, C=N, Ar), 1360, 1192 (s, O=S=O); 1078, 976 (s, Ar-F); ^1^H-NMR (600 MHz, CDCl_3_) δ: 8.01 (td, *J* = 8.2, 6.2 Hz, 1H, Ar-H), 7.03 (td, *J* = 8.8, 2.0 Hz, 1H, Ar-H), 7.00–6.92 (m, 1H, Ar-H), 5.78 (s, 1H, C_4_-H), 2.50 (dd, *J* = 20.5, 8.3 Hz, 1H, C_2_-H_a_), 2.16 (d, *J* = 20.5 Hz, 1H, C_2_-H_b_), 1.99 (d, *J* = 8.1 Hz, 1H, C_6_-H), 1.77 (s, 3H, C_3_-CH_3_), 1.31 (t, *J* = 8.2 Hz, 1H, C_1_-H), 1.25 (s, 3H, C_7_-CH_3_), 0.82 (s, 3H, C_7_-CH_3_); ^13^C-NMR (150 MHz, CDCl_3_) δ: 167.43, 167.36, 165.72, 165.65, 163.31, 161.06, 160.97, 159.32, 159.24, 149.61, 133.89, 133.82, 120.74, 120.65, 116.70, 112.00, 111.98, 111.85, 111.83, 105.86, 105.69, 105.52, 27.88, 27.19, 23.67, 23.29, 22.42, 20.63, 14.34; ESI-MS *m*/*z*: 342.04 [M + H]^+^.

*(Z)-3-Caren-5-one O-(2,3,4,5,6-pentafluorobenzenesulfonyl) oxime* ((*Z*)-**4i**). Colorless syrup. UV-Vis (EtOH) λ_max_ (log ε): 239.30 (4.41) nm; IR (KBr, cm^−1^): 3011 (w, =C-H), 1646, 1524, 1507 (m, C=N, Ar), 1385, 1201 (s, O=S=O); ^1^H-NMR (600 MHz, CDCl_3_) δ: 5.81 (s, 1H, C_4_-H), 2.54 (dd, *J* = 20.6, 8.4 Hz, 1H, C_2_-H_a_), 2.21 (d, *J* = 20.6 Hz, 1H, C_2_-H_b_), 1.98 (d, *J* = 8.0 Hz, 1H, C_6_-H), 1.81 (s, 3H, C_3_-CH_3_), 1.36 (t, *J* = 8.2 Hz, 1H, C_1_-H), 1.27 (s, 3H, C_7_-CH_3_), 0.87 (s, 3H, C_7_-CH_3_); ^13^C-NMR (150 MHz, CDCl_3_) δ: 164.27, 150.82, 146.13, 144.44, 143.91, 145.66, 138.74, 137.03, 116.21, 112.55, 27.89, 27.28, 23.75, 23.52, 22.87, 20.81, 14.36; ESI-MS *m*/*z*: 395.99 [M + H]^+^.

*(Z)-3-Caren-5-one O-(2-chlorobenzenesulfonyl) oxime* ((*Z*)-**4j**). White solid. UV-Vis (EtOH) λ_max_ (log ε): 237.84 (4.44) nm; IR (KBr, cm^−1^): 3094, 3032 (w, Ar-H, =C-H), 1649, 1573, 1456 (m, C=N, Ar), 1371, 1192 (s, O=S=O); ^1^H-NMR (600 MHz, CDCl_3_) δ: 8.22–8.12 (m, 1H, Ar-H), 7.59–7.51 (m, 2H, Ar-H), 7.47–7.40 (m, 1H, Ar-H), 5.76 (s, 1H, C_4_-H), 2.49 (dd, *J* = 20.5, 8.4 Hz, 1H, C_2_-H_a_), 2.15 (d, *J* = 20.5 Hz, 1H, C_2_-H_b_), 2.05 (d, *J* = 8.1 Hz, 1H, C_6_-H), 1.75 (s, 3H, C_3_-CH_3_), 1.30 (t, *J* = 8.0 Hz, 1H, C_1_-H), 1.26 (s, 3H, C_7_-CH_3_), 0.83 (s, 3H, C_7_-CH_3_); ^13^C-NMR (150 MHz, CDCl_3_) δ: 162.75, 149.13, 134.52, 134.24, 132.87, 132.69, 131.73, 126.90, 116.87, 27.94, 27.17, 23.62, 23.21, 22.35, 20.58, 14.35; ESI-MS *m*/*z*: 340.00 [M + H]^+^.

*(Z)-3-Caren-5-one O-(4-chlorobenzenesulfonyl) oxime* ((*Z*)-**4k**). White solid. UV-Vis (EtOH) λ_max_ (log ε): 233.23 (4.44) nm; IR (KBr, cm^−1^): 3072, 3026 (w, Ar-H, =C-H), 1652, 1590, 1578, 1481 (m, C=N, Ar), 1377, 1195 (s, O=S=O); ^1^H-NMR (600 MHz, CDCl_3_) δ: 7.93 (d, *J* = 8.7 Hz, 2H, C_12_-H, C_16_-H), 7.51 (d, *J* = 8.7 Hz, 2H, C_13_-H, C_15_-H), 5.82 (s, 1H, C_4_-H), 2.48 (dd, *J* = 20.4, 8.3 Hz, 1H, C_2_-H_a_), 2.15 (d, *J* = 20.5 Hz, 1H, C_2_-H_b_), 1.91 (d, *J* = 8.1 Hz, 1H, C_6_-H), 1.78 (s, 3H, C_3_-CH_3_), 1.27 (t, *J* = 8.2 Hz, 1H, C_1_-H), 1.22 (s, 3H, C_7_-CH_3_), 0.77 (s, 3H, C_7_-CH_3_); ^13^C-NMR (150 MHz, CDCl_3_) δ: 163.06, 149.26, 140.27, 134.60, 130.20, 129.18, 116.89, 27.96, 27.17, 23.65, 23.28, 22.32, 20.65, 14.33; ESI-MS *m*/*z*: 340.01 [M + H]^+^.

*(Z)-3-Caren-5-one O-(3,5-dichlorobenzenesulfonyl) oxime* ((*Z*)-**4l**). White solid. UV-Vis (EtOH) λ_max_ (log ε): 236.05 (4.41) nm; IR (KBr, cm^−1^): 3077, 3023 (w, Ar-H, =C-H), 1649, 1573, 1445 (m, C=N, Ar), 1377, 1195 (s, O=S=O); ^1^H-NMR (600 MHz, CDCl_3_) δ: 7.86 (d, *J* = 1.8 Hz, 2H, C_12_-H, C_16_-H), 7.60 (t, *J* = 1.8 Hz, 1H, C_14_-H), 5.84 (s, 1H, C_4_-H), 2.51 (dd, *J* = 20.5, 8.3 Hz, 1H, C_2_-H_a_), 2.17 (d, *J* = 20.5 Hz, 1H, C_2_-H_b_), 1.93 (d, *J* = 8.1 Hz, 1H, C_6_-H), 1.80 (s, 3H, C_3_-CH_3_), 1.31 (t, *J* = 8.1 Hz, 1H, C_1_-H), 1.24 (s, 3H, C_7_-CH_3_), 0.81 (s, 3H, C_7_-CH_3_); ^13^C-NMR (150 MHz, CDCl_3_) δ: 163.61, 149.86, 138.89, 135.79, 133.60, 127.12, 116.64, 27.98, 27.22, 23.69, 23.40, 22.55, 20.73, 14.33; ESI-MS *m*/*z*: 373.97, 375.96 [M + H]^+^.

*(Z)-3-Caren-5-one O-(2-bromobenzenesulfonyl) oxime* ((*Z*)-**4m**). White solid. UV-Vis (EtOH) λ_max_ (log ε): 230.85 (4.42) nm; IR (KBr, cm^−1^): 3086, 3029 (w, Ar-H, =C-H), 1649, 1578, 1450 (m, C=N, Ar), 1368, 1195 (s, O=S=O); ^1^H-NMR (600 MHz, CDCl_3_) δ: 8.19 (dd, *J* = 7.8, 1.8 Hz, 1H, Ar-H), 7.75 (dd, *J* = 7.8, 1.3 Hz, 1H, Ar-H), 7.47 (m, 2H, Ar-H), 5.75 (s, 1H, C_4_-H), 2.49 (dd, *J* = 20.5, 8.4 Hz, 1H, C_2_-H_a_), 2.16 (d, *J* = 20.5 Hz, 1H, C_2_-H_b_), 2.07 (d, *J* = 8.1 Hz, 1H, C_6_-H), 1.75 (s, 3H, C_3_-CH_3_), 1.30 (t, *J* = 8.1 Hz, 1H, C_1_-H), 1.26 (s, 3H, C_7_-CH_3_), 0.85 (s, 3H, C_7_-CH_3_); ^13^C-NMR (150 MHz, CDCl_3_) δ: 162.66, 149.09, 136.07, 135.28, 134.44, 133.16, 127.47, 120.58, 116.89, 28.00, 27.17, 23.62, 23.21, 22.36, 20.60, 14.41; ESI-MS *m*/*z*: 383.95, 385.95 [M + H]^+^.

*(Z)-3-Caren-5-one O-(3-bromobenzenesulfonyl) oxime* ((*Z*)-**4n**). White solid. UV-Vis (EtOH) λ_max_ (log ε): 229.30 (4.43) nm; IR (KBr, cm^−1^): 3077, 3023 (w, Ar-H, =C-H), 1658, 1575, 1462 (m, C=N, Ar), 1374, 1189 (s, O=S=O); ^1^H-NMR (600 MHz, CDCl_3_) δ: 8.13 (t, *J* = 1.8 Hz, 1H, Ar-H), 7.96–7.89 (m, 1H, Ar-H), 7.75 (ddd, *J* = 8.0, 1.8, 1.0 Hz, 1H, Ar-H), 7.42 (t, *J* = 8.0 Hz, 1H, C_15_-H), 5.83 (s, 1H, C_4_-H), 2.49 (dd, *J* = 20.5, 8.3 Hz, 1H, C_2_-H_a_), 2.15 (d, *J* = 20.5 Hz, 1H, C_2_-H_b_), 1.93 (d, *J* = 8.1 Hz, 1H, C_6_-H), 1.78 (s, 3H, C_3_-CH_3_), 1.28 (t, *J* = 8.1 Hz, 1H, C_1_-H), 1.23 (s, 3H, C_7_-CH_3_), 0.77 (s, 3H, C_7_-CH_3_); ^13^C-NMR (150 MHz, CDCl_3_) δ: 163.25, 149.39, 137.94, 136.64, 131.53, 130.33, 127.32, 122.75, 116.84, 27.96, 27.18, 23.66, 23.31, 22.38, 20.67, 14.30; ESI-MS *m*/*z*: 383.95, 385.96 [M + H]^+^.

*(Z)-3-Caren-5-one O-(4-bromobenzenesulfonyl) oxime* ((*Z*)-**4o**). White solid. UV-Vis (EtOH) λ_max_ (log ε): 237.90 (4.54) nm; IR (KBr, cm^−1^): 3069, 3026 (w, Ar-H, =C-H), 1652, 1575, 1476 (m, C=N, Ar), 1377, 1195 (s, O=S=O); ^1^H-NMR (600 MHz, CDCl_3_) δ: 7.85 (d, *J* = 8.7 Hz, 2H, C_12_-H, C_16_-H), 7.68 (d, *J* = 8.7 Hz, 2H, C_13_-H, C_15_-H), 5.82 (s, 1H, C_4_-H), 2.48 (dd, *J* = 20.5, 8.3 Hz, 1H, C_2_-H_a_), 2.15 (d, *J* = 20.6 Hz, 1H, C_2_-H_b_), 1.91 (d, *J* = 8.1 Hz, 1H, C_6_-H), 1.78 (s, 3H, C_3_-CH_3_), 1.27 (t, *J* = 8.1 Hz, 1H, C_1_-H), 1.22 (s, 3H, C_7_-CH_3_), 0.77 (s, 3H, C_7_-CH_3_); ^13^C-NMR (150 MHz, CDCl_3_) δ: 163.07, 149.28, 135.14, 132.1, 130.26, 128.857, 116.88, 27.96, 27.18, 23.65, 23.28, 22.33, 20.65, 14.34; ESI-MS *m*/*z*: 383.99, 385.98 [M + H]^+^.

*(Z)-3-Caren-5-one O-(3-nitrobenzenesulfonyl) oxime* ((*Z*)-**4p**). White solid. UV-Vis (EtOH) λ_max_ (log ε): 242.29 (4.58) nm; IR (KBr, cm^−1^): 3097,3023 (w, Ar-H, =C-H), 1652,1612, 1578, 1468 (m, C=N, Ar), 1536, 1348 (s, N=O), 1379, 1195 (s, O=S=O); ^1^H NMR (600 MHz, CDCl_3_) δ: 8.84 (s, 1H, Ar-H), 8.52–8.47 (m, 1H, Ar-H), 8.32 (d, *J* = 7.8 Hz, 1H, Ar-H), 7.77 (t, *J* = 8.0 Hz, 1H, Ar-H), 5.81 (s, 1H, C_4_-H), 2.50 (dd, *J* = 20.5, 8.4 Hz, 1H, C_2_-H_a_), 2.17 (d, *J* = 20.5 Hz, 1H, C_2_-H_b_), 1.94 (d, *J* = 8.1 Hz, 1H, C_6_-H), 1.79 (s, 3H, C_3_-CH_3_), 1.31 (t, *J* = 8.2 Hz, 1H, C_1_-H), 1.25 (s, 3H, C_7_-CH_3_), 0.80 (s, 3H, C_7_-CH_3_); ^13^C-NMR (150 MHz, CDCl_3_) δ: 163.67, 150.02, 148.16, 138.22, 134.33, 130.20, 128.05, 124.18, 116.54, 27.97, 27.22, 23.69, 23.43, 22.60, 20.75, 14.36; ESI-MS *m*/*z*: 351.02 [M + H]^+^.

*(Z)-3-Caren-5-one O-(4-*cyano*benzenesulfonyl) oxime* ((*Z*)-**4q**). White solid. UV-Vis (EtOH) λ_max_ (log ε): 235.02 (4.54) nm; IR (KBr, cm^−1^): 3094, 3043 (w, Ar-H, =C-H), 2231 (s, C≡N), 1652,1567, 1442 (m, C=N, Ar), 1377, 1195 (s, O=S=O); ^1^H-NMR (600 MHz, CDCl_3_) δ: 8.11 (d, *J* = 8.5 Hz, 2H, C_13_-H, C_15_-H), 7.84 (d, *J* = 8.5 Hz, 2H, C_12_-H, C_16_-H), 5.80 (s, 1H, C_4_-H), 2.50 (dd, *J* = 20.5, 8.3 Hz, 1H, C_2_-H_a_), 2.17 (d, *J* = 20.5 Hz, 1H, C_2_-H_b_), 1.91 (d, *J* = 8.1 Hz, 1H, C_6_-H), 1.79 (s, 3H, C_3_-CH_3_), 1.30 (t, *J* = 8.2 Hz, 1H, C_1_-H), 1.23 (s, 3H, C_7_-CH_3_), 0.78 (s, 3H, C_7_-CH_3_); ^13^C-NMR (150 MHz, CDCl_3_) δ: 163.54, 149.93, 140.33, 132.61, 129.46, 117.31, 117.24, 116.55, 27.96, 27.20, 23.69, 23.38, 22.52, 20.70, 14.37; ESI-MS *m*/*z*: 331.05 [M + H]^+^.

*(Z)-3-Caren-5-one O-(4-methoxybenzenesulfonyl) oxime* ((*Z*)-**4r**). Colorless syrup. UV-Vis (EtOH) λ_max_ (log ε): 243.61 (4.70) nm; IR (KBr, cm^−1^): 3012 (w, =C-H), 1652, 1598, 1578, 1499 (m, C=N, Ar), 1368, 1195 (s, O=S=O), 1266 (s, C-O-C); ^1^H-NMR (600 MHz, CDCl_3_) δ: 7.92 (dd, *J* = 8.9, 1.5 Hz, 2H, C_12_-H, C_16_-H), 6.99 (d, *J* = 8.9 Hz, 2H, C_13_-H, C_15_-H), 5.83 (s, 1H, C_4_-H), 3.87 (s, 3H, C_17_-H), 2.47 (dd, *J* = 20.4, 8.3 Hz, 1H, C_2_-H_a_), 2.13 (d, *J* = 20.4 Hz, 1H, C_2_-H_b_), 1.92 (d, *J* = 8.1 Hz, 1H, C_6_-H), 1.77 (s, 3H, C_3_-CH_3_), 1.25 (t, *J* = 8.1 Hz, 1H, C_1_-H), 1.21 (s, 3H, C_7_-CH_3_), 0.75 (s, 3H, C_7_-CH_3_); ^13^C-NMR (150 MHz, CDCl_3_) δ: 163.68, 162.57, 148.58, 130.92, 127.60, 117.24, 114.06, 55.65, 27.95, 27.15, 23.62, 23.17, 22.13, 20.60, 14.32; ESI-MS *m*/*z*: 336.06 [M + H]^+^.

*(Z)-3-Caren-5-one O-(4-chloro-3-nitrobenzenesulfonyl) oxime* ((*Z*)-**4s**). White solid. UV-Vis (EtOH) λ_max_ (log ε): 225.14 (4.48) nm; IR (KBr, cm^−1^): 3080, 3040 (w, Ar-H, =C-H), 1652, 1595, 1573, 1453 (m, C=N, Ar), 1538, 1348 (s, N=O), 1388, 1195 (s, O=S=O); ^1^H-NMR (600 MHz, CDCl_3_) δ: 8.49 (d, *J* = 2.1 Hz, 1H, Ar-H), 8.12 (dd, *J* = 8.5, 2.1 Hz, 1H, Ar-H), 7.75 (d, *J* = 8.5 Hz, 1H, Ar-H), 5.81 (s, 1H, C_4_-H), 2.51 (dd, *J* = 20.5, 8.3 Hz, 1H, C_2_-H_a_), 2.18 (d, *J* = 20.5 Hz, 1H, C_2_-H_b_), 1.92 (d, *J* = 8.1 Hz, 1H, C_6_-H), 1.81 (s, 3H, C_3_-CH_3_), 1.32 (t, *J* = 8.2 Hz, 1H, C_1_-H), 1.24 (s, 3H, C_7_-CH_3_), 0.83 (s, 3H, C_7_-CH_3_); ^13^C-NMR (150 MHz, CDCl_3_) δ: 163.83, 150.29, 147.72, 136.20, 132.83–132.59, 126.25, 116.43, 27.97, 27.25, 23.71, 23.49, 22.71, 20.78, 14.39; ESI-MS *m*/*z*: 384.98 [M + H]^+^.

*(Z)-3-Caren-5-one O-(γ-biphenylsulfonyl) oxime* ((*Z*)-**4t**). White solid. UV-Vis (EtOH) λ_max_ (log ε): 258.79 (4.44) nm; IR (KBr, cm^−1^): 3067, 3037 (w, Ar-H, =C-H), 1649, 1592, 1482, 1451 (m, C=N, Ar), 1371, 1184 (s, O=S=O); ^1^H-NMR (600 MHz, CDCl_3_) δ: 8.05 (d, *J* = 8.4 Hz, 2H, Ar-H), 7.73 (d, *J* = 8.4 Hz, 2H, Ar-H), 7.61 (d, *J* = 7.4 Hz, 2H, Ar-H), 7.48 (t, *J* = 7.6 Hz, 2H, Ar-H), 7.42 (t, *J* = 7.3 Hz, 1H, Ar-H), 5.86 (s, 1H, C_4_-H), 2.47 (dd, *J* = 20.5, 8.3 Hz, 1H, C_2_-H_a_), 2.14 (d, *J* = 20.5 Hz, 1H, C_2_-H_b_), 1.95 (d, *J* = 8.1 Hz, 1H, C_6_-H), 1.77 (s, 3H, C_3_-CH_3_), 1.26 (t, *J* = 8.1 Hz, 1H, C_1_-H), 1.22 (s, 3H, C_7_-CH_3_), 0.76 (s, 3H, C_7_-CH_3_); ^13^C- NMR (150 MHz, CDCl_3_) δ: 162.93, 148.91, 146.52, 139.28, 134.68, 129.23, 129.06, 128.59, 127.47, 127.38, 117.11, 27.96, 27.17, 23.64, 23.23, 22.24, 20.65, 14.30; ESI-MS *m*/*z*: 382.06 [M + H]^+^.

*(Z)-3-Caren-5-one O-(α-thienylsulfonyl) oxime* ((*Z*)-**4u**). White solid. UV-Vis (EtOH) λ_max_ (log ε): 239.91 (4.45) nm; IR (KBr, cm^−1^): 3094, 3012 (w, Ar-H, =C-H), 1655, 1575, 1504, (m, C=N, Ar), 1371, 1181 (s, O=S=O); ^1^H-NMR (600 MHz, CDCl_3_) δ: 7.80 (dd, *J* = 3.8, 1.3 Hz, 1H, C_14_-H), 7.69 (dd, *J* = 5.0, 1.3 Hz, 1H, C_12_-H), 7.12 (dd, *J* = 4.9, 3.9 Hz, 1H, C_13_-H), 5.89 (s, 1H, C_4_-H), 2.49 (dd, *J* = 20.4, 8.3 Hz, 1H, C_2_-H_a_), 2.16 (d, *J* = 20.4 Hz, 1H, C_2_-H_b_), 1.92 (d, *J* = 8.1 Hz, 1H, C_6_-H), 1.80 (s, 3H, C_3_-CH_3_), 1.27 (t, *J* = 8.2 Hz, 1H, C_1_-H), 1.21 (s, 3H, C_7_-CH_3_), 0.78 (s, 3H, C_7_-CH_3_); ^13^C NMR (150 MHz, CDCl_3_) δ: 163.03, 149.23, 135.63, 134.83, 133.76, 127.12, 116.96, 27.90, 27.19, 23.67, 23.26, 22.31, 20.64, 14.30; ESI-MS *m*/*z*: 312.01 [M + H]^+^.

*(E)-3-Caren-5-one O-(benzenesulfonyl) oxime* ((*E*)-**4v**). White solid. UV-Vis (EtOH) λ_max_ (log ε): 227.27 (4.26) nm; IR (KBr, cm^−1^): 3066, 3026 (w, Ar-H, =C-H), 1641, 1573, 1473 (m, C=N, Ar), 1371, 1189 (s, O=S=O); ^1^H-NMR (600 MHz, CDCl_3_) δ: 7.99 (d, *J* = 8.1 Hz, 2H, C_12_-H, C_16_-H), 7.63 (t, *J* = 7.5 Hz, 1H, C_14_-H), 7.53 (t, *J* = 7.8 Hz, 2H, C_13_-H, C_15_-H), 6.49 (s, 1H, C_4_-H), 2.49 (dd, *J* = 20.9, 8.0 Hz, 1H, C_2_-H_a_), 2.19 (d, *J* = 20.8 Hz, 1H, C_2_-H_b_), 1.84 (s, 3H, C_3_-CH_3_), 1.52 (d, *J* = 8.5 Hz, 1H, C_6_-H), 1.24 (t, *J* = 8.3 Hz, 1H, C_1_-H), 1.10 (s, 3H, C_7_-CH_3_), 0.77 (s, 3H, C_7_-CH_3_); ^13^C-NMR (150 MHz, CDCl_3_) δ: 160.14, 153.75, 136.22, 133.52, 128.81, 128.67, 112.57, 28.01, 27.91, 24.00, 23.33, 22.45, 20.51, 13.91; ESI-MS *m*/*z*: 306.07 [M + H]^+^.

*(E)-3-Caren-5-one O-(4-methylbenzenesulfonyl) oxime* ((*E*)-**4w**). White solid. UV-Vis (EtOH) λ_max_ (log ε): 228.22 (4.10) nm; IR (KBr, cm^−1^): 3097, 3046 (w, Ar-H, =C-H), 1631, 1598, 1442 (m, C=N, Ar), 1370, 1189 (s, O=S=O); ^1^H-NMR (600 MHz, CDCl_3_) δ: 7.87 (d, *J* = 8.2 Hz, 2H, C_12_-H, C_16_-H), 7.32 (d, *J* = 8.2 Hz, 2H, C_13_-H, C_15_-H), 6.49 (s, 1H, C_4_-H), 2.48 (dd, *J* = 20.8, 8.0 Hz, 1H, C_2_-H_a_), 2.43 (s, 3H, C_14_-CH_3_), 2.18 (d, *J* = 20.8 Hz, 1H, C_2_-H_b_), 1.83 (s, 3H, C_3_-CH_3_), 1.53 (d, *J* = 8.5 Hz, 1H, C_6_-H), 1.24 (t, *J* = 8.3 Hz, 1H, C_1_-H), 1.10 (s, 3H, C_7_-CH_3_), 0.78 (s, 3H, C_7_-CH_3_); ^13^C-NMR (150 MHz, CDCl_3_) δ: 160.02, 153.50, 144.47, 133.23, 129.45, 128.70, 112.64, 28.01, 27.93, 23.98, 23.39, 22.44, 21.67, 20.49, 13.92; ESI-MS *m*/*z*: 320.07 [M + H]^+^.

*(E)-3-Caren-5-one O-(4-chlorobenzenesulfonyl) oxime* ((*E*)-**4x**). White solid. UV-Vis (EtOH) λ_max_ (log ε): 229.87 (4.36) nm; IR (KBr, cm^−1^): 3097, 3026 (w, Ar-H, =C-H), 1638, 1590, 1573, 1478 (m, C=N, Ar), 1374, 1195 (s, O=S=O); ^1^H-NMR (600 MHz, CDCl_3_) δ: 7.93 (d, *J* = 8.6 Hz, 2H, C_12_-H, C_16_-H), 7.51 (d, *J* = 8.6 Hz, 2H, C_13_-H, C_15_-H), 6.48 (s, 1H, C_4_-H), 2.50 (dd, *J* = 20.8, 8.0 Hz, 1H, C_2_-H_a_), 2.20 (d, *J* = 20.9 Hz, 1H, C_2_-H_b_), 1.85 (s, 3H, C_3_-CH_3_), 1.52 (d, *J* = 8.5 Hz, 1H, C_6_-H), 1.26 (t, *J* = 8.3 Hz, 1H, C_1_-H), 1.11 (s, 3H, C_7_-CH_3_), 0.80 (s, 3H, C_7_-CH_3_); ^13^C-NMR (150 MHz, CDCl_3_) δ: 160.37, 154.16, 140.19, 134.66, 130.19, 129.16, 112.48, 28.05, 27.92, 24.02, 23.38, 22.53, 20.61, 13.93; ESI-MS *m*/*z*: 339.99 [M + H]^+^.

*(E)-3-Caren-5-one O-(4-bromobenzenesulfonyl) oxime* ((*E*)-**4y**). White solid. UV-Vis (EtOH) λ_max_ (log ε): 238.46 (4.54) nm; IR (KBr, cm^−1^): 3097, 3026 (w, Ar-H, =C-H), 1641, 1575, 1476 (m, C=N, Ar), 1377, 1195 (s, O=S=O); ^1^H-NMR (600 MHz, CDCl_3_) δ: 7.85 (d, *J* = 8.7 Hz, 2H, C_12_-H, C_16_-H), 7.67 (d, *J* = 8.7 Hz, 2H, C_13_-H, C_15_-H), 6.48 (s, 1H, C_4_-H), 2.50 (dd, *J* = 20.9, 8.1 Hz, 1H, C_2_-H_a_), 2.20 (d, *J* = 20.9 Hz, 1H, C_2_-H_b_), 1.85 (s, 3H, C_3_-CH_3_), 1.52 (d, *J* = 8.5 Hz, 1H, C_6_-H), 1.26 (t, *J* = 8.2 Hz, 1H, C_1_-H), 1.11 (s, 3H, C_7_-CH_3_), 0.80 (s, 3H, C_7_-CH_3_); ^13^C-NMR (150 MHz, CDCl_3_) δ: 160.38, 154.15, 135.21, 132.14, 130.25, 128.76, 112.48, 28.06, 27.93, 24.02, 23.38, 22.53, 20.62, 13.93; ESI-MS *m*/*z*: 383.95, 385.98 [M + H]^+^.

### 3.5. Antifungal Activity Test

In vitro method was employed to evaluate antifungal activity of the target compounds [21]. The test compound was dissolved in acetone and then diluted to a 500 µg/mL drug solution with 200 µg/mL of sorporl-144 emulsifier. The flat containing 50 µg/mL tested compound was prepared using 1 mL of drug solution and 9 mL of PSA medium. Three bacterium trays with a diameter of 4 mm were placed in the flat containing the tested compound. After each treatment was cultured in a 24 ± 1 °C for 48 h, the expanded diameter of the bacterium tray was measured. Compared with the positive control, the relative inhibition percentage was calculated. Activity grading indicators: Grade A: ≥90%; Grade B: 70~90%; Grade C: 50~70%; Grade D: <50%.

### 3.6. 3D-QSAR Analysis

Molecular modeling was performed using SYBYL-X 2.1.1 software (Tripos, Inc., St. Louis, MO, USA). According to literature reports [28], the antifungal activity against *P. piricola* was expressed in terms of activity factor (ED) by the formula:

ED = log {*I*/[(100 − *I*) × *M*_W_]}

where *I* was the percent inhibition at 50 µg/mL and *M*_W_ was the molecular weight of the tested compounds.

**Figure 4:**
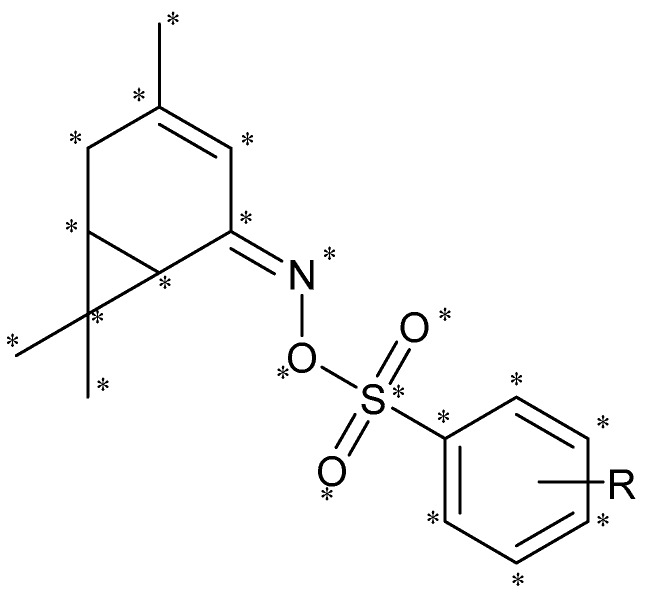
The asterisk skeleton of title compounds.

**Figure 5:**
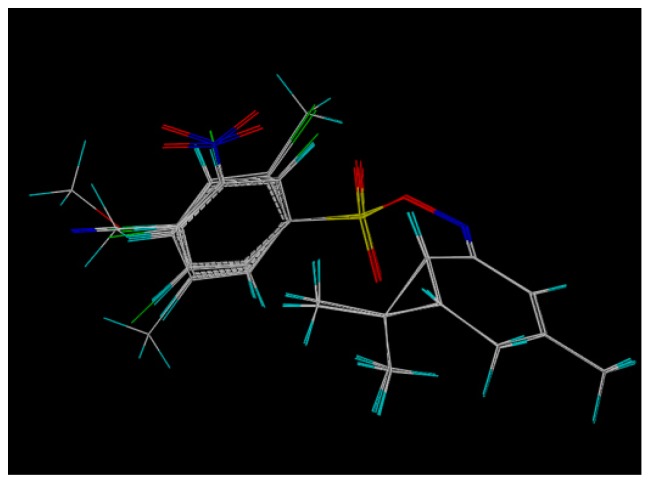
Superposition modes of compounds.

Complete conformational optimization of each structure was performed using a conjugate gradient procedure based on the Tripos force field and Gasteiger-Hückel charges. The compound (*Z*)-**4f** was used as a template to build the other molecular structures. According to the common skeleton marked with an asterisk showing in Figure 4, sixteen optimized molecules containing benzene ring were superimposed (Figure 5). The values of the CoMFA field were automatically calculated by the SYBYL/CoMFA routine. A predictive 3D-QSAR model was established using CoMFA descriptors as independent variables and ED values as dependent variables. The cross-validation with the leave-one-out mothed was carried out to obtain the cross-validated q^2^ and the optimal number of components. Then, A non-cross-validation analysis under the optimal number of components was performed. The modeling capability was indicated by the correlation coefficient squared r^2^, and the prediction capability was judged by the r^2^ and q^2^.

## 4. Conclusions

Using the natural product 3-carene as starting material, twenty-five novel 3-caren-5-one oxime sulfonate compounds were designed, synthesized, characterized, and evaluated for their antifungal activity. The bioassay result revealed that, compounds (*Z*)-**4b**, (*Z*)-**4f**, (*Z*)-**4m**, (*Z*)-**4e**, (*Z*)-**4j**, (*Z*)-**4l**, (*E*)-**4y**, (*Z*)-**4d**, and (*Z*)-**4p** exhibited excellent antifungal activity of 85.7%-100% against *P. piricola*, showing much better antifungal activity than that of the commercial fungicide chlorothanil. Both the compounds (*E*)-**4y** and (*E*)-**4x** displayed outstanding antifungal activity of 100% against *B. myadis*, and the former also displayed outstanding antifungal activity of 100% against *R. solani*. Furthermore, the target compound (*Z*)-**4b** also exhibited better antifungal activity against *C. arachidicola* than that of the positive control. Therefore, compounds (*Z*)-**4b**, (*Z*)-**4f**, (*Z*)-**4m**, (*E*)-**4y**, and (*E*)-**4x** are lead compounds worthy of further investigation. In order to design more effective antifungal compounds against *P. piricola*, the 3D-QSAR analysis was carried out using the CoMFA method. A reasonable and reliable 3D-QSAR model (r^2^ = 0.990, q^2^ = 0.569) has been established, and two new molecules with modification on phenyl gourps were proposed.

## Figures and Tables

**Table 1:** Antifungal activity of the target compounds (*Z*)-**4a**–**4u** and (*E*)-**4v**–**4y** at 50 µg/mL.

Compounds	Relative Inhibition Rate (%) against the Tested Fungi					
	*C. arachidicola*	*P. piricola*	*A. solani*	*R. solani*	*B. myadis*	*C. orbicalare*
(*Z*)-**4a** (R = *n*-ethyl)	74.2	35.7	34.4	29.2	5	17.5
(*Z*)-**4b** (R = *n*-butyl)	87.1	100	1.6	46.9	36.6	34
(*Z*)-**4c** (R = Ph)	9.7	7.1	27.9	23.3	20.8	13.4
(*Z*)-**4d** (R = *o*-Me Ph)	61.3	85.7	27.9	58.7	28.7	25.8
(*Z*)-**4e** (R = *p*-Me Ph)	48.4	92.9	27.9	42.4	36.6	17.5
(*Z*)-**4f** (R = 2,5-Me Ph)	0	100	8.2	48.3	32.7	21.6
(*Z*)-**4g** (R = *p*-F Ph)	22.6	0	0	35.1	24.8	29.9
(*Z*)-**4h** (R = 2,4-F Ph)	9.7	0	0	2.6	16.8	29.9
(*Z*)-**4i** (R = 2,3,4,5,6-F Ph)	74.2	0	8.2	0	12.9	13.4
(*Z*)-**4j** (R = *o*-Cl Ph)	9.7	92.9	21.3	43.9	16.8	21.6
(*Z*)-**4k** (R = *p*-Cl Ph)	0	0	1.6	23.3	20.8	25.8
(*Z*)-**4l** (R = 3,5-Cl Ph)	61.3	92.9	34.4	49.8	20.8	17.5
(*Z*)-**4m** (R = *o*-Br Ph)	0	100	27.9	23.3	20.8	25.8
(*Z*)-**4n** (R = *m*-Br Ph)	0	0	8.2	45.4	5	21.6
(*Z*)-**4o** (R = *p*-Br Ph)	48.4	14.3	8.2	20.3	12.9	29.9
(*Z*)-**4p** (R = *m*-NO_2_ Ph)	48.4	85.7	8.2	52.8	5	29.9
(*Z*)-**4q** (R = *p*-CN Ph)	22.6	0	14.8	17.3	12.9	13.4
(*Z*)-**4r** (R = *p*-MeO Ph)	9.7	0	27.9	29.2	5	17.5
(*Z*)-**4s** (R = *p*-Cl-m-NO_2_ Ph)	16.1	42.9	1.6	52.8	5	17.5
(*Z*)-**4t** (R = *γ*-biphenyl)	22.6	28.6	14.8	18.8	20.8	9.3
(*Z*)-**4u** (R = *α*-thienyl)	9.7	64.3	21.3	24.7	5	17.5
(*E*)-**4v** (R = Ph)	0	14.3	0	23.3	8.9	0
(*E*)-**4w** (R = *p*-Me Ph)	0	42.9	21.3	29.2	20.8	9.3
(*E*)-**4x** (R = *p*-Cl Ph)	9.7	57.1	8.2	89.7	100	1
(*E*)-**4y** (R = *p*-Br Ph)	0	92.9	27.9	100	100	9.3
Chlorothanil	73.3	75	73.9	96.1	90.4	91.3

**Table 2:** The ED values of experimental and predicted activities.

Compounds	ED	ED’’	Residue
(*Z*)-**4c** (R = Ph)	−3.60	−3.61	0.01
(*Z*)-**4d** (R = *o*-Me Ph)	−1.73	−1.88	0.15
(*Z*)-**4e** (R = *p*-Me Ph)	−1.39	−1.44	0.05
(*Z*)-**4f** (R = 2,5-Me Ph)	−0.53	−0.45	−0.08
(*Z*)-**4g** (R = *p*-F Ph)	−4.51	−4.61	0.10
(*Z*)-**4n** (R = 2,4-F Ph)	−4.53	−4.57	0.04
(*Z*)-**4j** (R = *o*-Cl Ph)	−1.41	−1.23	−0.18
(*Z*)-**4k** (R = *p*-Cl Ph)	−4.53	−4.06	−0.47
(*Z*)-**4l** (R = 3,5-Cl Ph)	−1.45	−1.50	0.05
(*Z*)-**4m** (R = *o*-Br Ph)	−0.59	−0.70	0.11
(*Z*)-**4n** (R = *m*-Br Ph)	−4.58	−4.62	0.04
(*Z*)-**4o** (R = *p*-Br Ph)	−3.36	−3.56	0.20
(*Z*)-**4p** (R = *m*-NO_2_ Ph)	−1.77	−1.71	−0.06
(*Z*)-**4q** (R = *p*-CN Ph)	−4.51	−4.53	0.02
(*Z*)-**4r** (R = *p*-MeO Ph)	−4.52	−4.53	0.01
(*Z*)-**4s** (R = *p*-Cl-*m*-NO_2_ Ph)	−2.71	−2.73	0.02

**Table 3:** Summary of CoMFA Aanalysis.

					Contribution (%)	
	q^2^	r^2^	S	F	Steric	Electrostatic
CoMFA	0.569	0.990	0.243	68.914	95.0	5.0

q^2^: cross-validated correlational coefficient, r^2^: non-validated correlational coefficient, S: standard error of estimate, F: the Fischer ratio.

## Supplementary Materials

Supplementary materials are available online.
