# Supplementary material for: Synthesis of Bioactive Compounds from 3-Carene (II): Synthesis, Antifungal Activity and 3D-QSAR Study of (Z)- and (E)-3-Caren-5-One Oxime Sulfonates
Source: Molecules. 2019 Jan 29;24(3):477. doi: 10.3390/molecules24030477 (PMC6384770; doi:10.3390/molecules24030477)
Supplement: Supplementary file 1 [file molecules-24-00477-s001.pdf]

Supplementary materials: Synthesis of Bioactive Compounds from 3-Carene (Ⅱ): Synthesis, Antifungal Activity and 3D-QSAR Study of (*Z*)- and (*E*)-3-Caren-5-One Oxime Sulfonates

Guo-Qiang Kang, Wen-Gui Duan*, Gui-Shan Lin*, You-Pei Yu, Xiao-Yu Wang and Sun-Zhong Lu

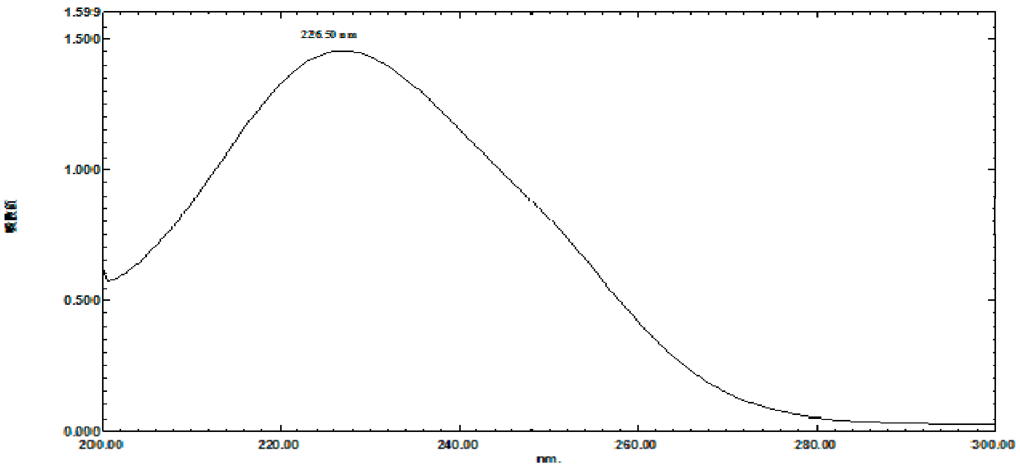

Figure S1. UV-vis spectrum of 3-carene-5-one (**2**) in EtOH.

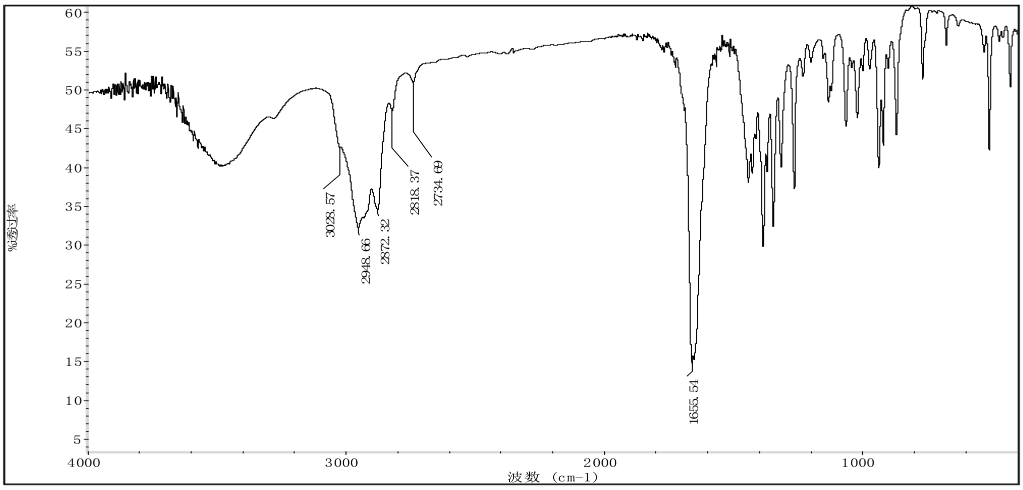

Figure S2. FTIR spectrum of 3-carene-5-one (**2**).

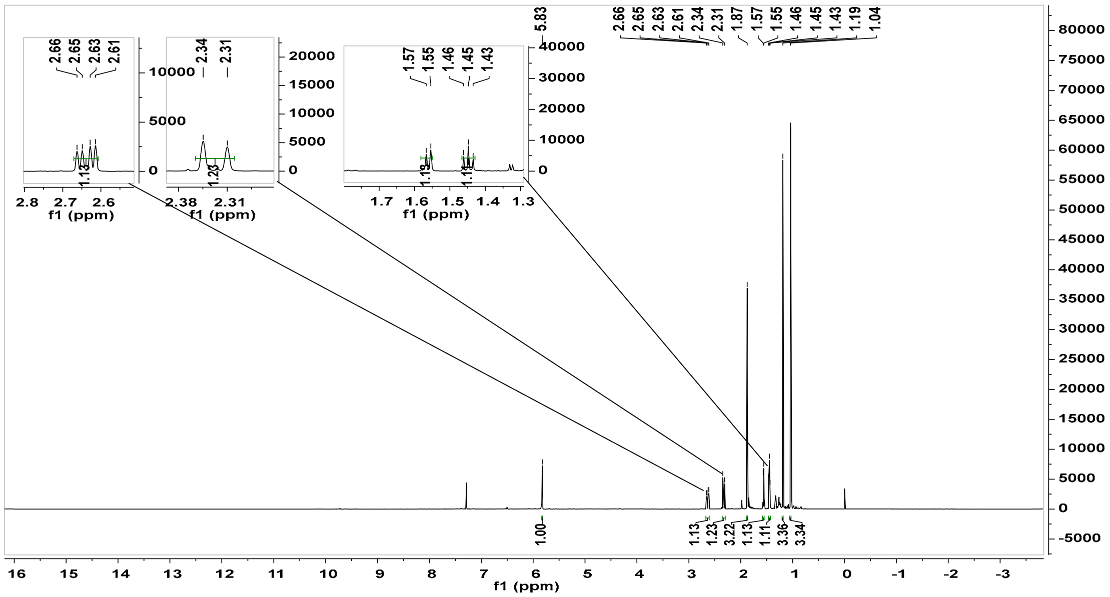

Figure S3. ^{1}H-NMR spectrum of 3-carene-5-one (**2**) in $CDCl_3$.

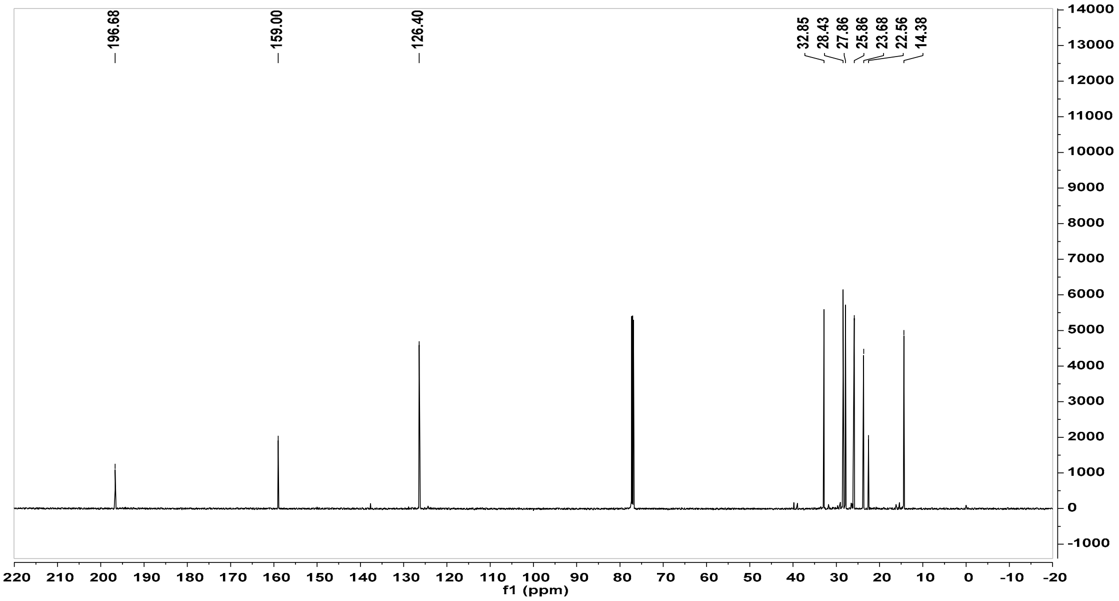

Figure S4. ^{13}C-NMR spectrum of 3-carene-5-one (**2**) in $CDCl_3$.

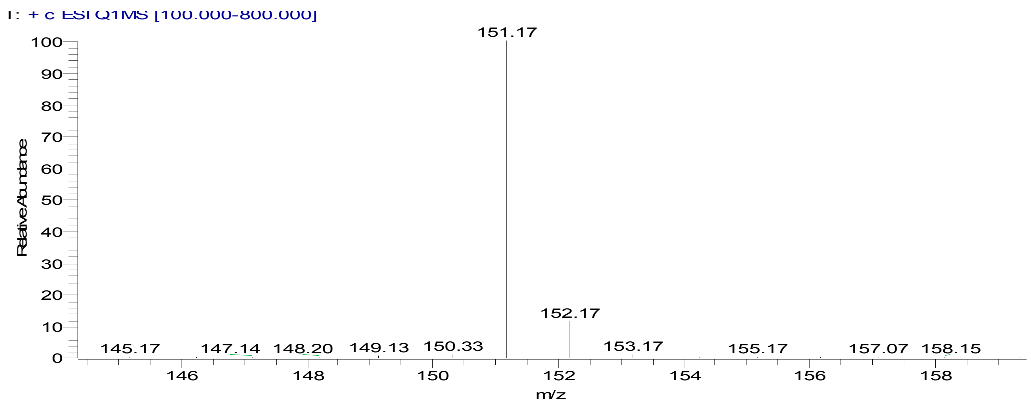

Figure S5. ESI-MS spectrum of 3-carene-5-one (**2**).

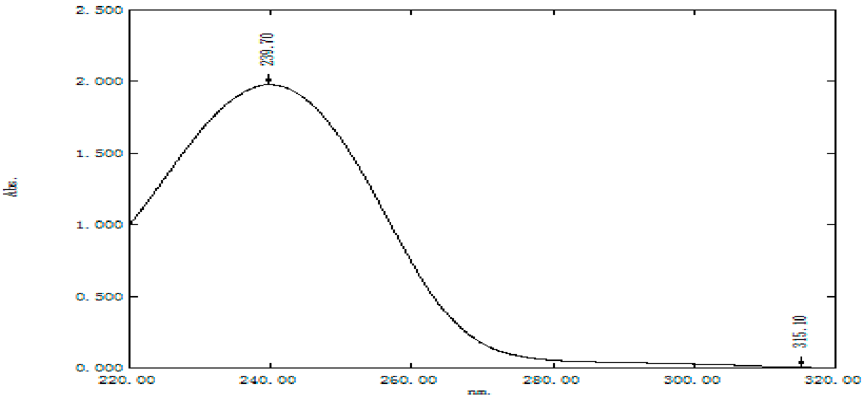

Figure S6. UV-vis spectrum of (*Z*)-3-carene-5-one oxime (**3a**) in EtOH.

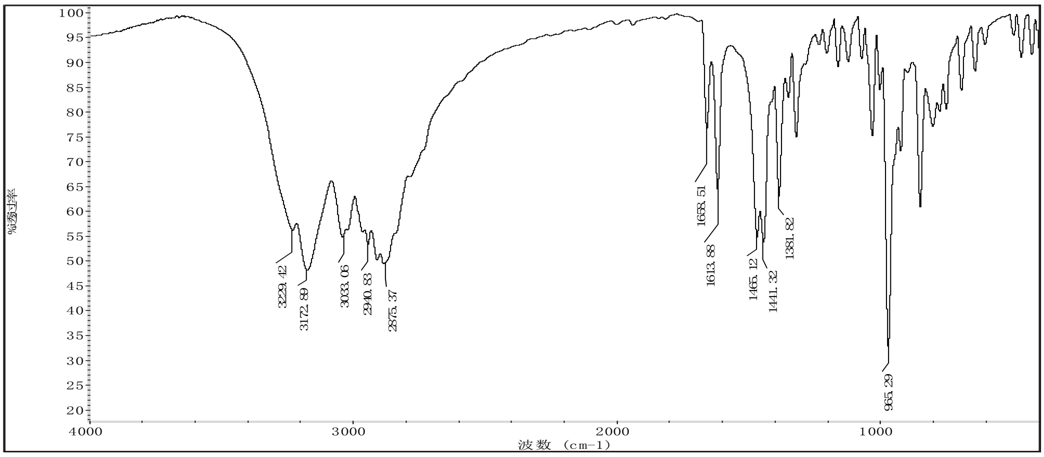

Figure S7. FTIR spectrum of (*Z*)-3-carene-5-one oxime (**3a**).

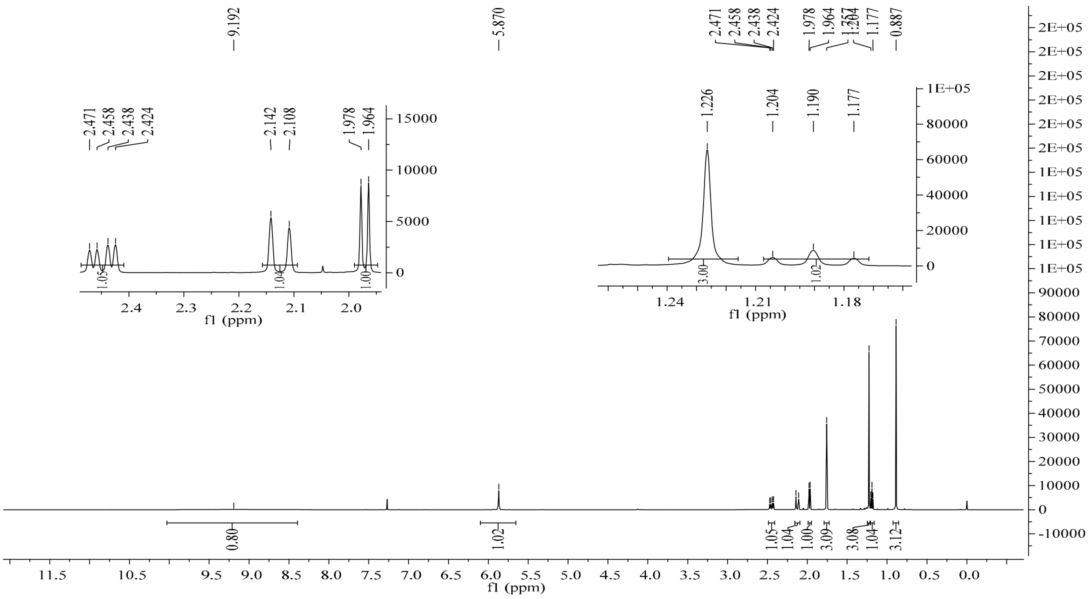

Figure S8. ^{1}H-NMR spectrum of (*Z*)-3-carene-5-one oxime (**3a**) in $CDCl_3$.

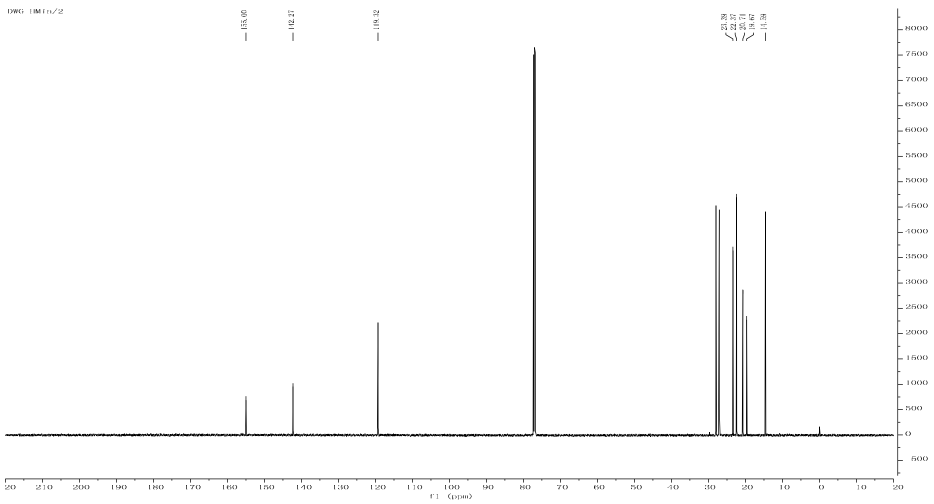

Figure S9. ^{13}C-NMR spectrum of (*Z*)-3-carene-5-one oxime (**3a**) in $CDCl_3$.

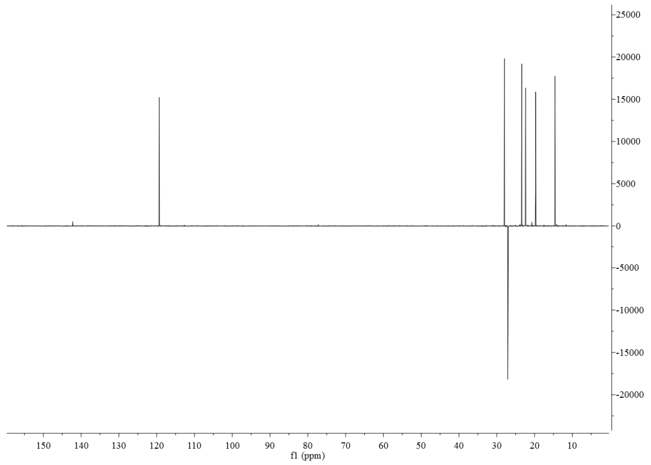

Figure S10. DEPT 135^0 spectrum of (*Z*)-3-carene-5-one oxime (**3a**) in $CDCl_3$.

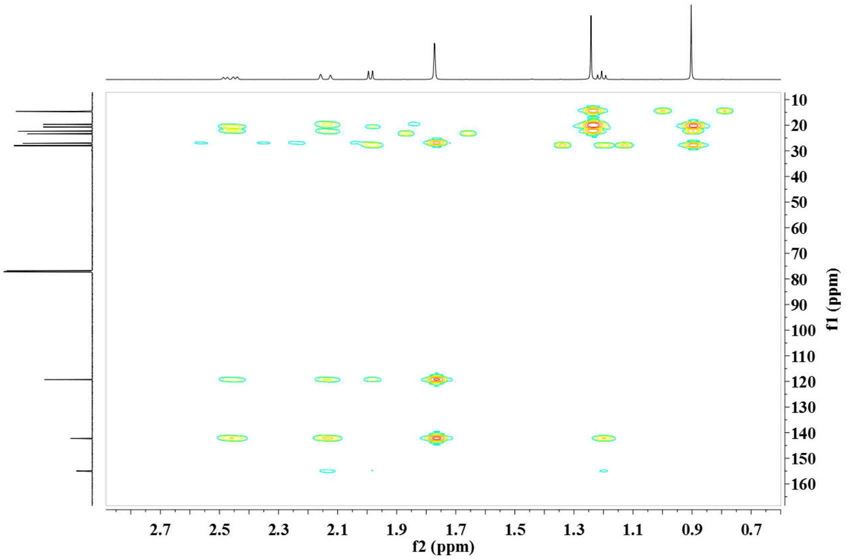

Figure S11. HMBC spectrum of (*Z*)-3-carene-5-one oxime (**3a**).

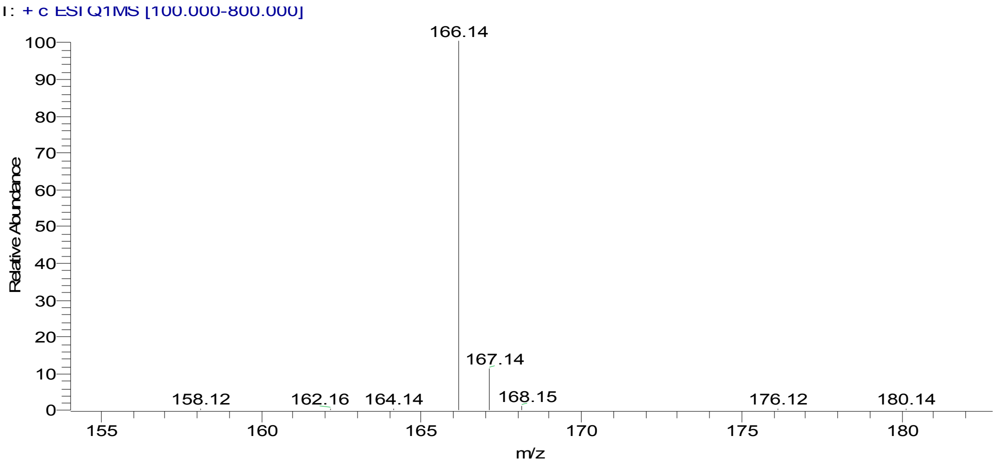

Figure S12. ESI-MS spectrum of (*Z*)-3-carene-5-one oxime (**3a).**

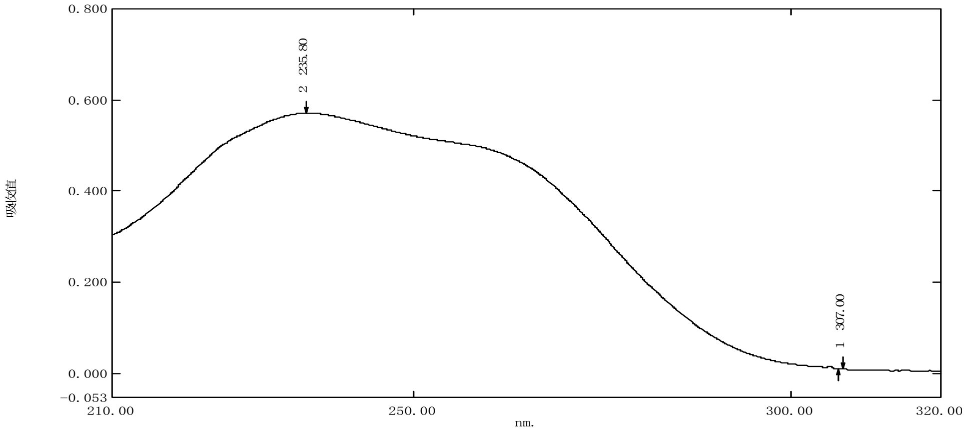

Figure S13. UV-vis spectrum of (*E*)-3-carene-5-one oxime (**3b**) in EtOH.

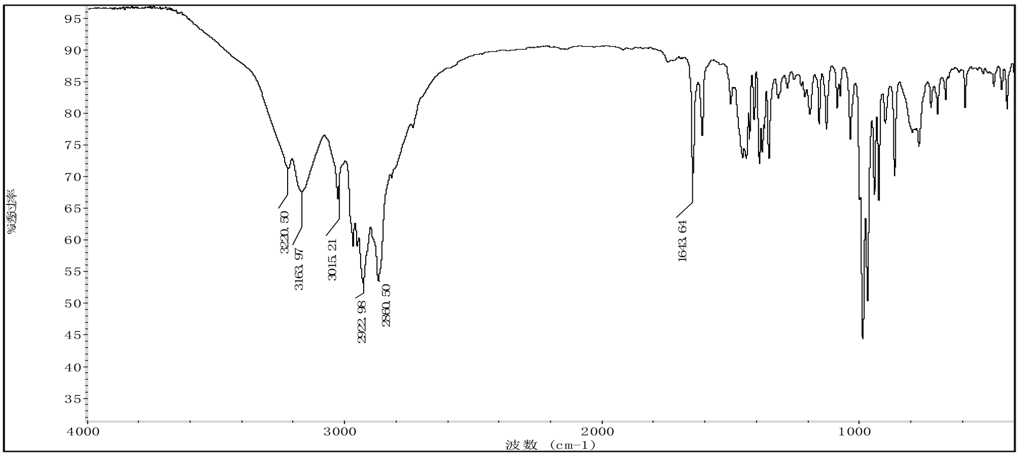

Figure S14. FTIR spectrum of (*E*)-3-carene-5-one oxime (**3b**).

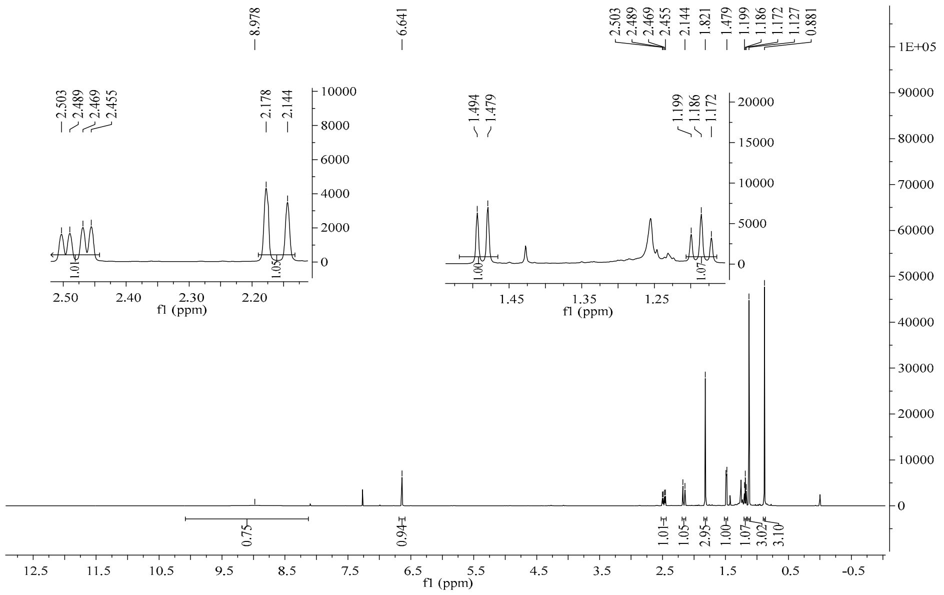

Figure S15. ^{1}H-NMR spectrum of (*E*)-3-carene-5-one oxime (**3b**) in $CDCl_3$.

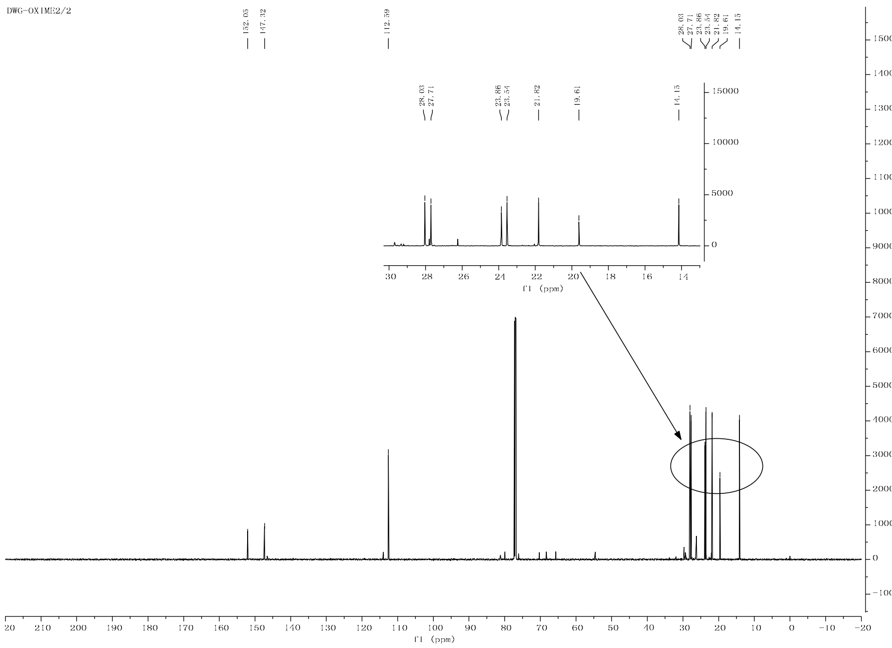

Figure S16. ^{13}C-NMR spectrum of (*E*)-3-carene-5-one oxime (**3b**) in $CDCl_3$

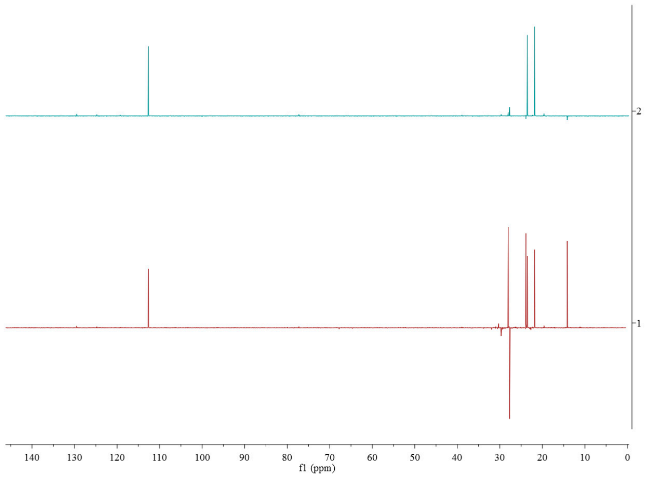

Figure S17. DEPT 90⁰ and 135⁰ spectrum of (*Z*)-3-carene-5-one oxime (**3b**) in $CDCl_3$.

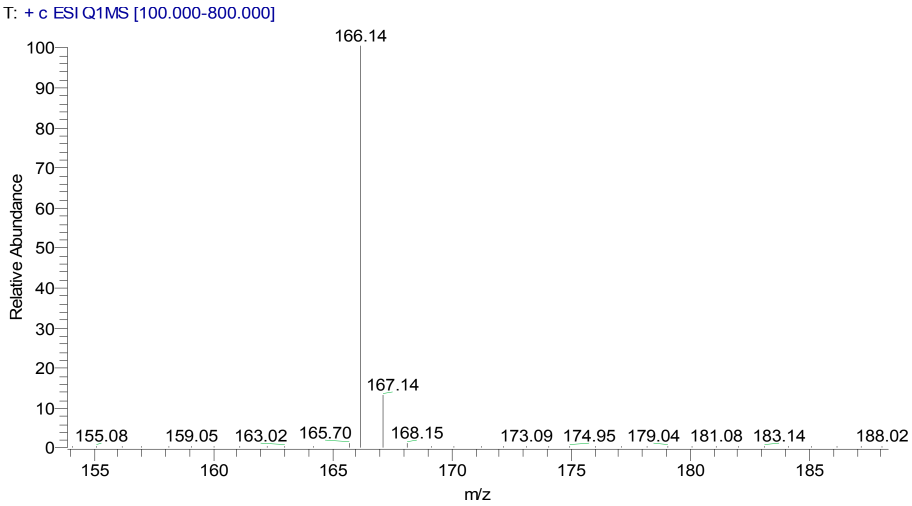

Figure S18. ESI-MS spectrum of (*E*)-3-carene-5-one oxime (**3b**).

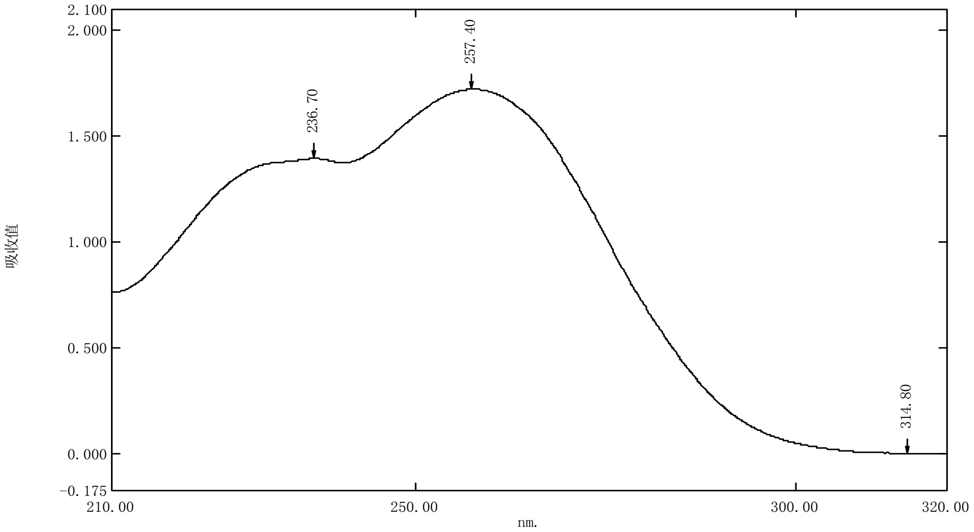

Figure S19. UV-vis spectrum of compound (**4a**) in EtOH.

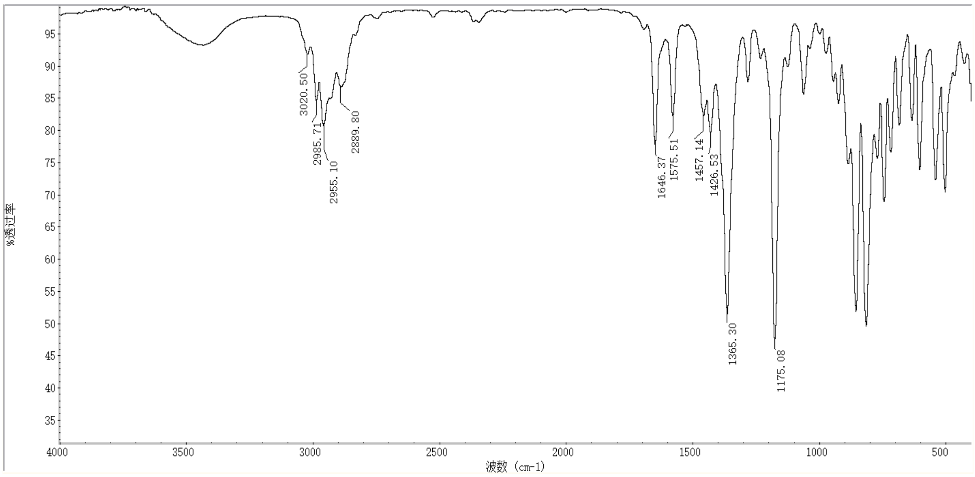

Figure S20. FTIR spectrum of compound (**4a**).

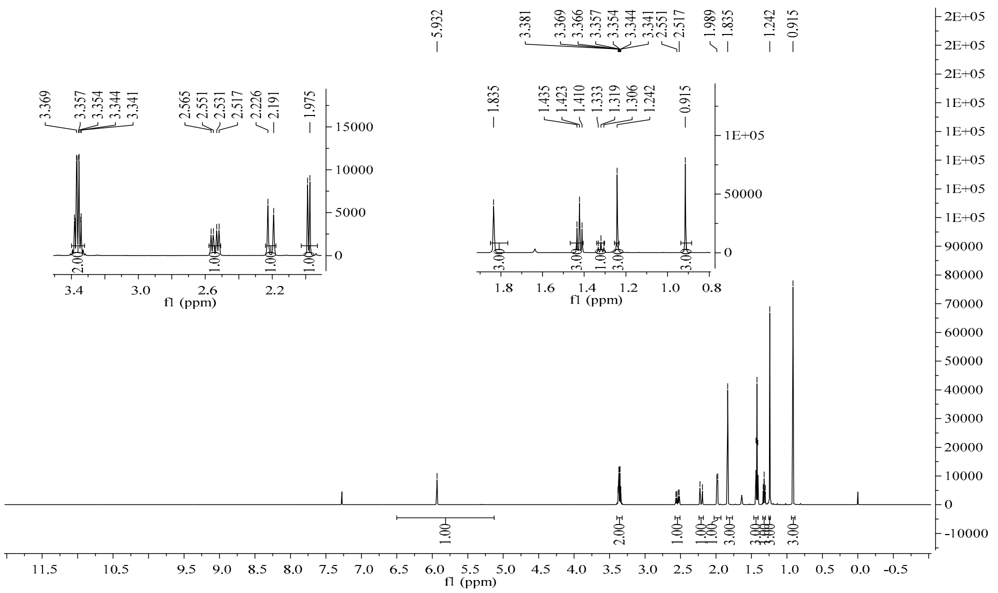

Figure S21. ^{1}H-NMR spectrum of compound (**4a**) in $CDCl_3$.

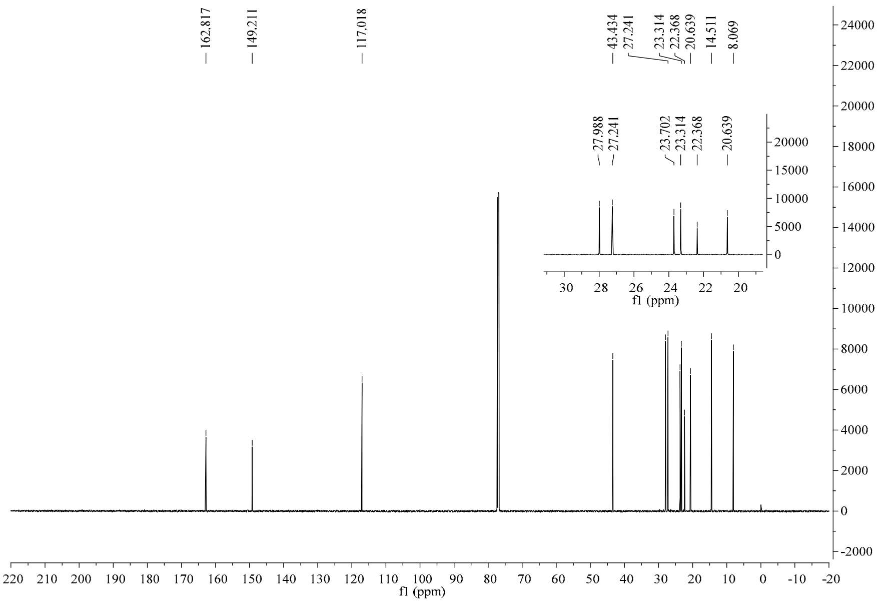

Figure S22. ¹³C-NMR spectrum of compound (**4a**) in $CDCl_3$.

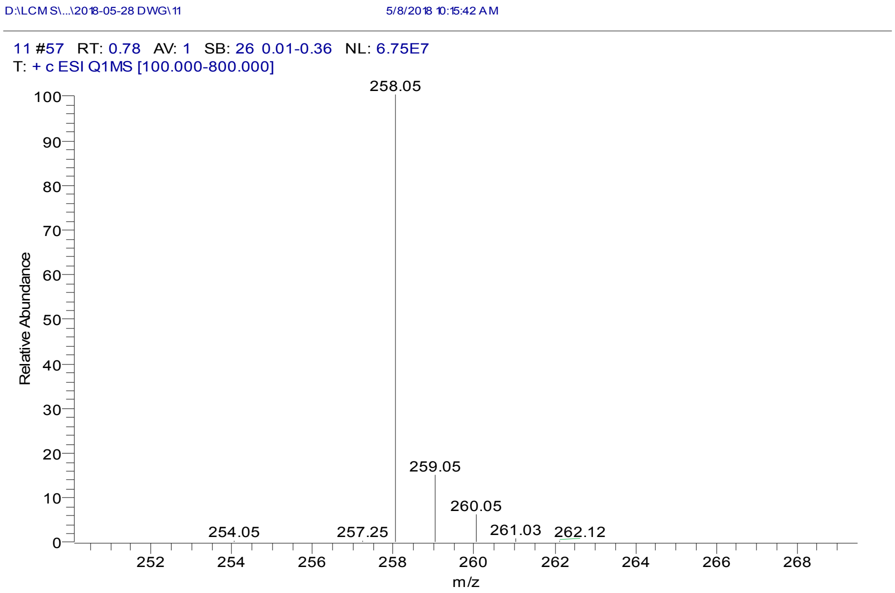

Figure S23. ESI-MS spectrum of compound (**4a).**

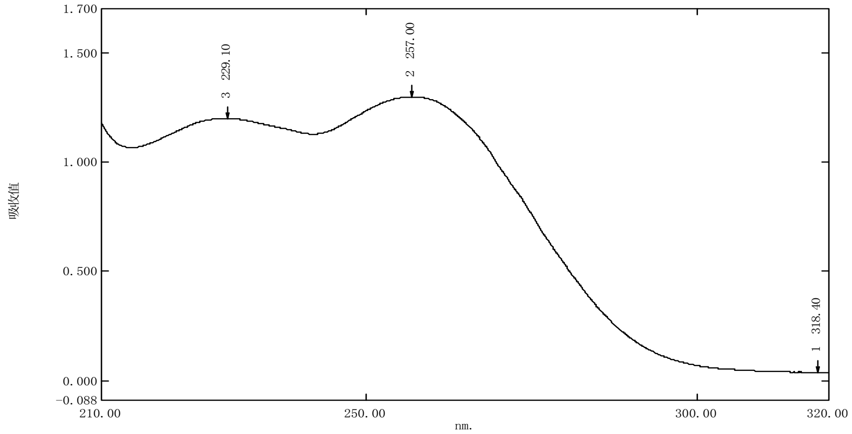

Figure S24. UV-vis spectrum of compound (**4b**) in EtOH.

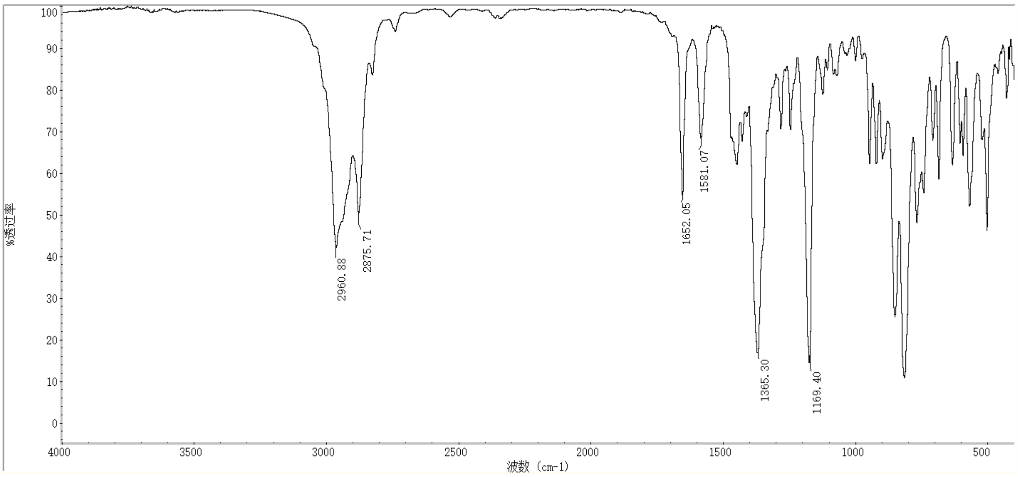

Figure S25. FTIR spectrum of compound (**4b**).

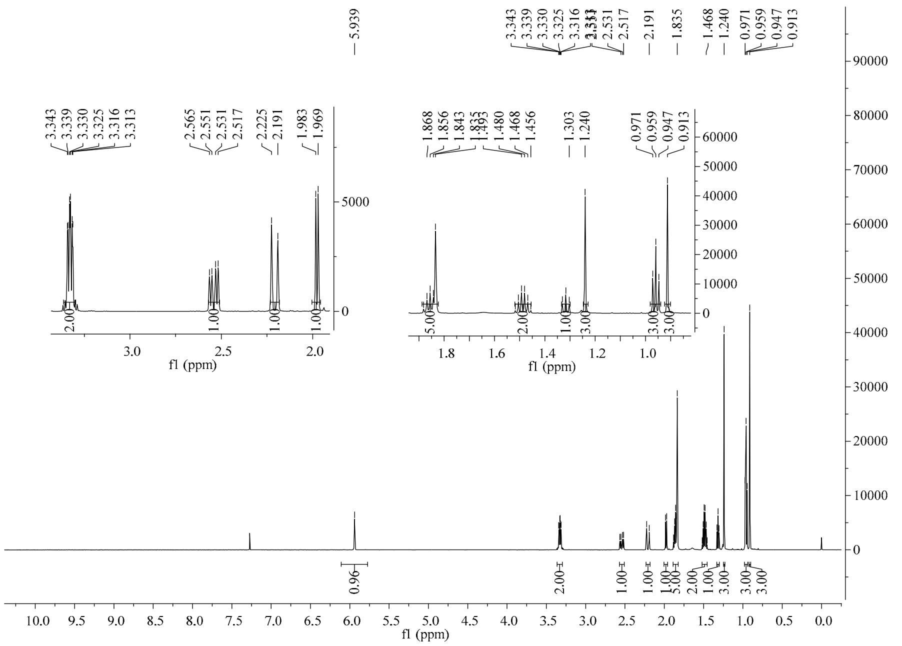

Figure S26. ^{1}H-NMR spectrum of compound (**4b**) in $CDCl_3$.

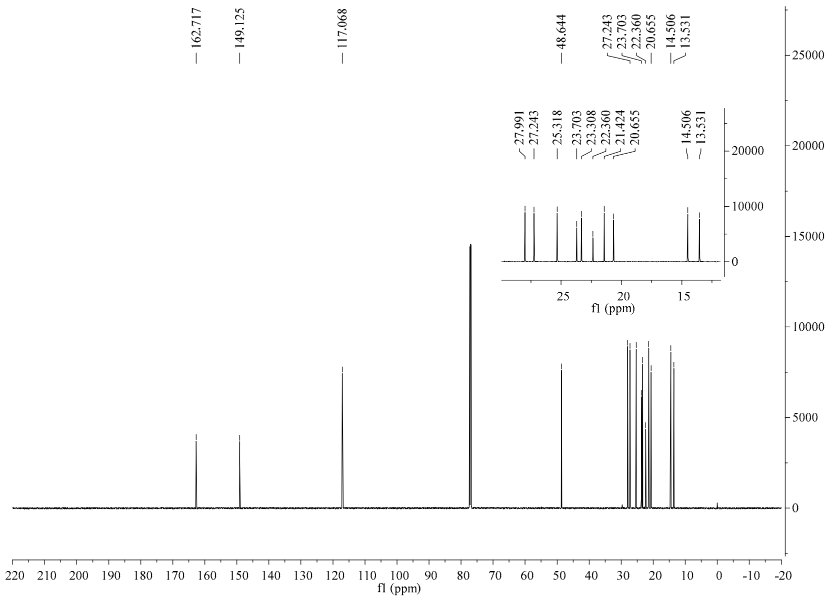

Figure S27. ^{13}C-NMR spectrum of compound (**4b**) in $CDCl_3$.

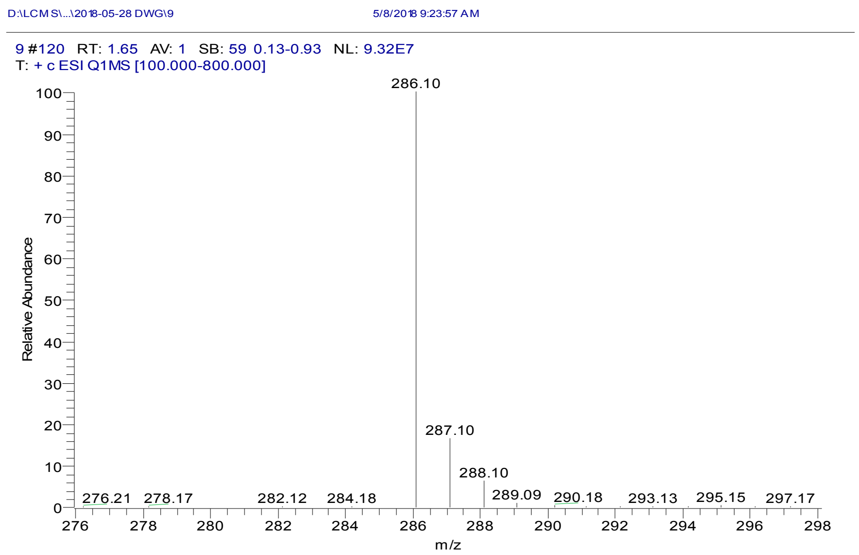

Figure S28. ESI-MS spectrum of compound (**4b).**

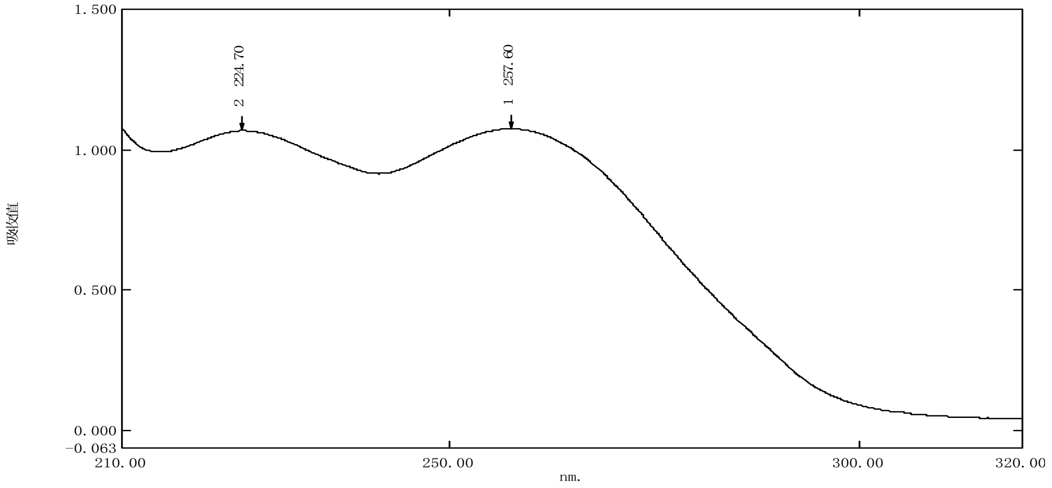

Figure S29. UV-vis spectrum of compound (**4c**) in EtOH.

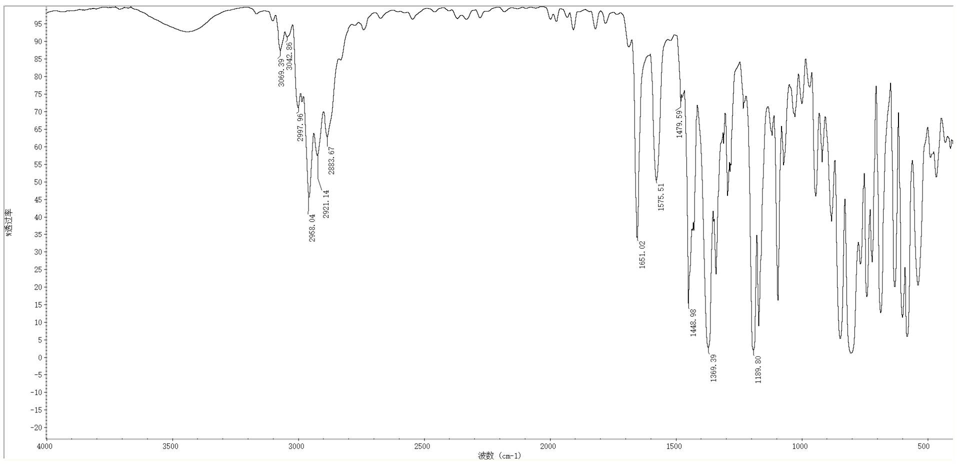

Figure S30. FTIR spectrum of compound (**4c**).

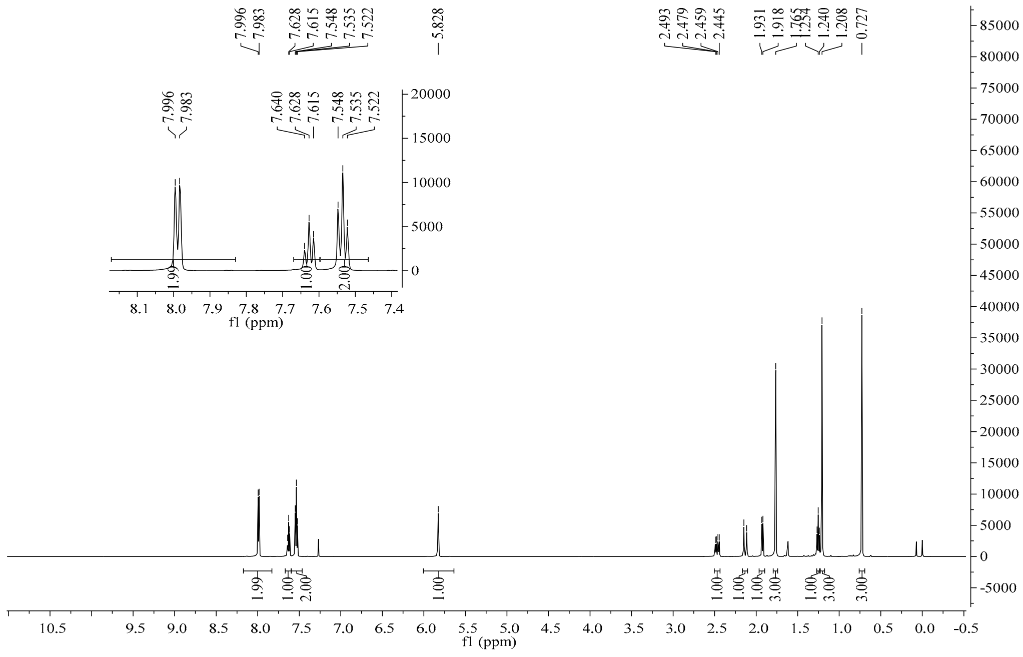

Figure S31. ^{1}H-NMR spectrum of compound (**4c**) in $CDCl_3$.

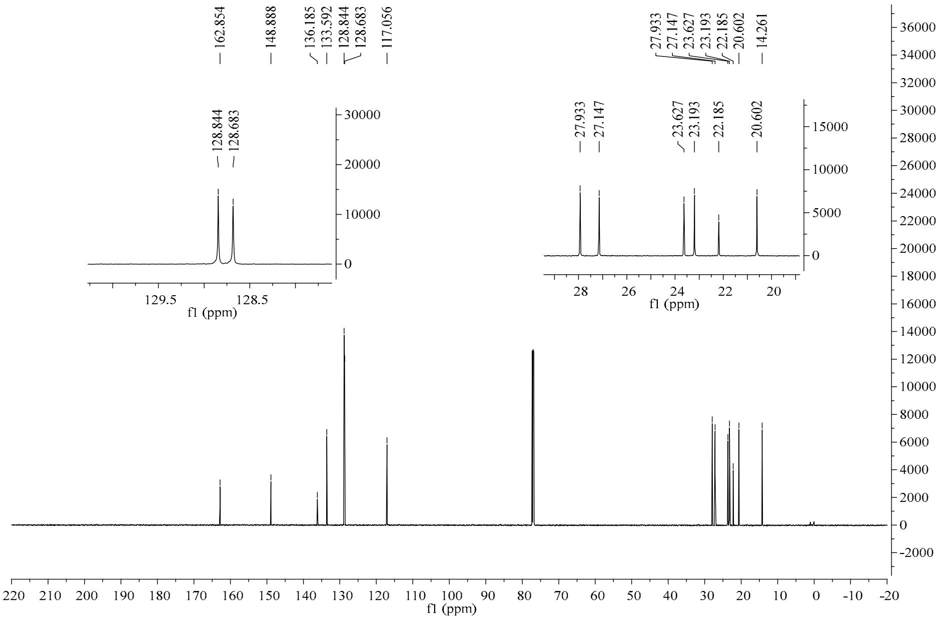

Figure S32. ^{13}C-NMR spectrum of compound (**4c**) in $CDCl_3$.

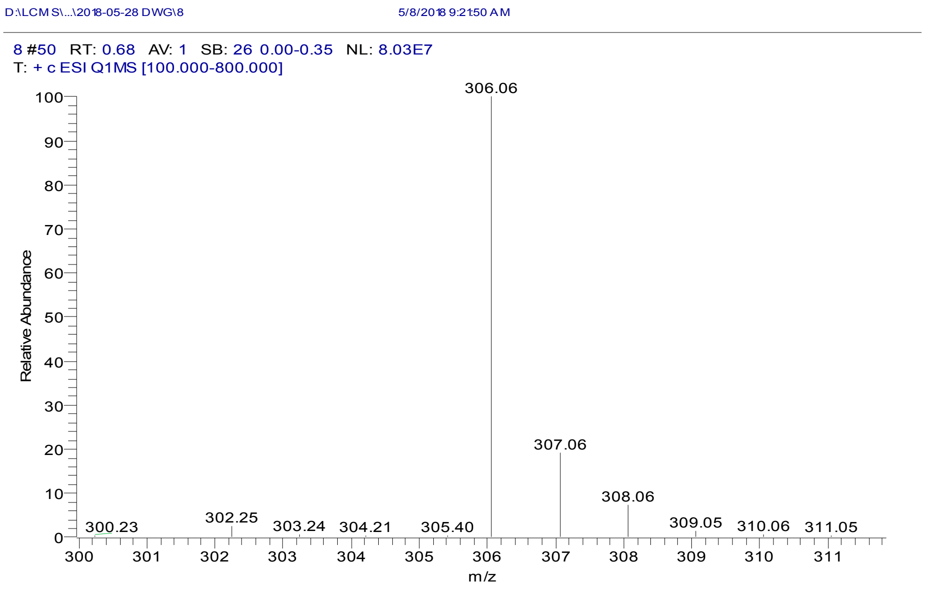

Figure S33. ESI-MS spectrum of compound (**4c).**

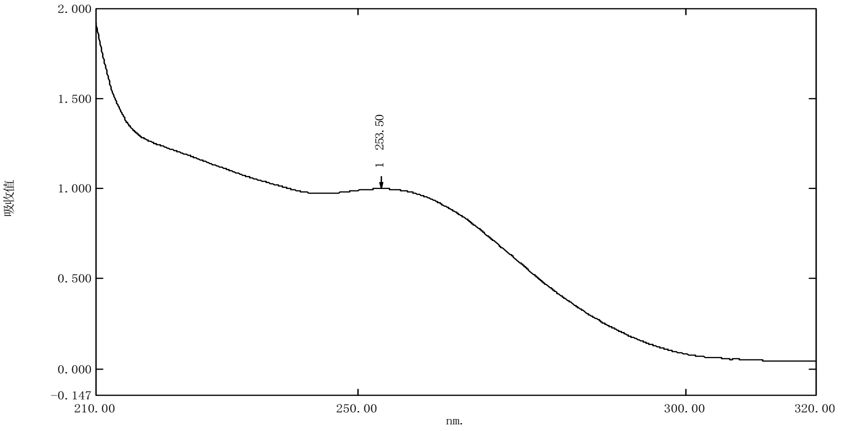

Figure S34. UV-vis spectrum of compound (**4d**) in EtOH.

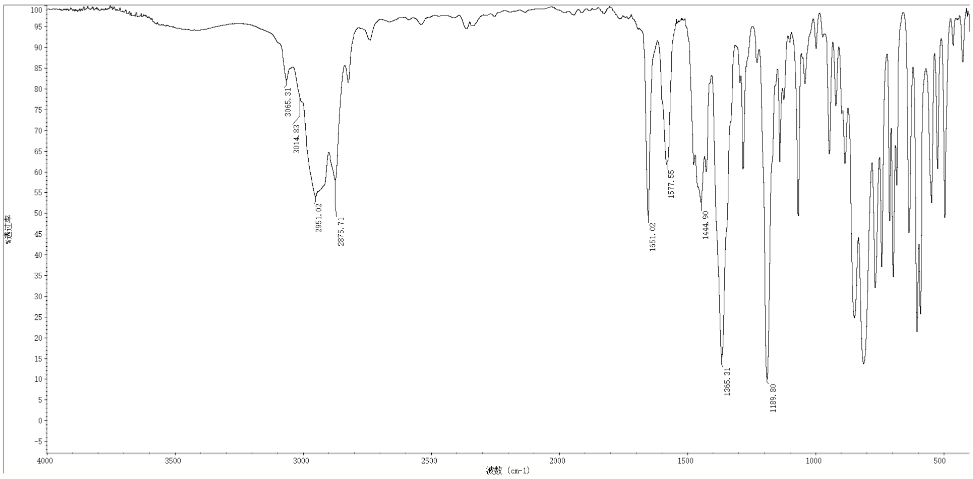

Figure S35. FTIR spectrum of compound (**4d**).

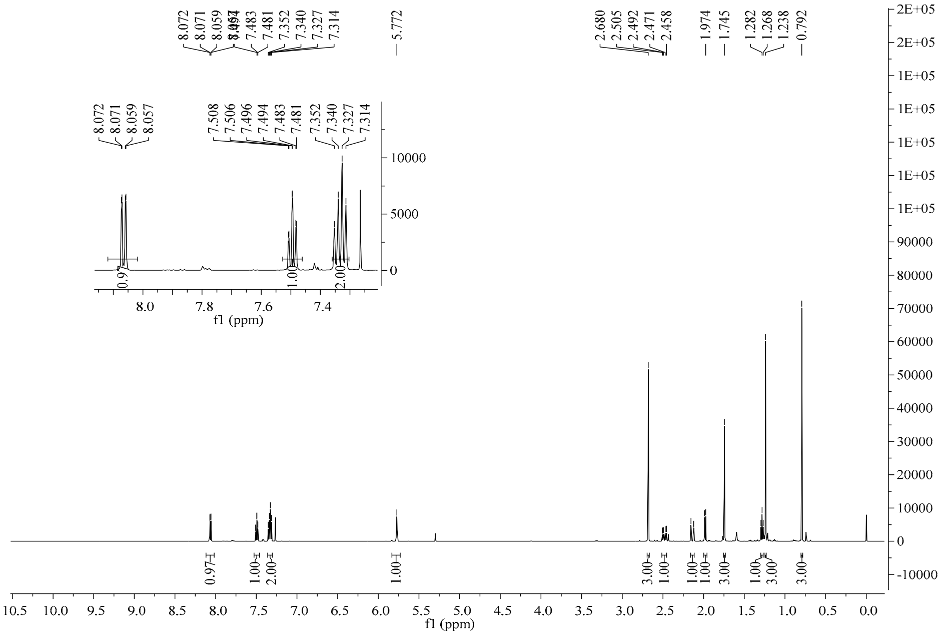

Figure S36. ^{1}H-NMR spectrum of compound (**4d**) in $CDCl_3$.

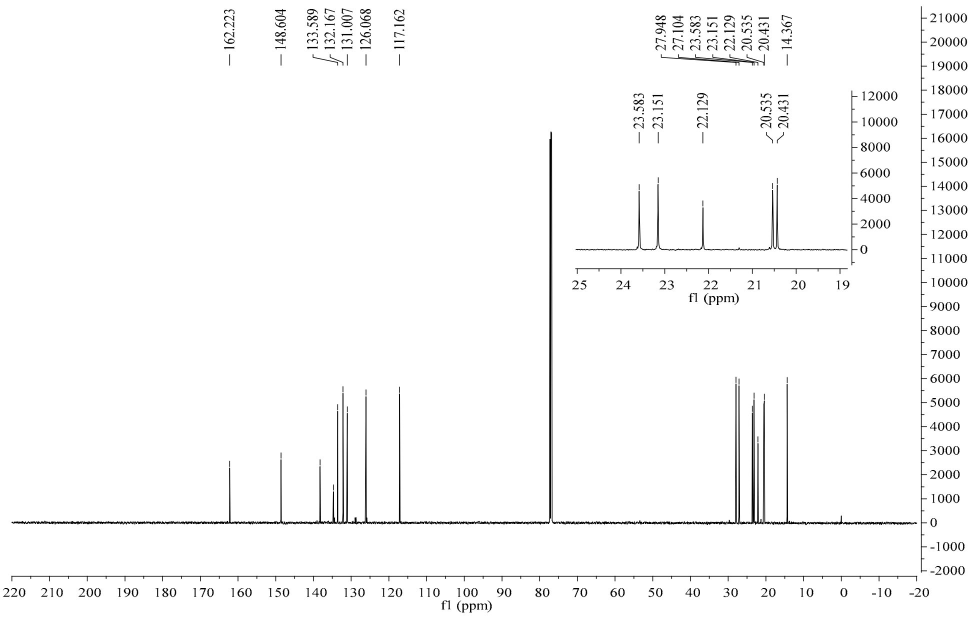

Figure S58. ^{13}C-NMR spectrum of compound (**4d** in $CDCl_3$.

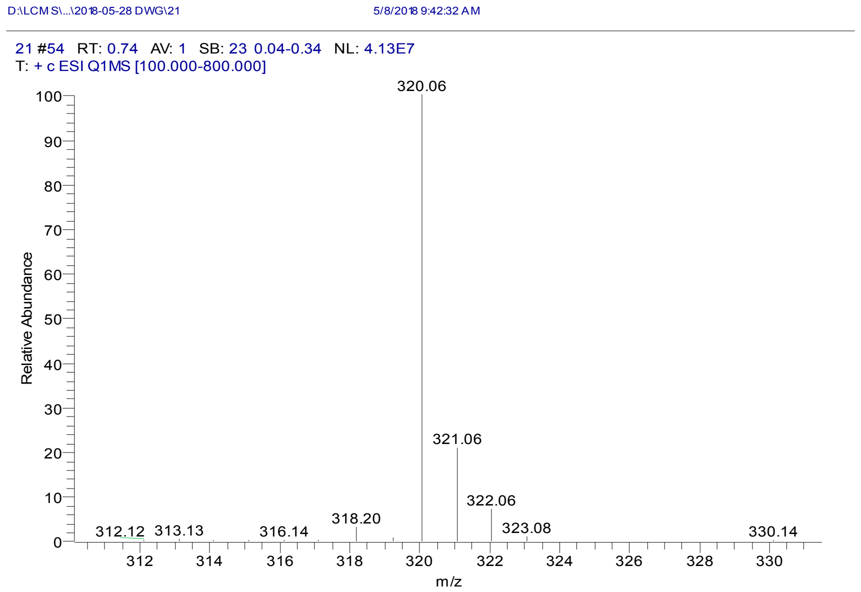

Figure S38. ESI-MS spectrum of compound (**4d).**

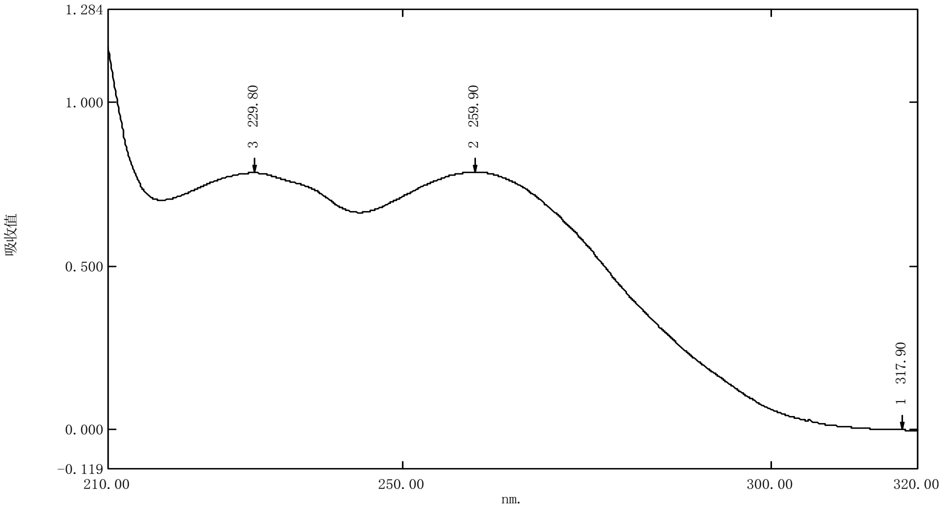

Figure S39. UV-vis spectrum of compound (**4e**) in EtOH.

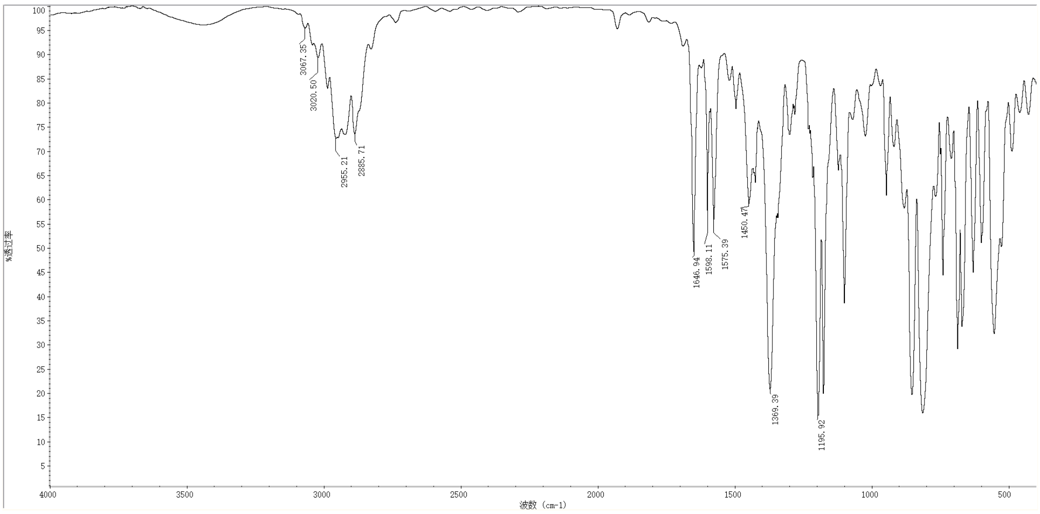

Figure S40. FTIR spectrum of compound (**4e**).

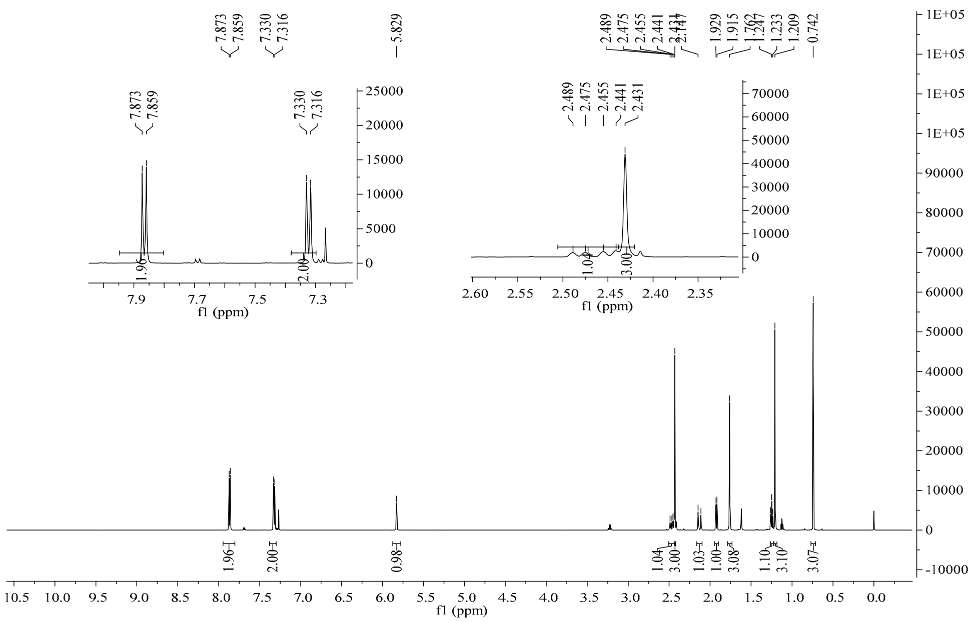

Figure S41. ^{1}H-NMR spectrum of compound (**4e**) in $CDCl_3$.

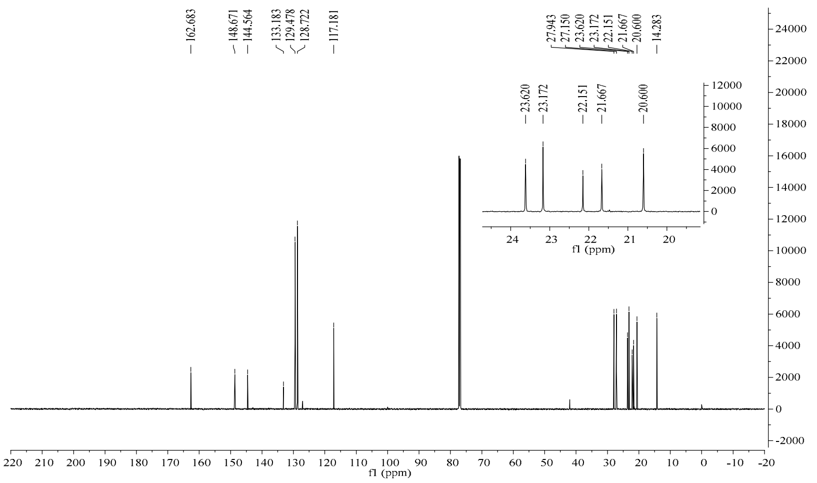

Figure S42. ^{13}C-NMR spectrum of compound (**4e**) in $CDCl_3$.

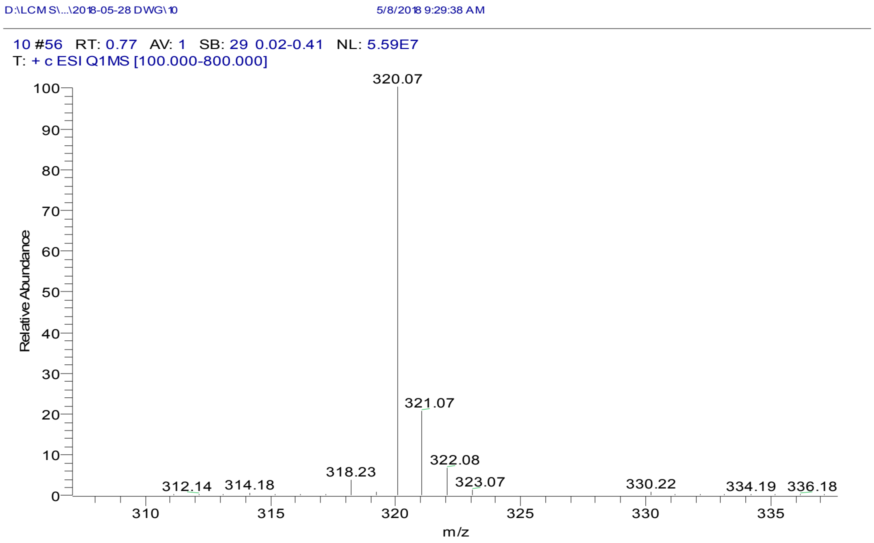

Figure S43. ESI-MS spectrum of compound (**4e).**

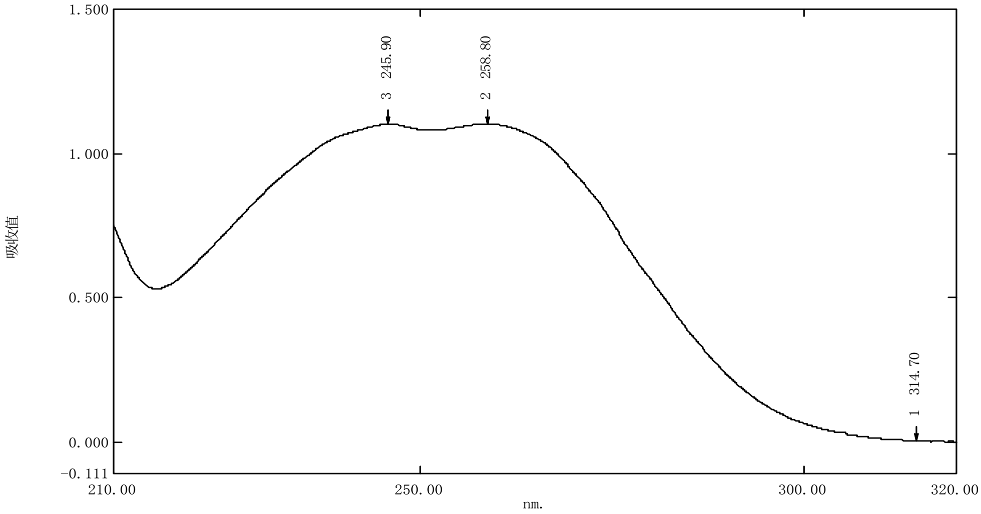

Figure S44. UV-vis spectrum of compound (**4f**) in EtOH.

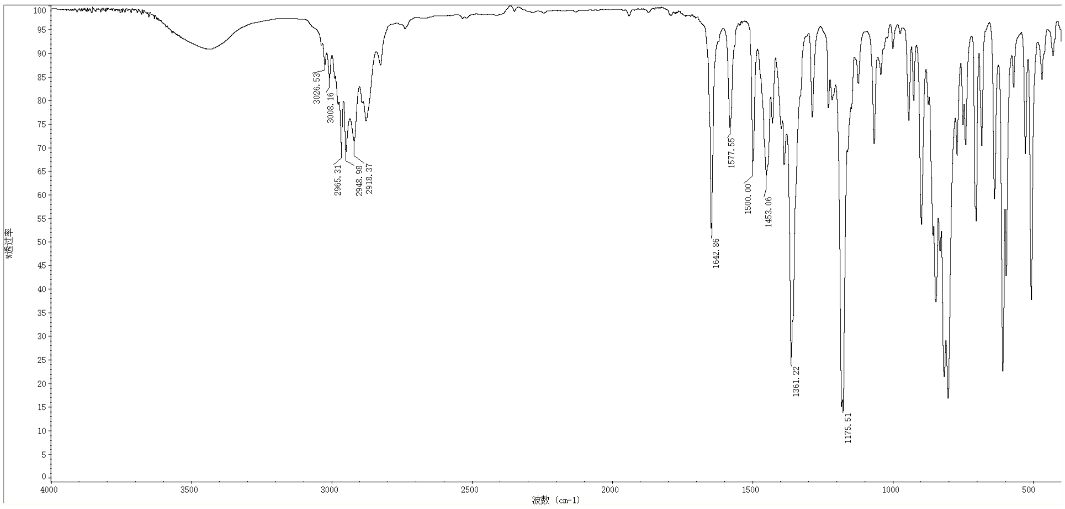

Figure S45. FTIR spectrum of compound (**4f**).

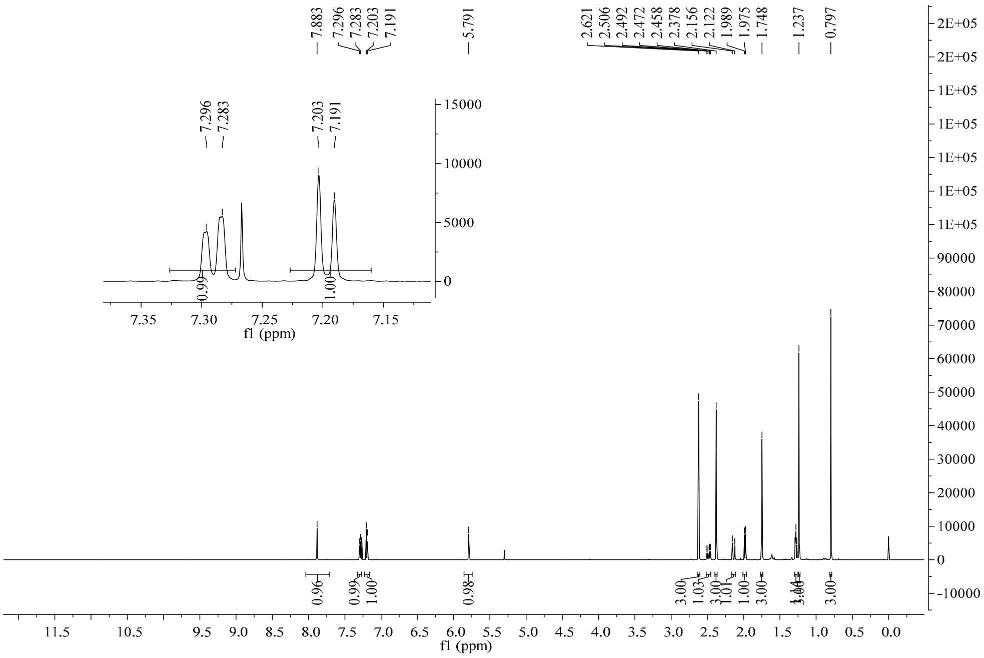

Figure S46. ^{1}H-NMR spectrum of compound (**4f**) in $CDCl_3$.

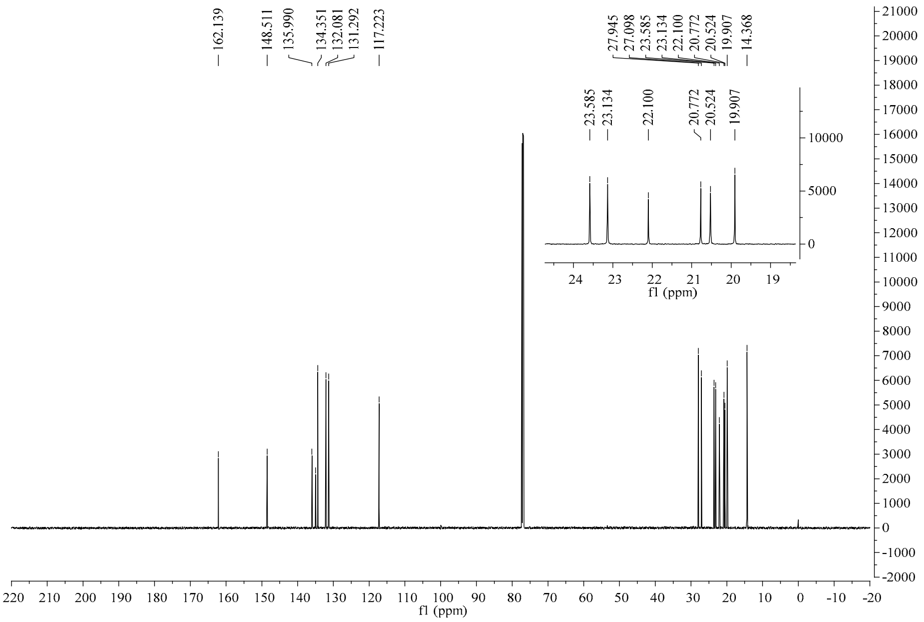

Figure S47. ^{13}C-NMR spectrum of compound (**4f**) in $CDCl_3$.

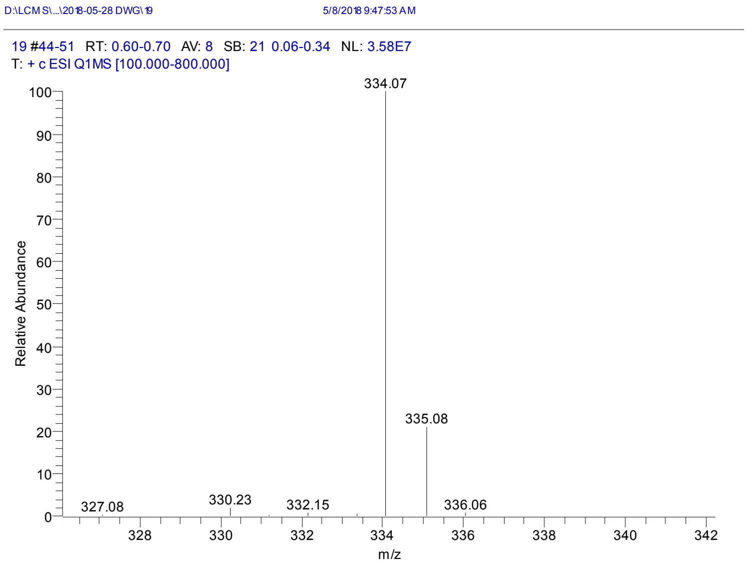

Figure S48. ESI-MS spectrum of compound (**4f).**

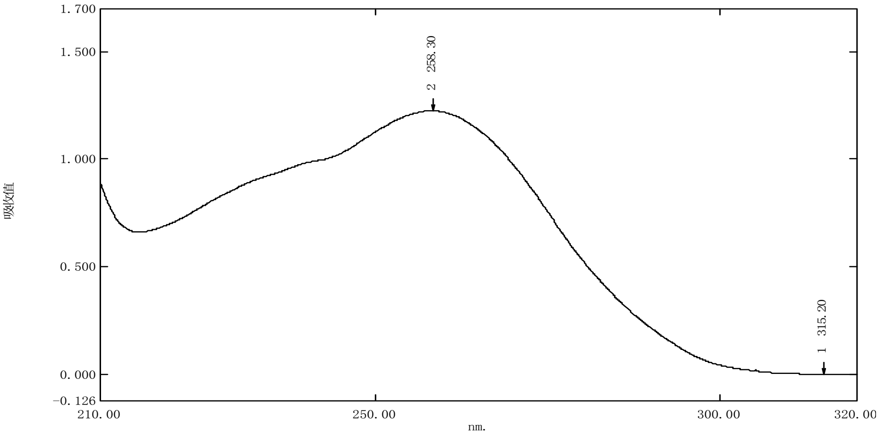

Figure S49. UV-vis spectrum of compound (**4g**) in EtOH.

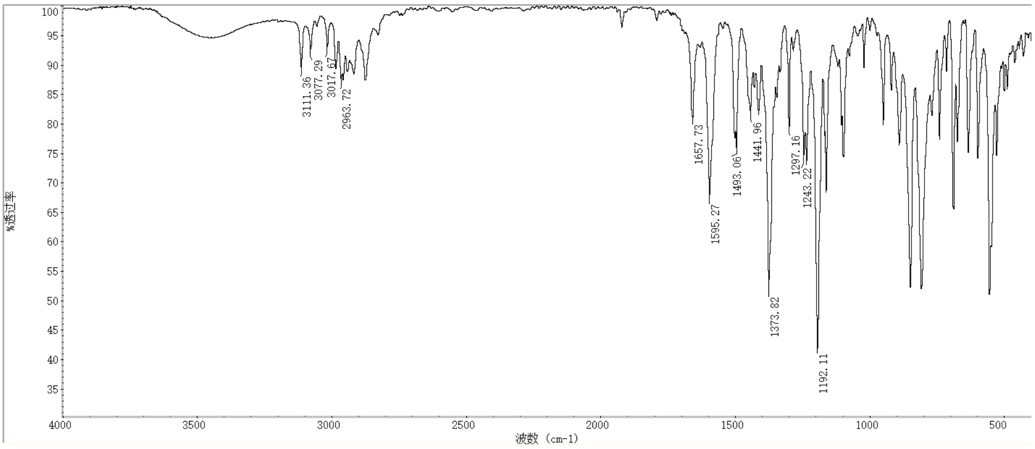

Figure S50. FTIR spectrum of compound (**4g**).

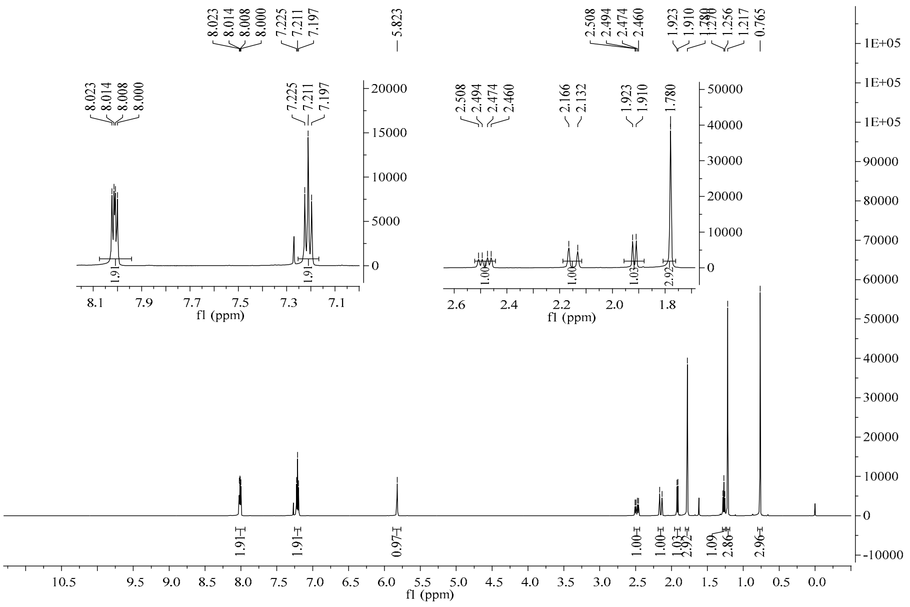

Figure S51. ^{1}H-NMR spectrum of compound (**4g**) in $CDCl_3$.

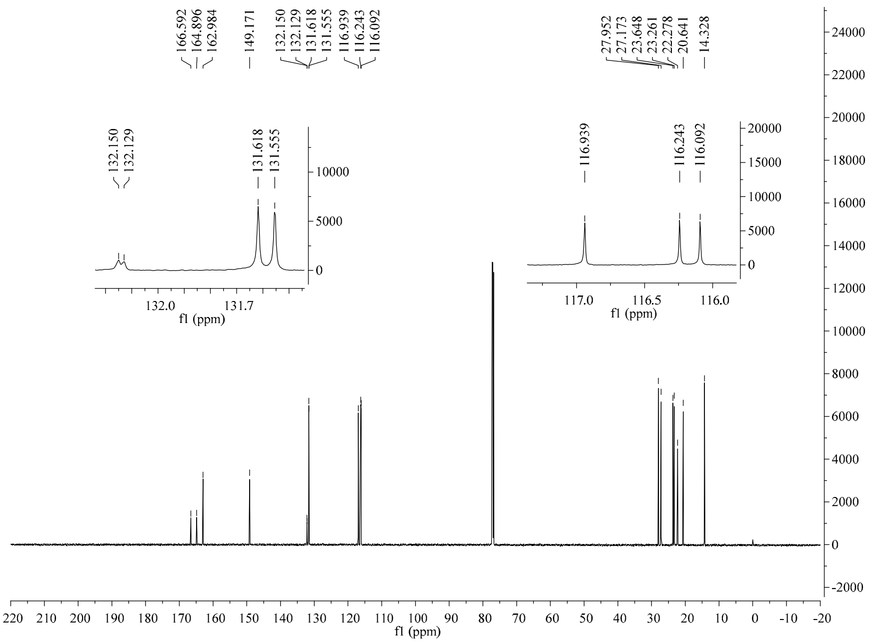

Figure S52. ^{13}C-NMR spectrum of compound (**4g**) in $CDCl_3$.

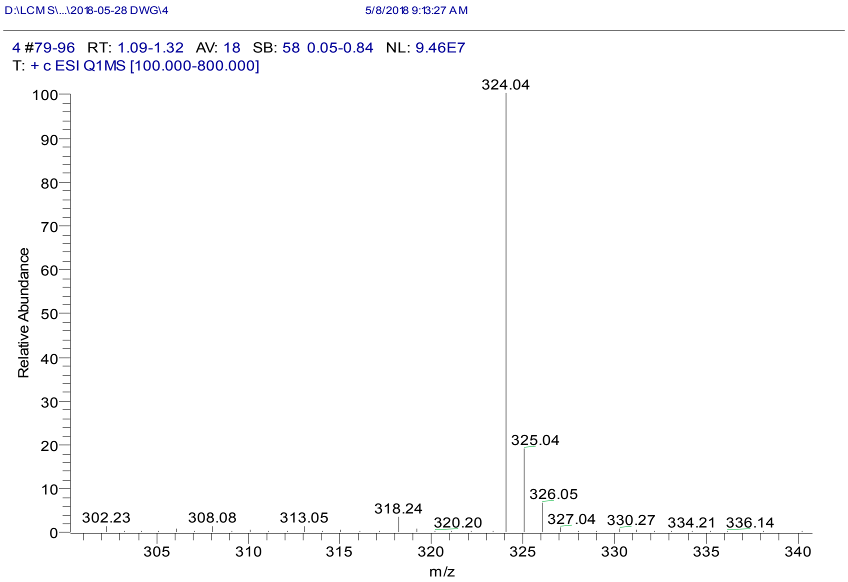

Figure S53. ESI-MS spectrum of compound (**4g**).

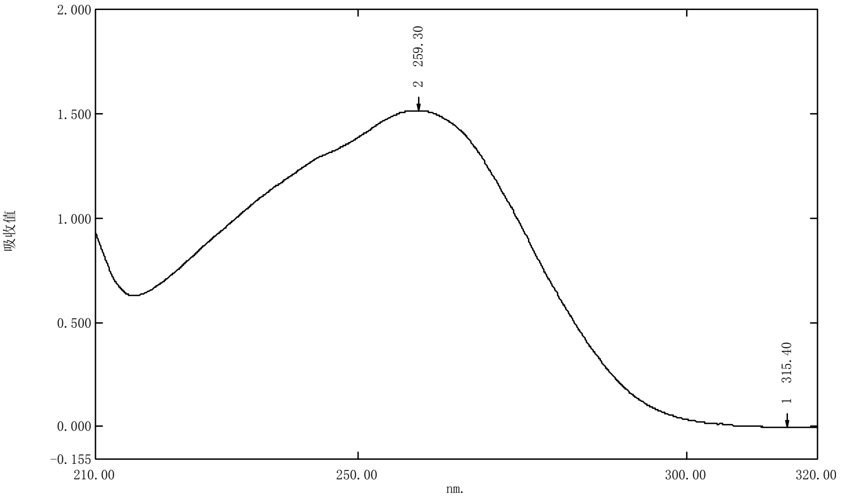

Figure S54. UV-vis spectrum of compound (**4h**) in EtOH.

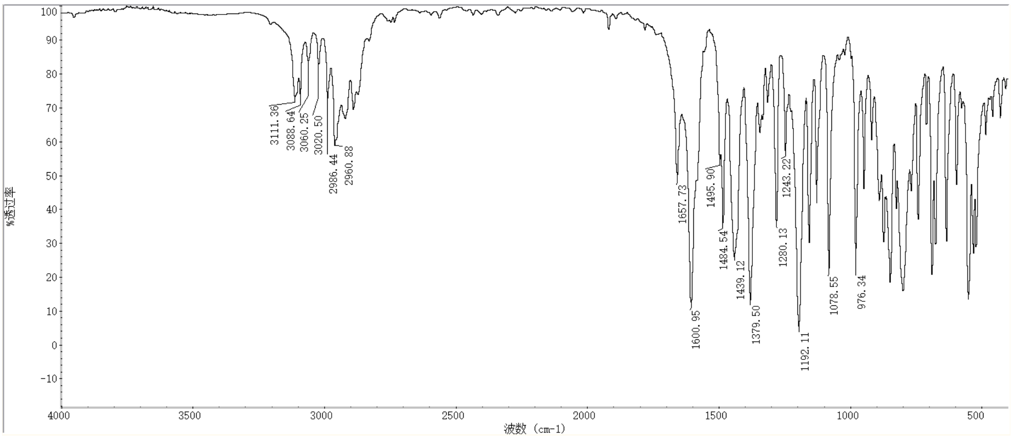

Figure S55. FTIR spectrum of compound (**4h**).

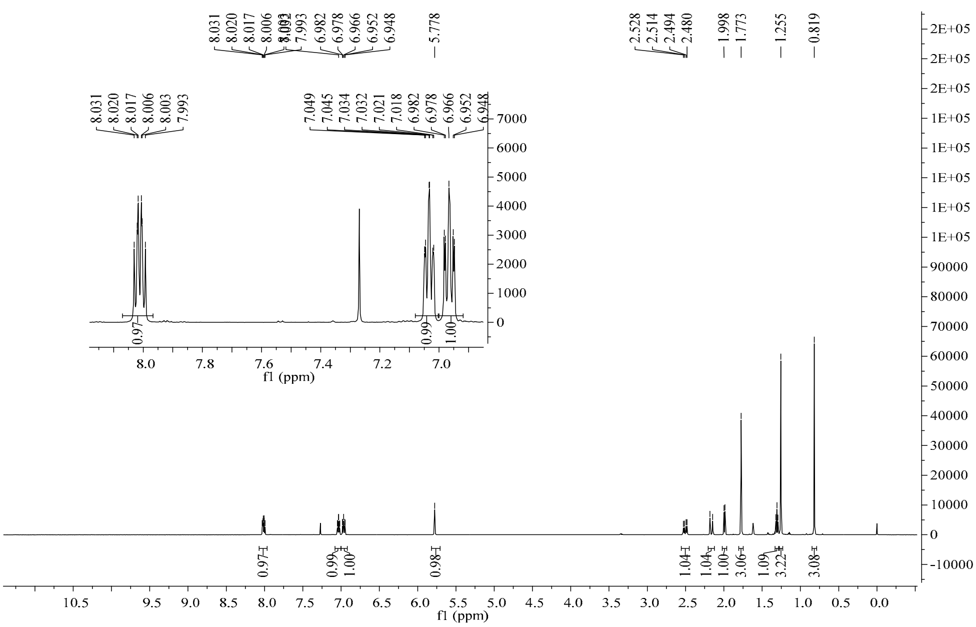

Figure S56. ^{1}H-NMR spectrum of compound (**4h**) in $CDCl_3$.

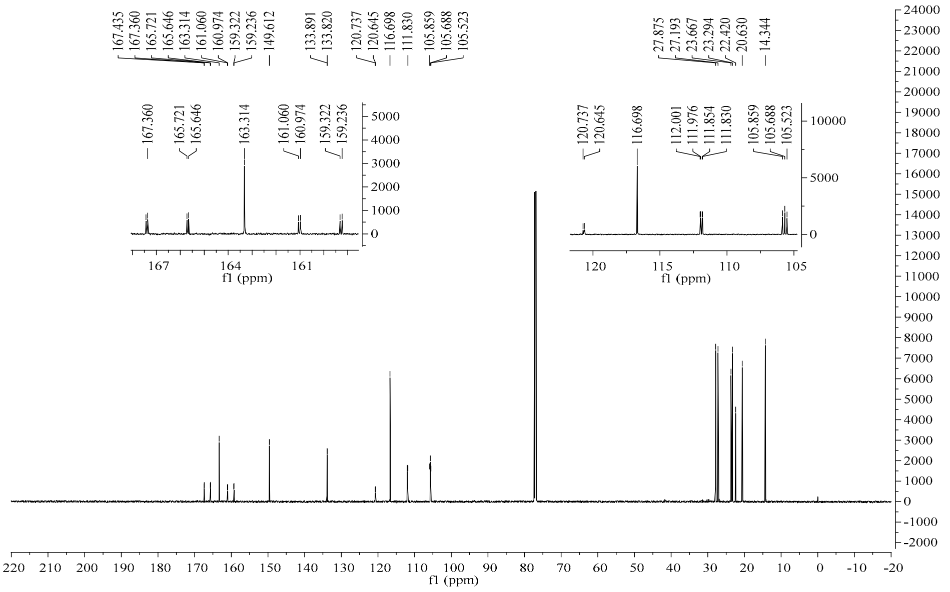

Figure S57. ^{13}C-NMR spectrum of compound (**4h**) in $CDCl_3$.

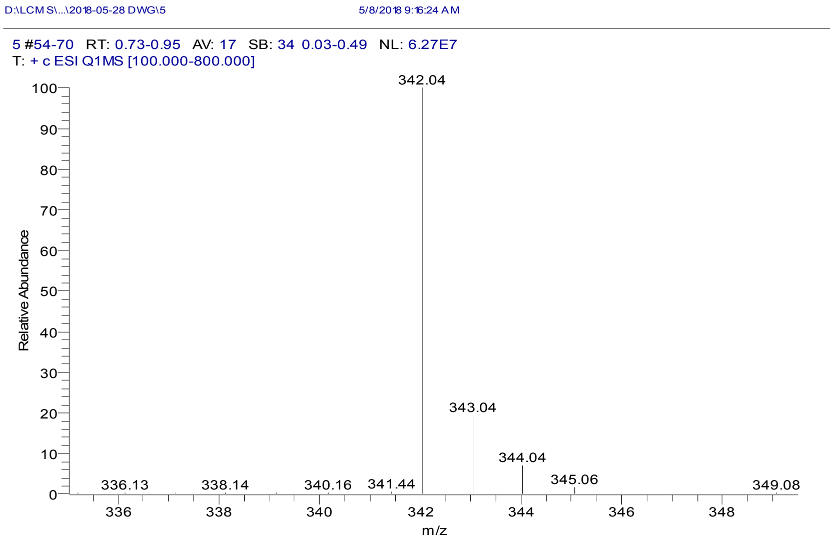

Figure S58. ESI-MS spectrum of compound (**4h)**

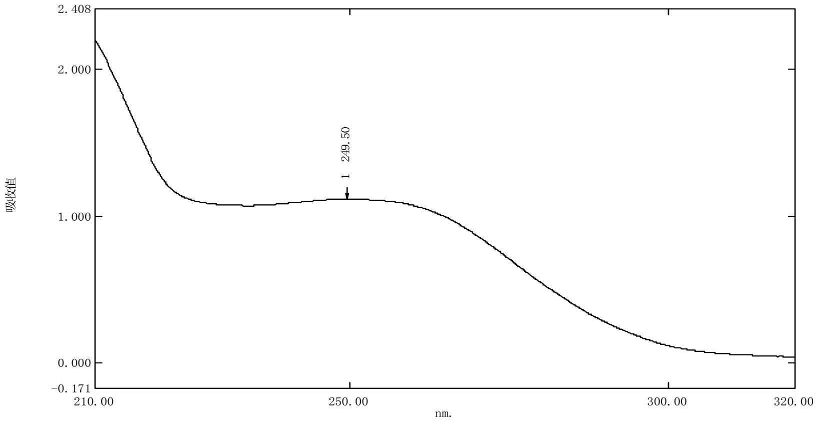

Figure S59 UV-vis spectrum of compound (**4i**) in EtOH.

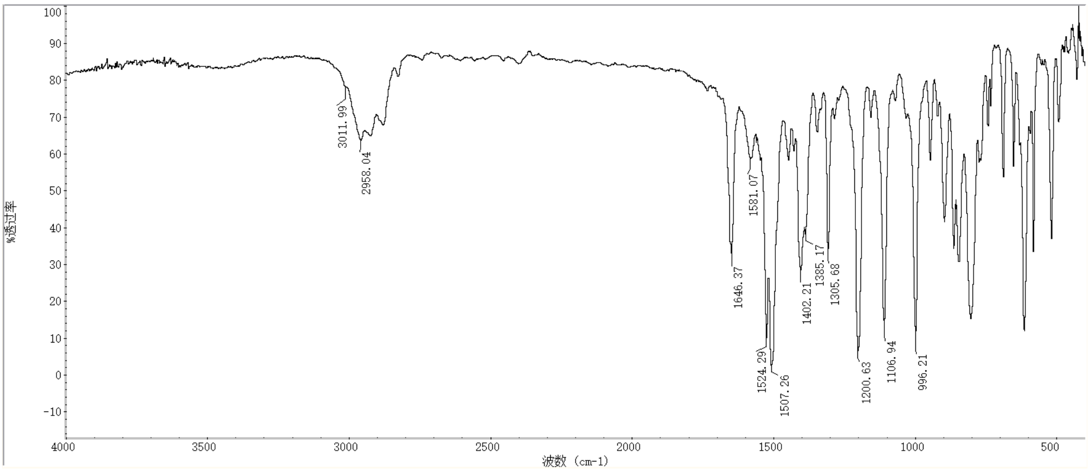

Figure S60. FTIR spectrum of compound (**4i**).

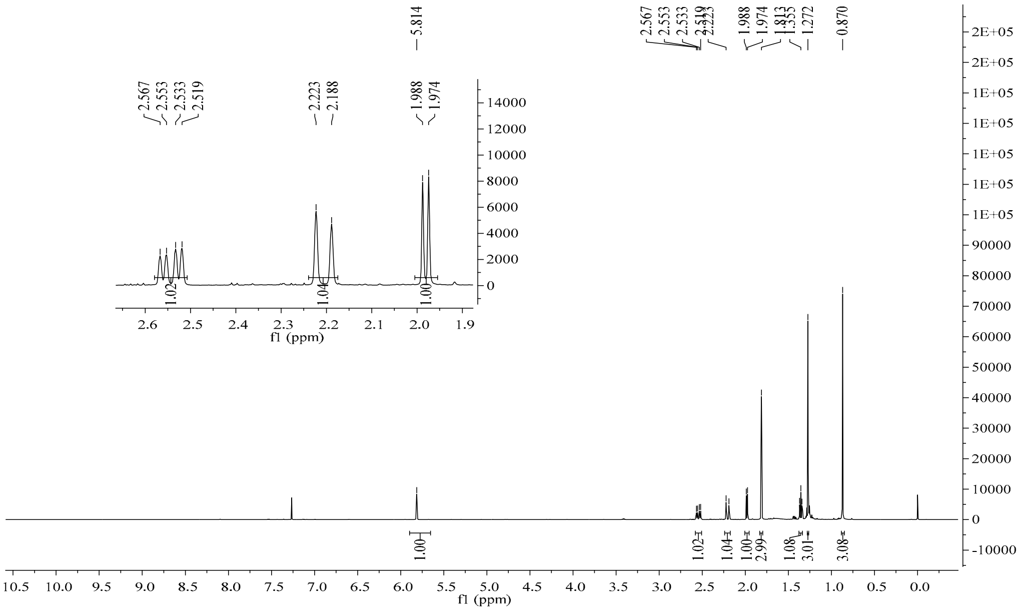

Figure S61. ^{1}H-NMR spectrum of compound (**4i**) in $CDCl_3$.

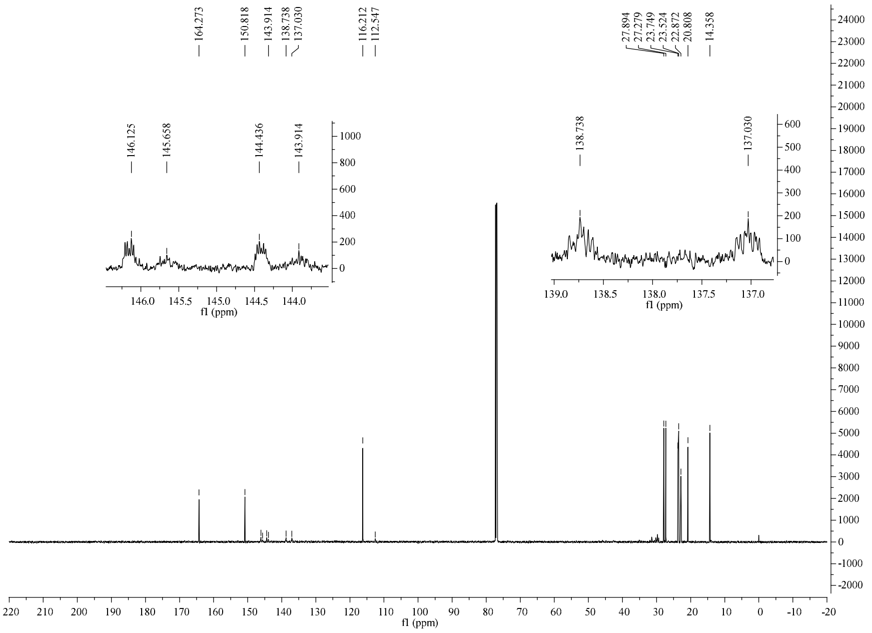

Figure S62. ^{13}C-NMR spectrum of compound (**4i**) in $CDCl_3$.

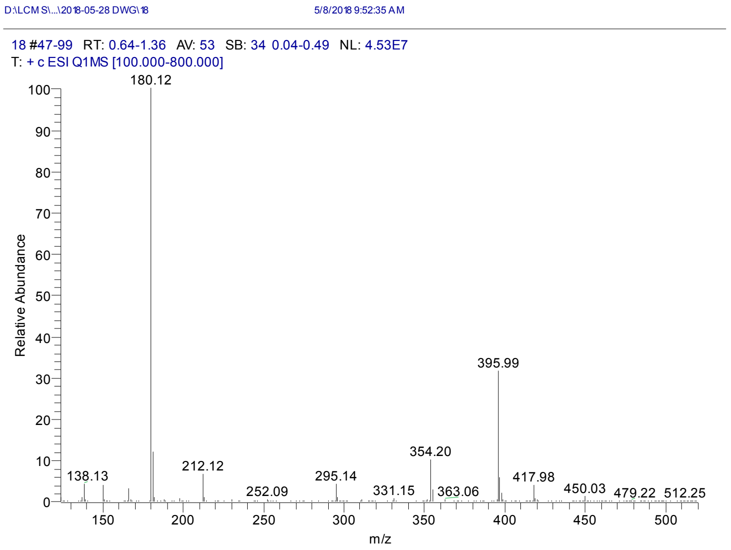

Figure S63. ESI-MS spectrum of compound (**4i).**

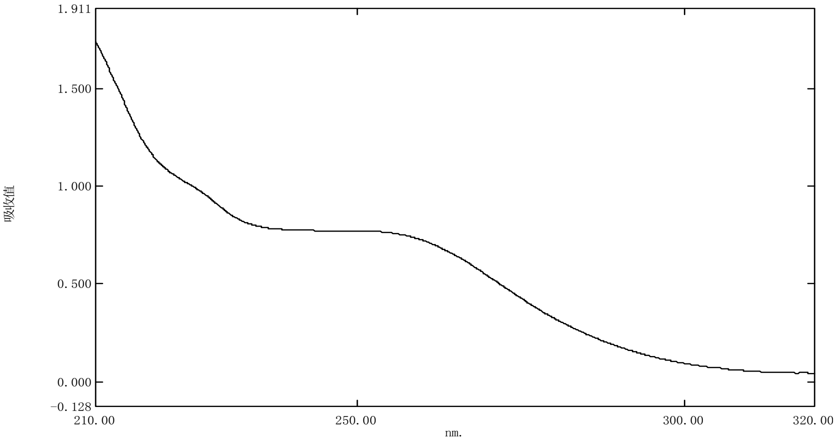

Figure S64. UV-vis spectrum of compound (**4j**) in EtOH.

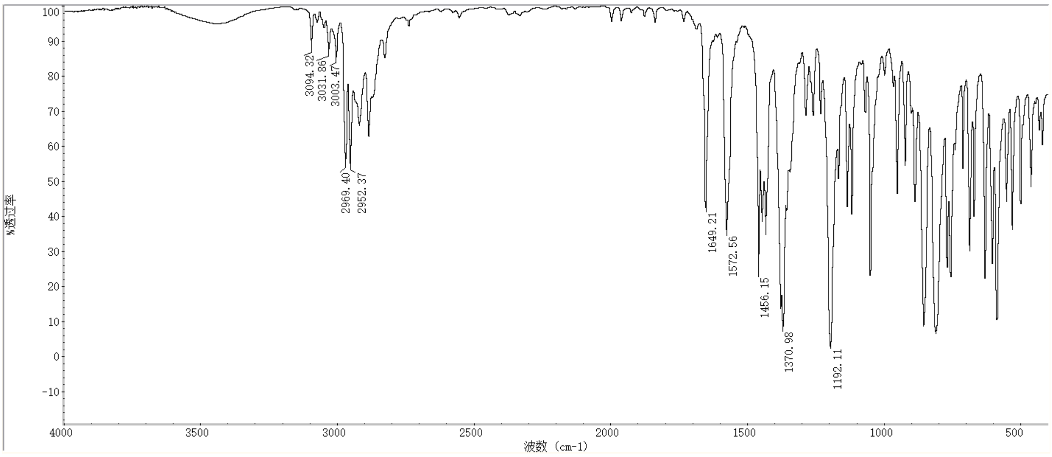

Figure S65. FTIR spectrum of compound (**4j**).

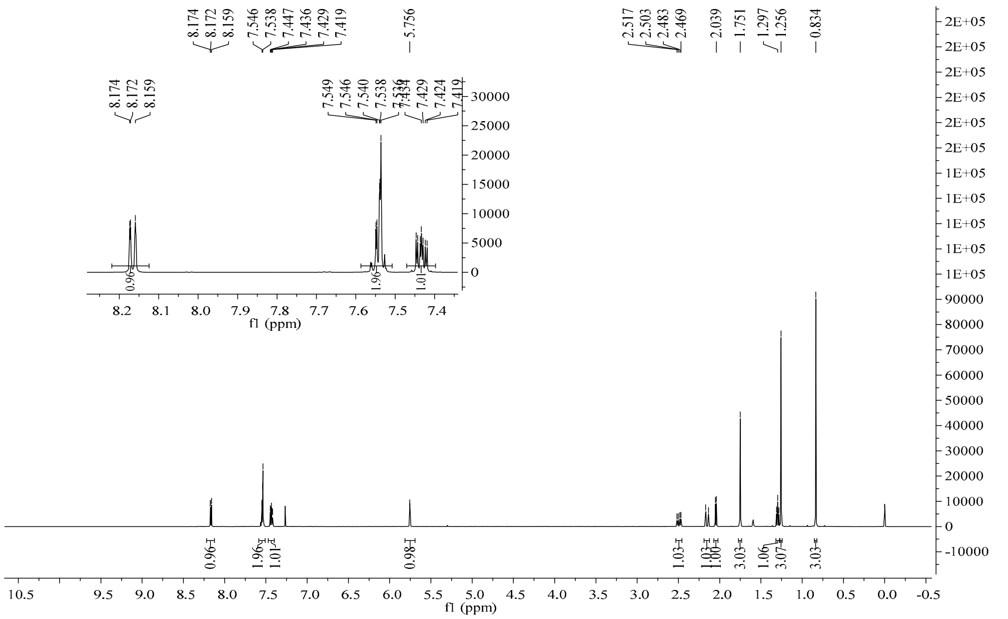

Figure S66. ^{1}H-NMR spectrum of compound (**4j**) in $CDCl_3$.

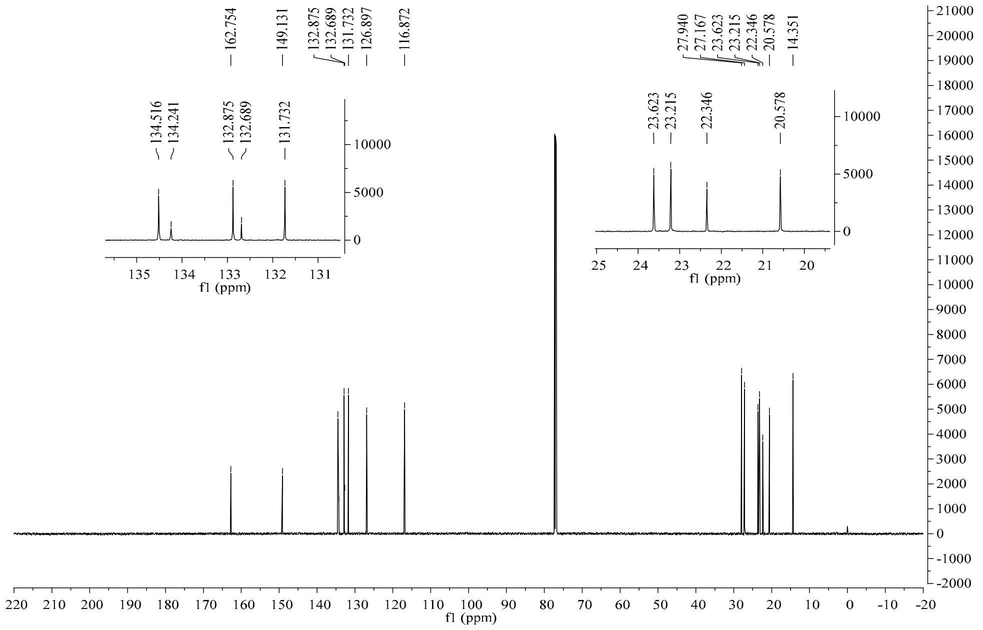

Figure S67. ^{13}C-NMR spectrum of compound (**4j**) in $CDCl_3$.

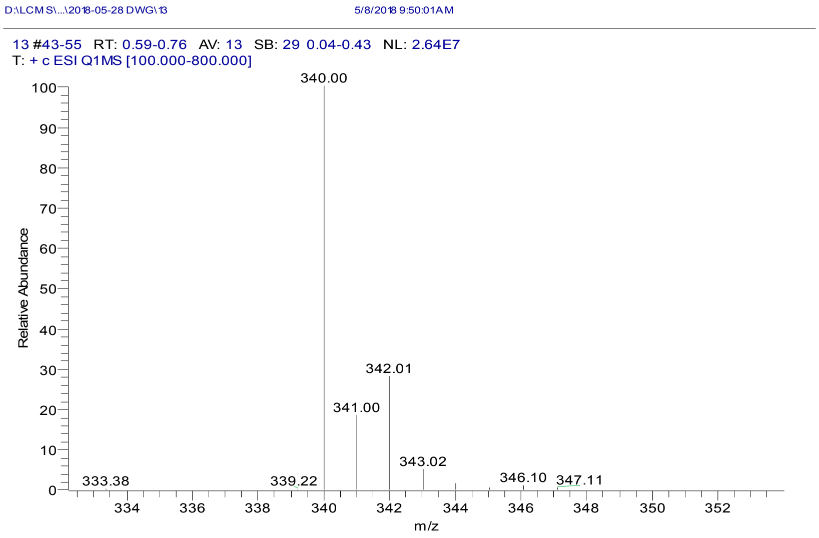

Figure S68. ESI-MS spectrum of compound (**4j).**

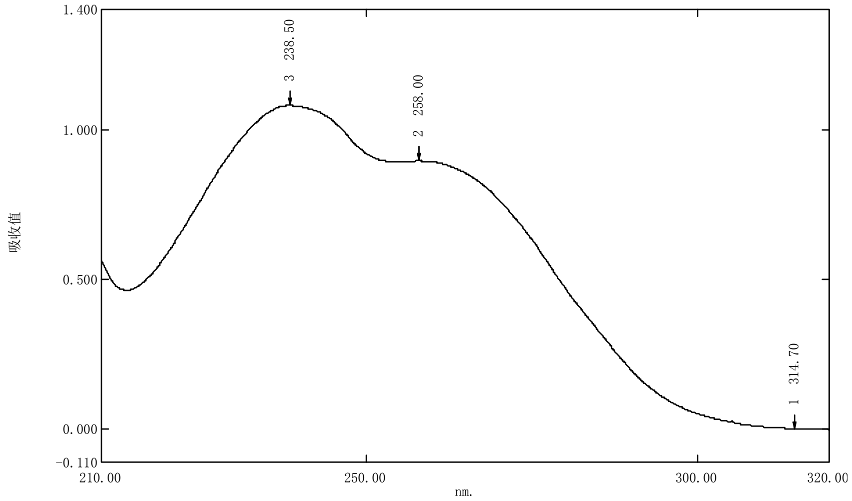

Figure S69. UV-vis spectrum of compound (**4k**) in EtOH.

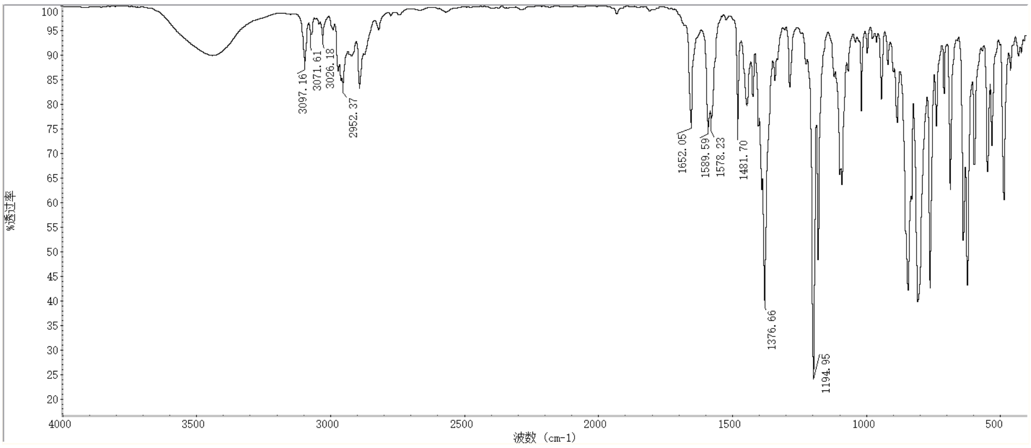

Figure S70. FTIR spectrum of compound (**4k**).

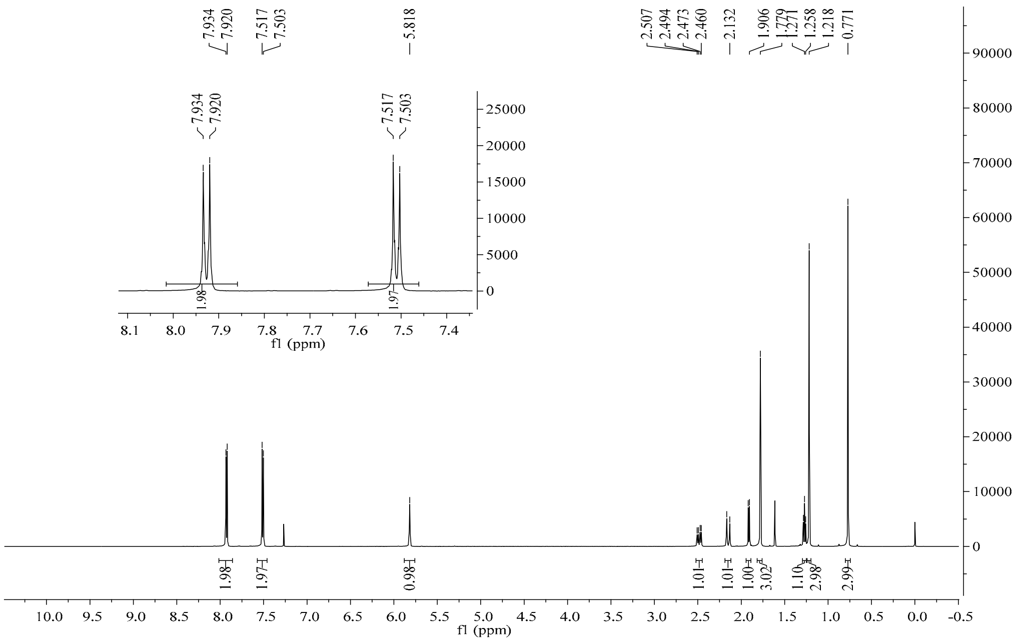

Figure S71. ^{1}H-NMR spectrum of compound (**4k**) in $CDCl_3$.

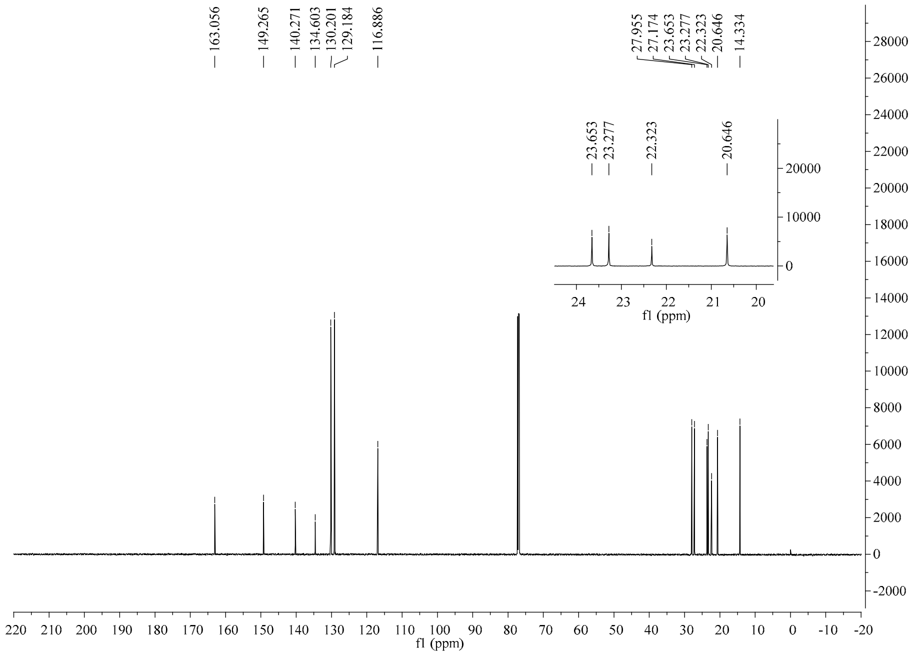

Figure S72. ^{13}C-NMR spectrum of compound (**4k**) in $CDCl_3$.

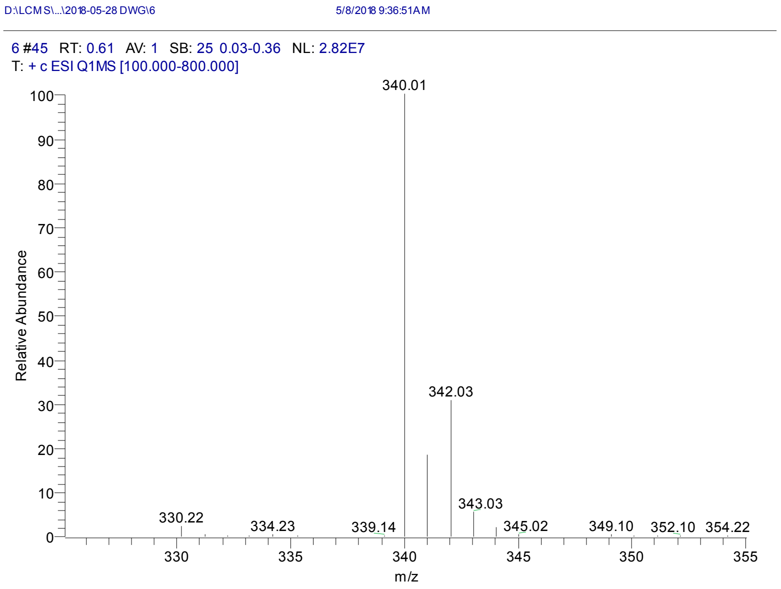

Figure S73. ESI-MS spectrum of compound (**4k).**

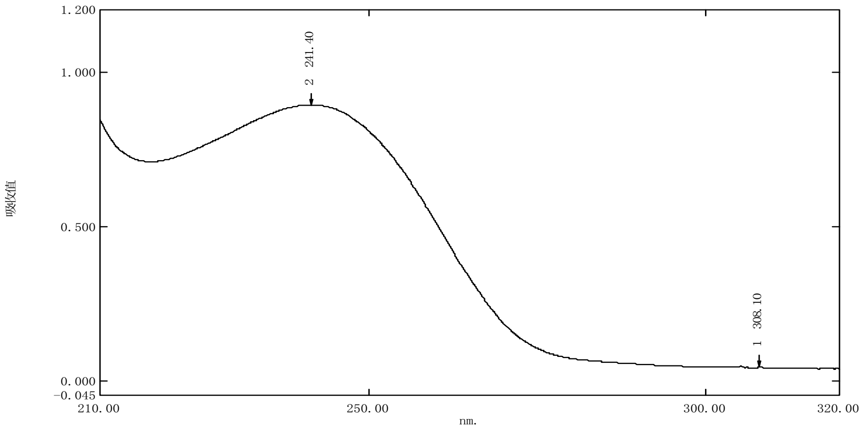

Figure S74. UV-vis spectrum of compound (**4l**) in EtOH.

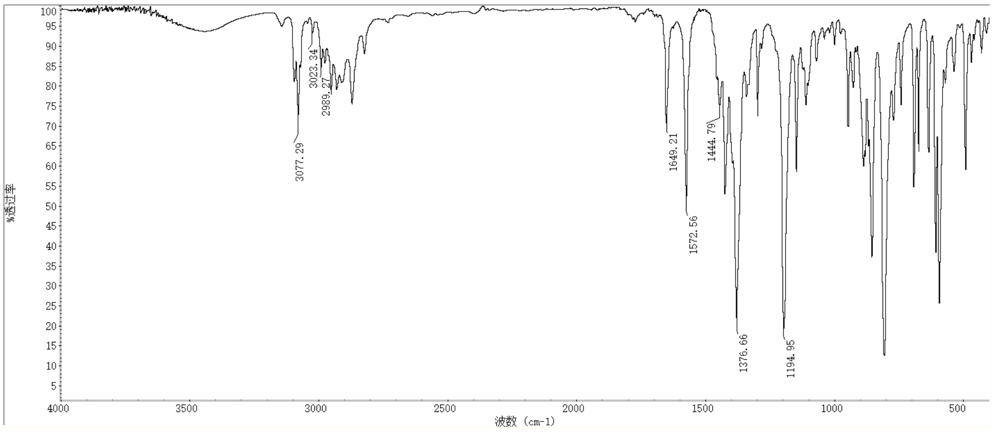

Figure S75. FTIR spectrum of compound (**4l**).

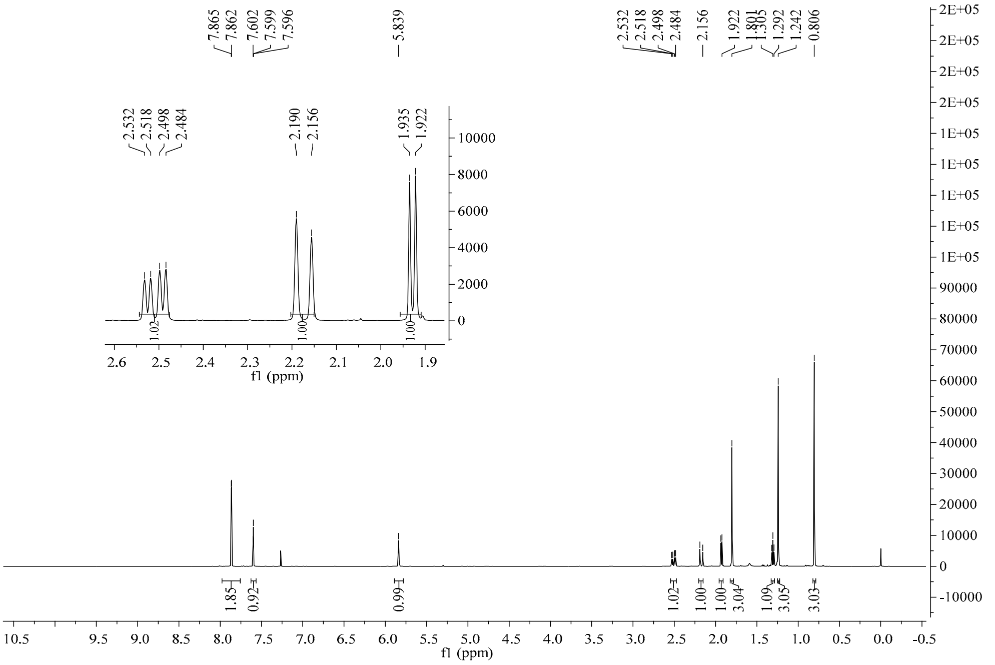

Figure S76. ^{1}H-NMR spectrum of compound (**4l**) in $CDCl_3$.

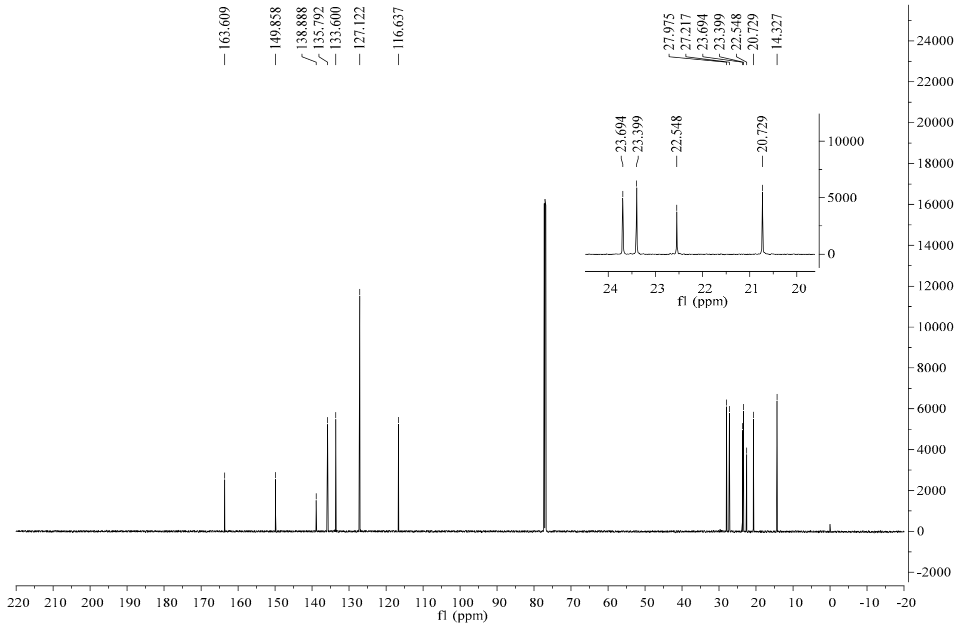

Figure S77. ^{13}C-NMR spectrum of compound (**4l**) in $CDCl_3$.

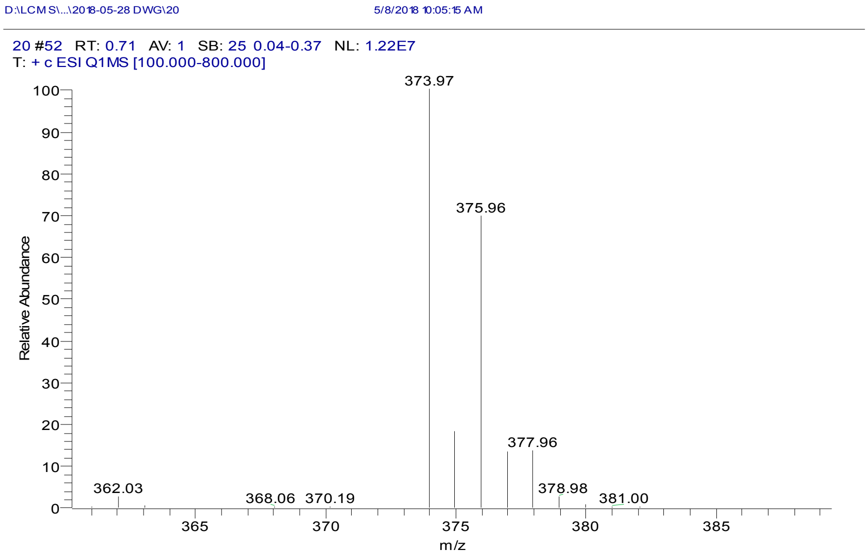

Figure S78. ESI-MS spectrum of compound (**4l).**

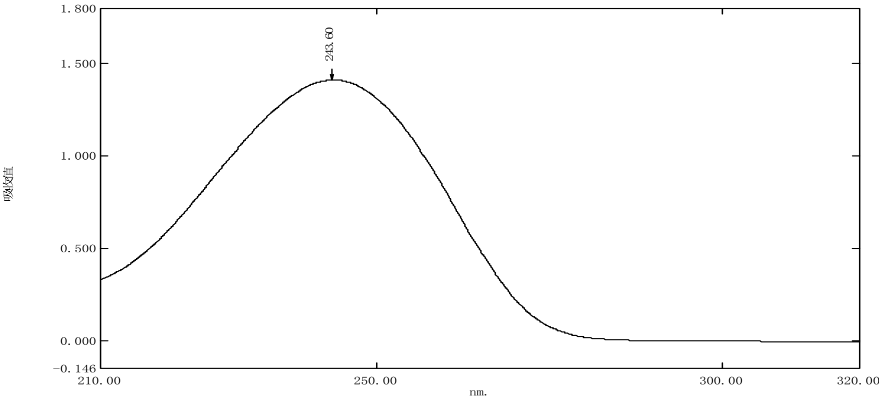

Figure S79. UV-vis spectrum of compound (**4m**) in EtOH.

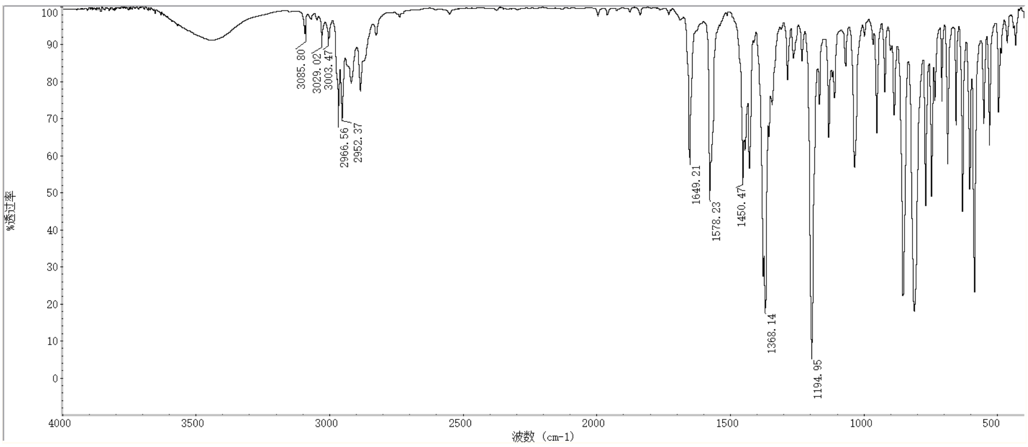

Figure S80. FTIR spectrum of compound (**4m**).

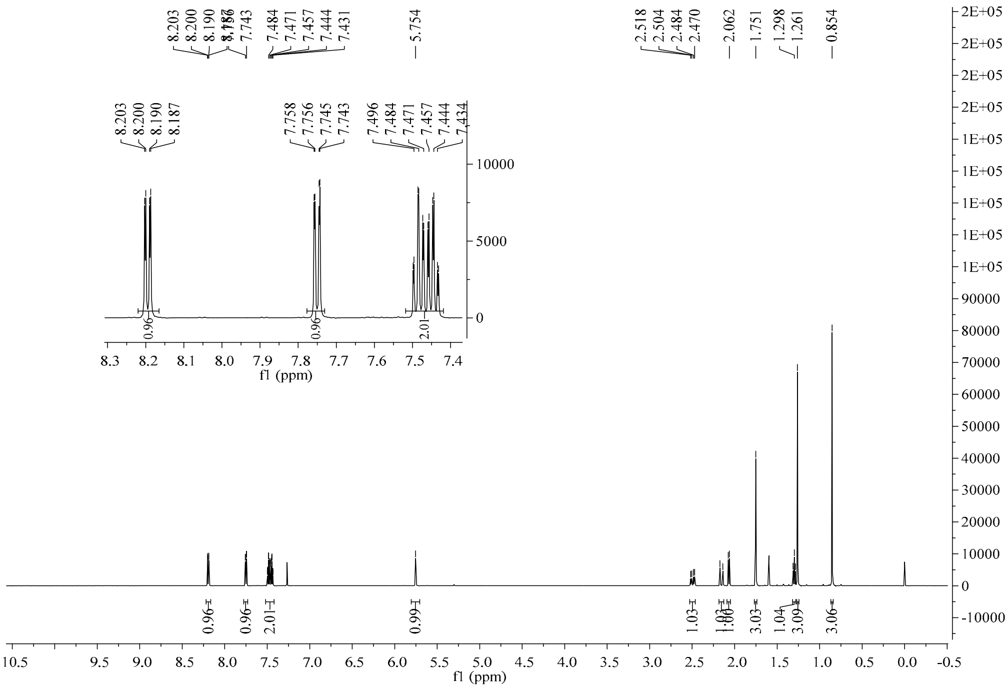

Figure S81. ^{1}H-NMR spectrum of compound (**4m**) in $CDCl_3$.

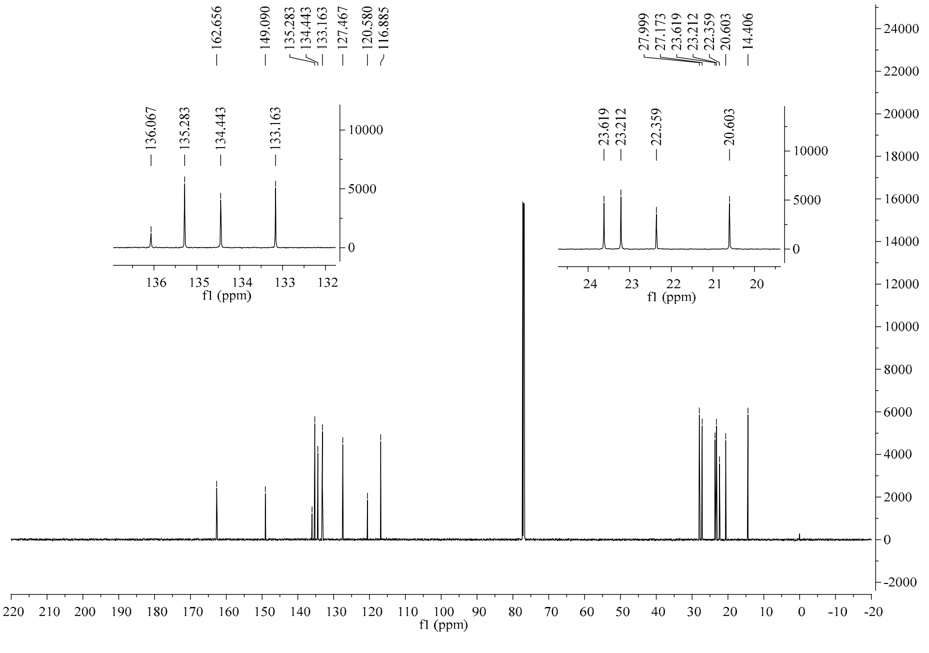

Figure S82. ^{13}C-NMR spectrum of compound (**4m**) in $CDCl_3$.

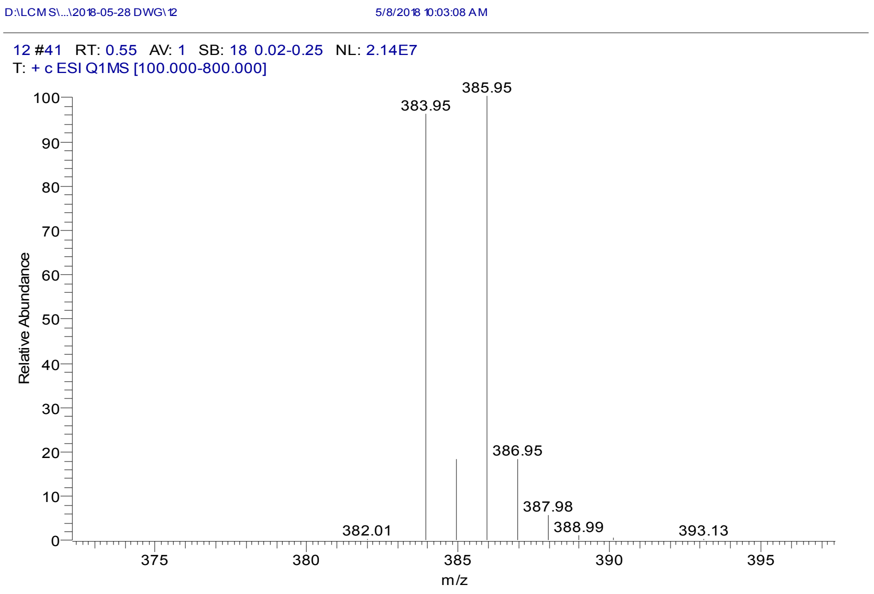

Figure S83. ESI-MS spectrum of compound (**4m).**

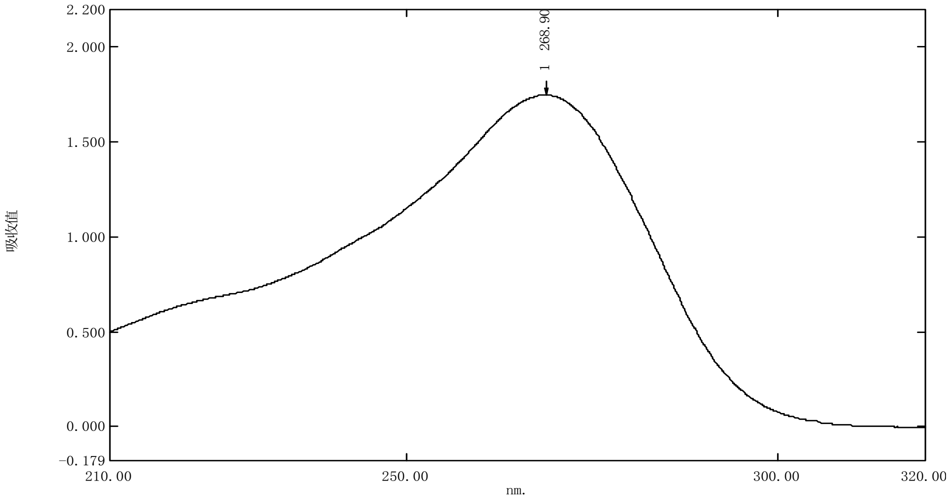

Figure S84. UV-vis spectrum of compound (**4n**) in EtOH.

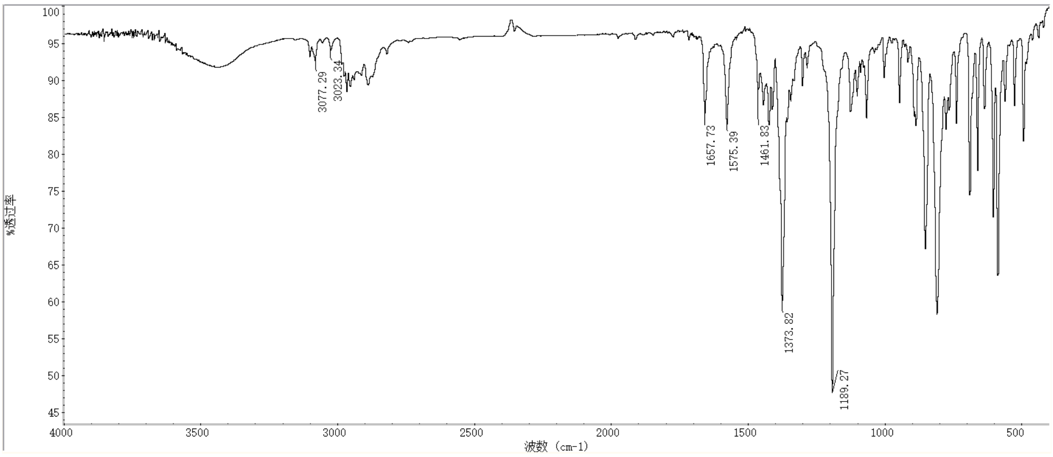

Figure S85. FTIR spectrum of compound (**4n**).

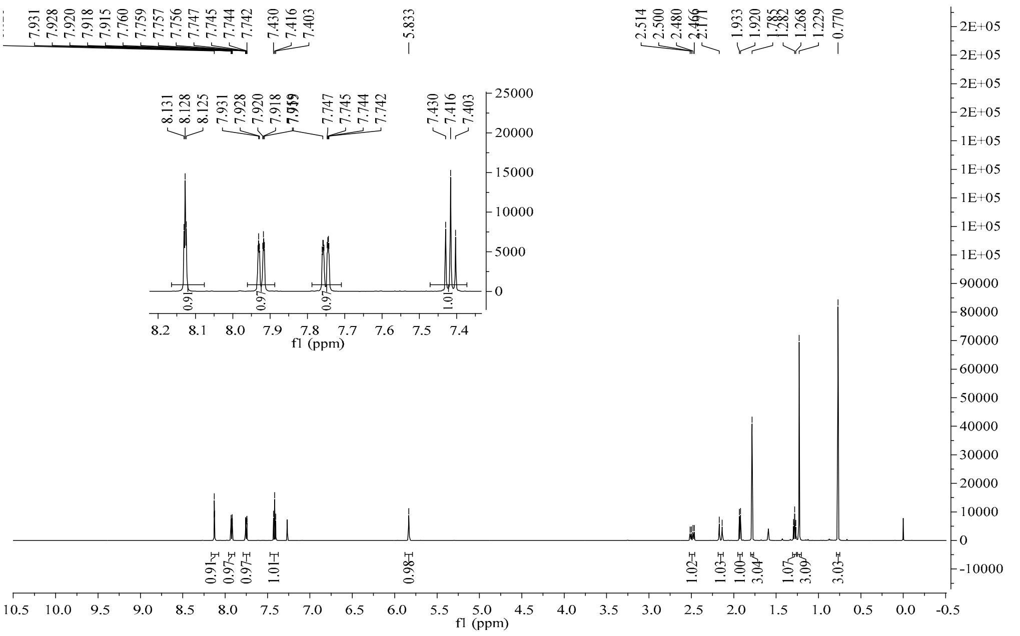

Figure S86. ^{1}H-NMR spectrum of compound (**4n**) in $CDCl_3$.

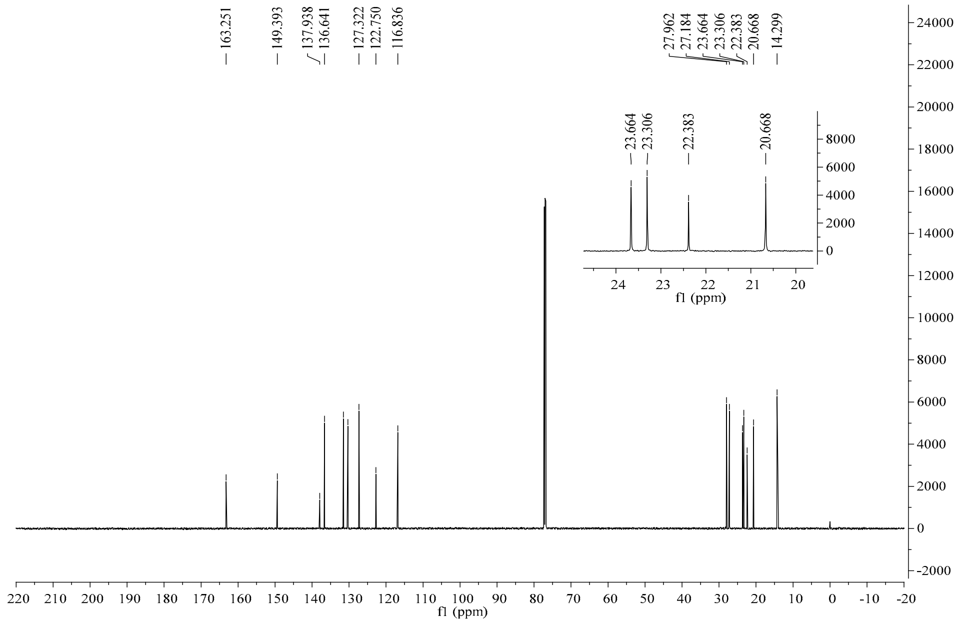

Figure S87. ^{13}C-NMR spectrum of compound (**4n**) in $CDCl_3$.

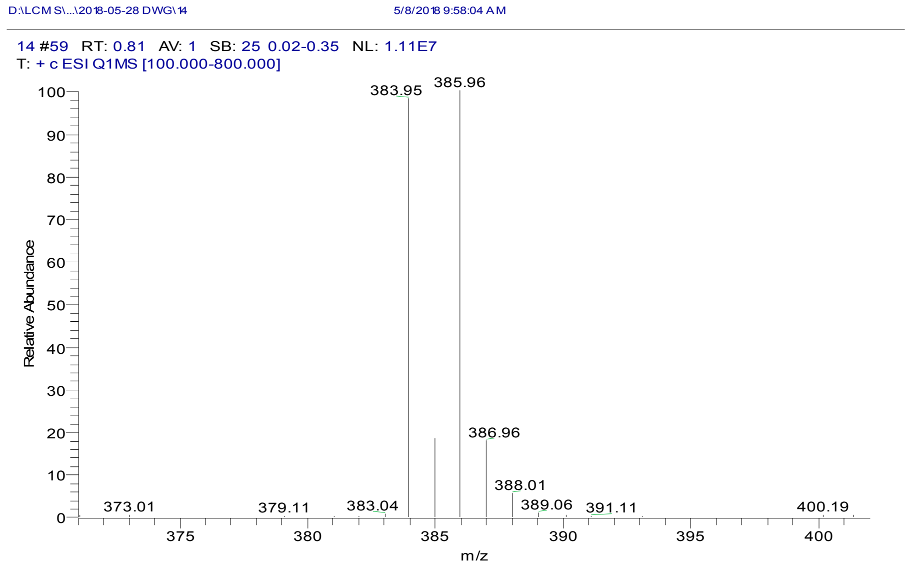

Figure S88. ESI-MS spectrum of compound (**4n).**

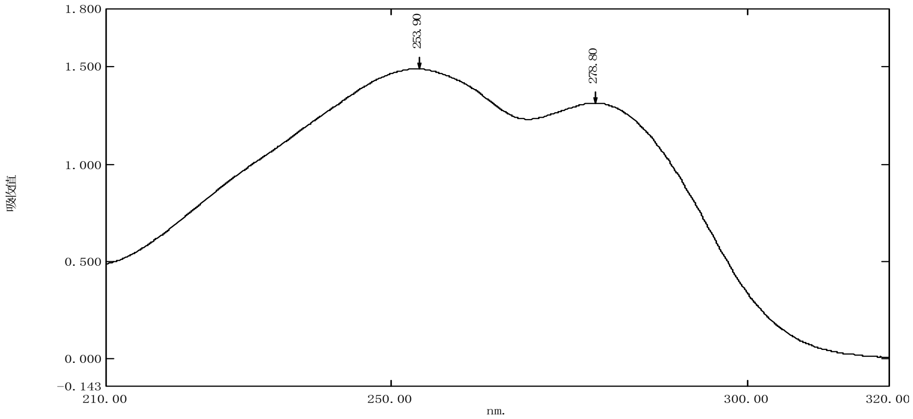

Figure S89. UV-vis spectrum of compound (**4o**) in EtOH.

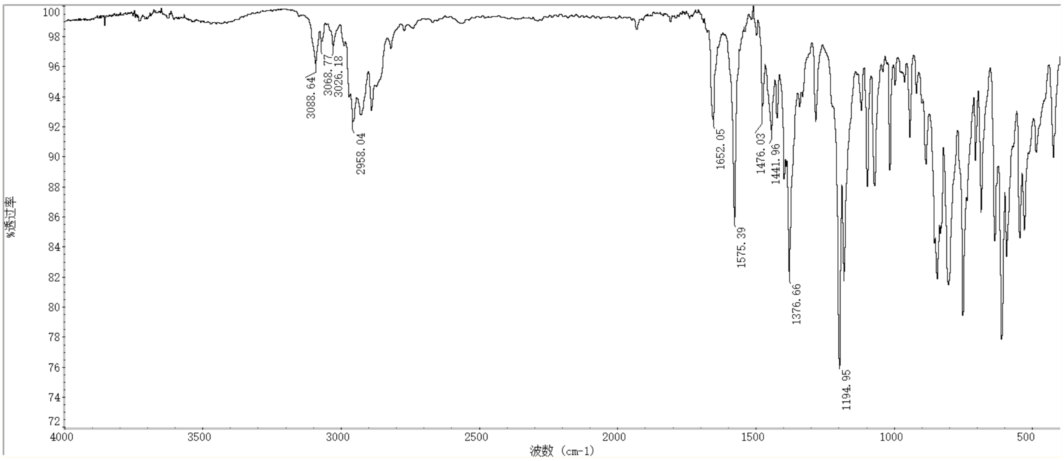

Figure S90. FTIR spectrum of compound (**4o**).

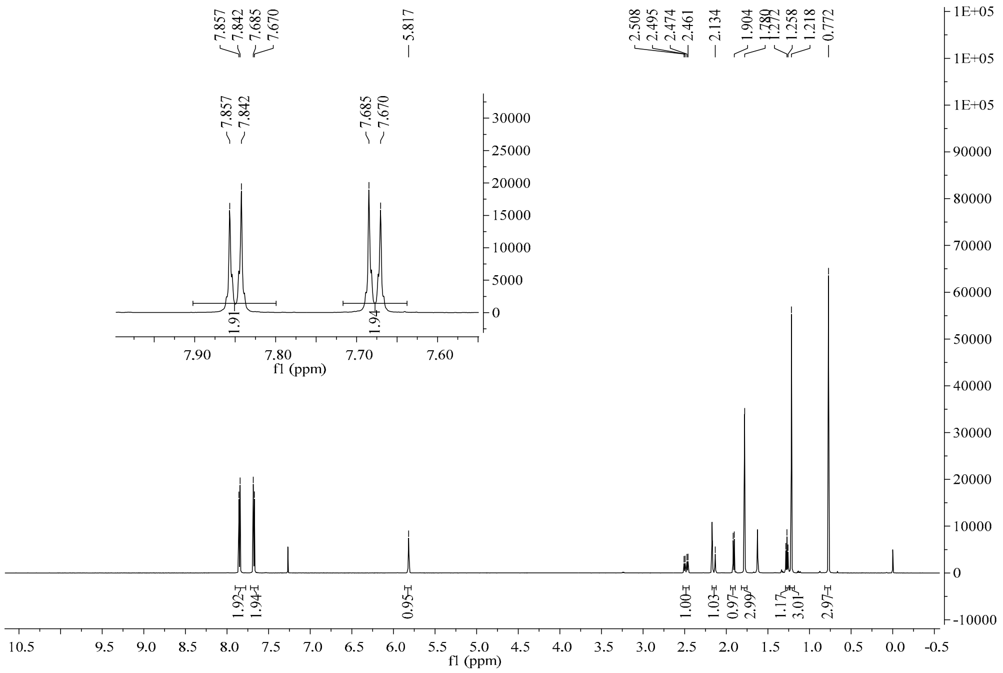

Figure S91. ^{1}H-NMR spectrum of compound (**4o**) in $CDCl_3$.

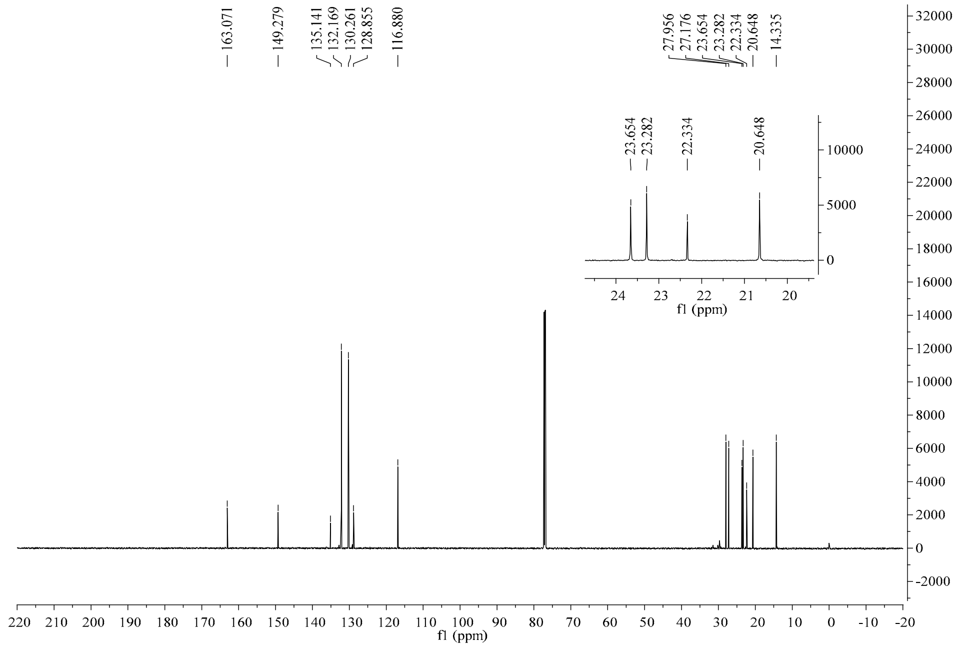

Figure S92. ^{13}C-NMR spectrum of compound (**4o**) in $CDCl_3$.

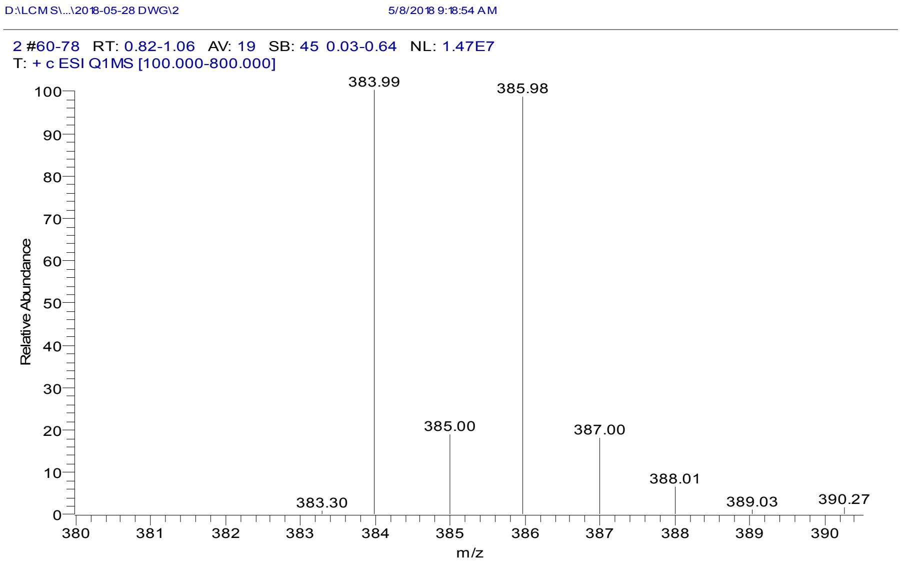

Figure S93. ESI-MS spectrum of compound (**4o).**

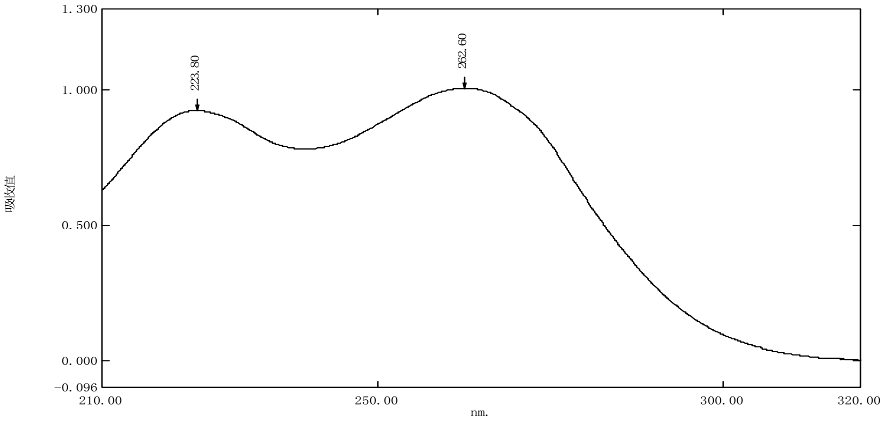

Figure S94. UV-vis spectrum of compound (**4p**) in EtOH.

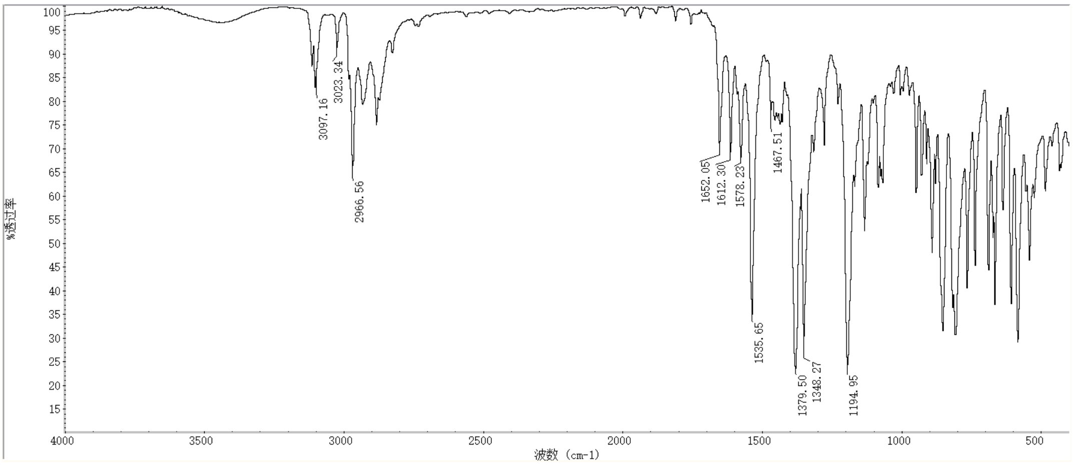

Figure S95. FTIR spectrum of compound (**4p**).

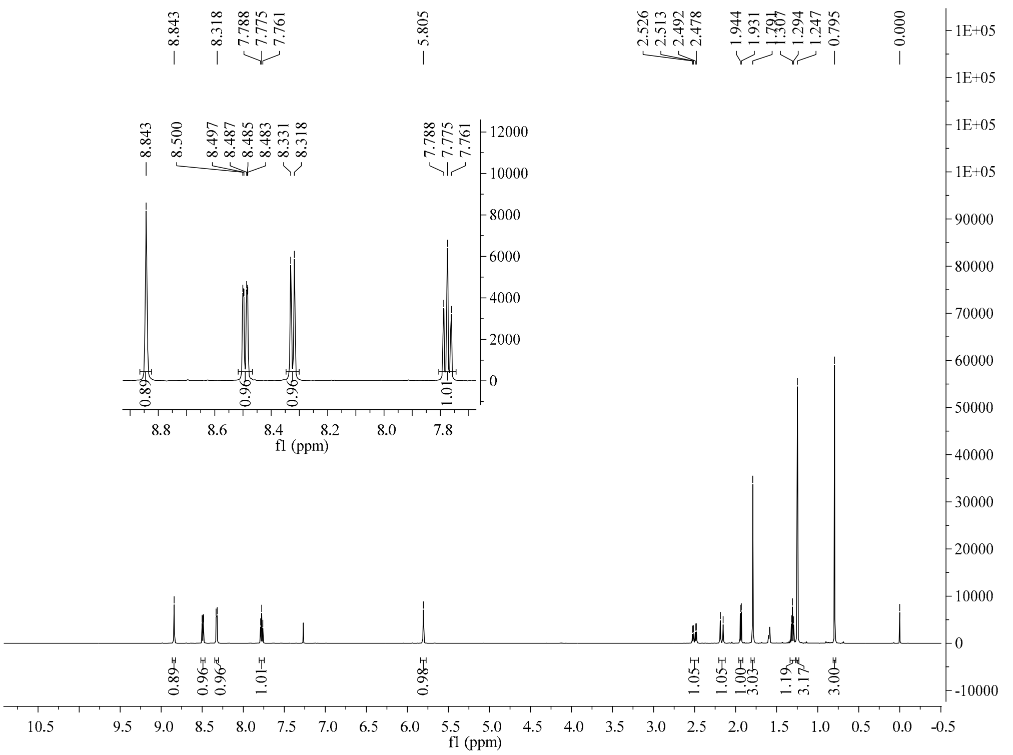

Figure S96. ^{1}H-NMR spectrum of compound (**4p**) in $CDCl_3$.

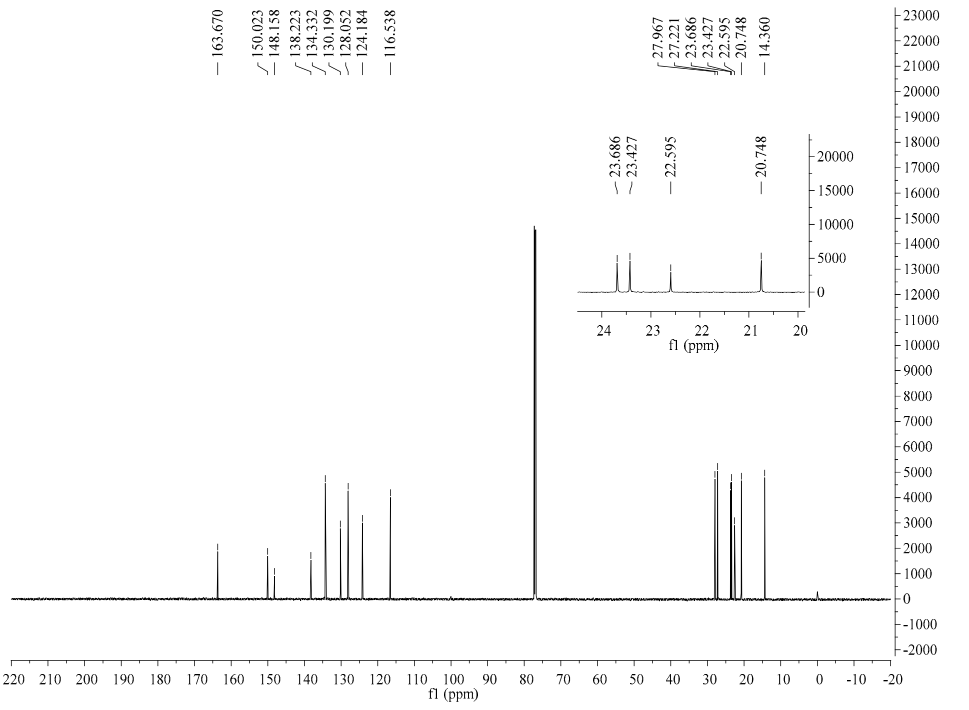

Figure S97. ^{13}C-NMR spectrum of compound (**4p**) in $CDCl_3$.

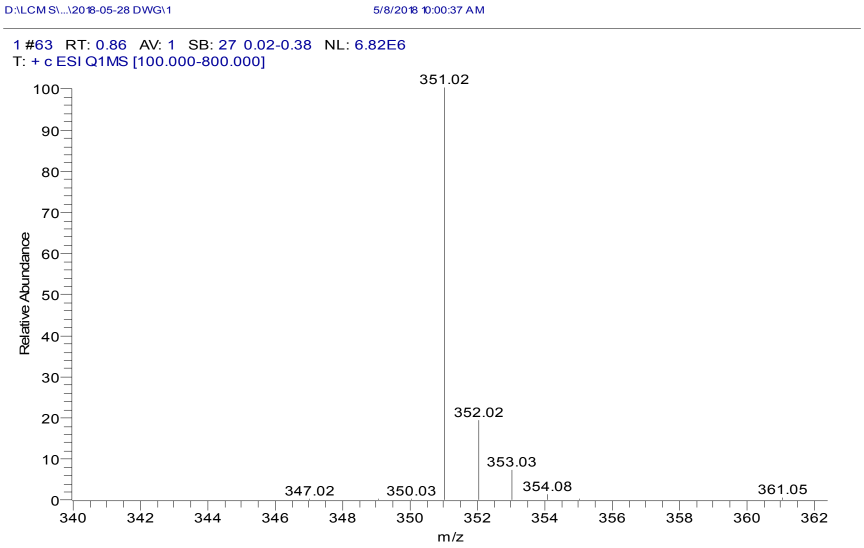

Figure S98. ESI-MS spectrum of compound (**4p).**

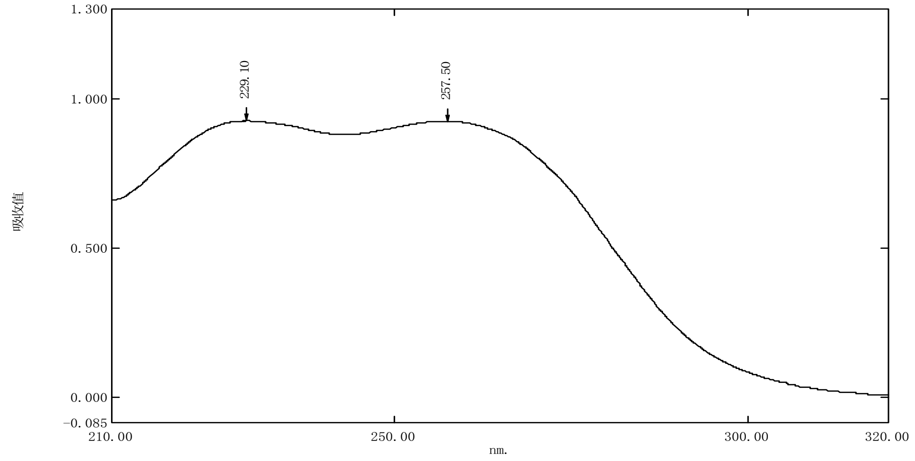

Figure S99. UV-vis spectrum of compound (**4q**) in EtOH.

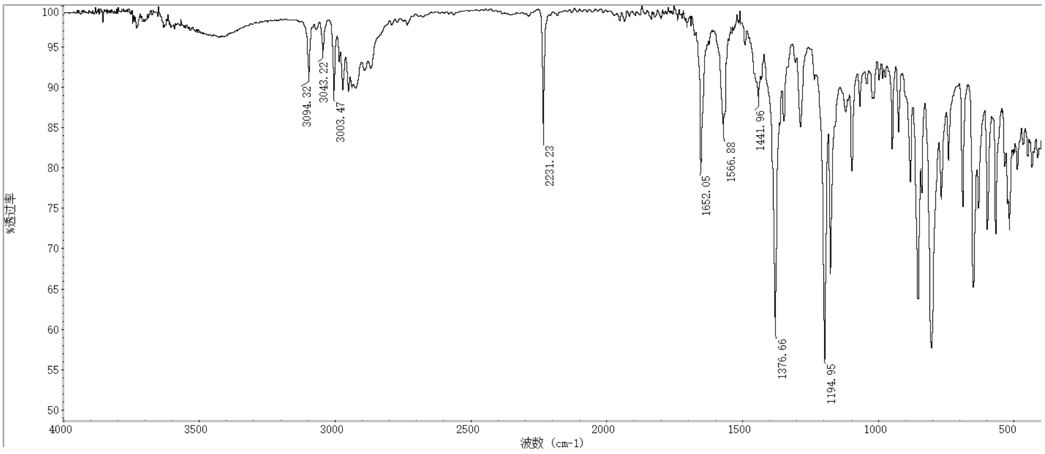

Figure S100. FTIR spectrum of compound (**4q**).

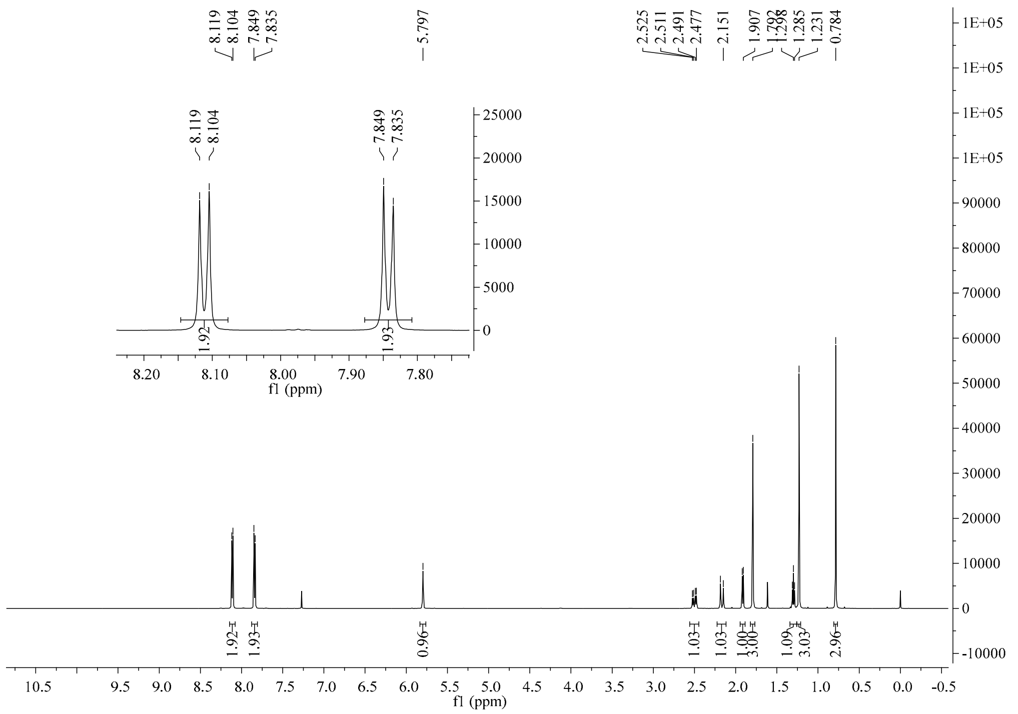

Figure S101. ^{1}H-NMR spectrum of compound (**4q**) in $CDCl_3$.

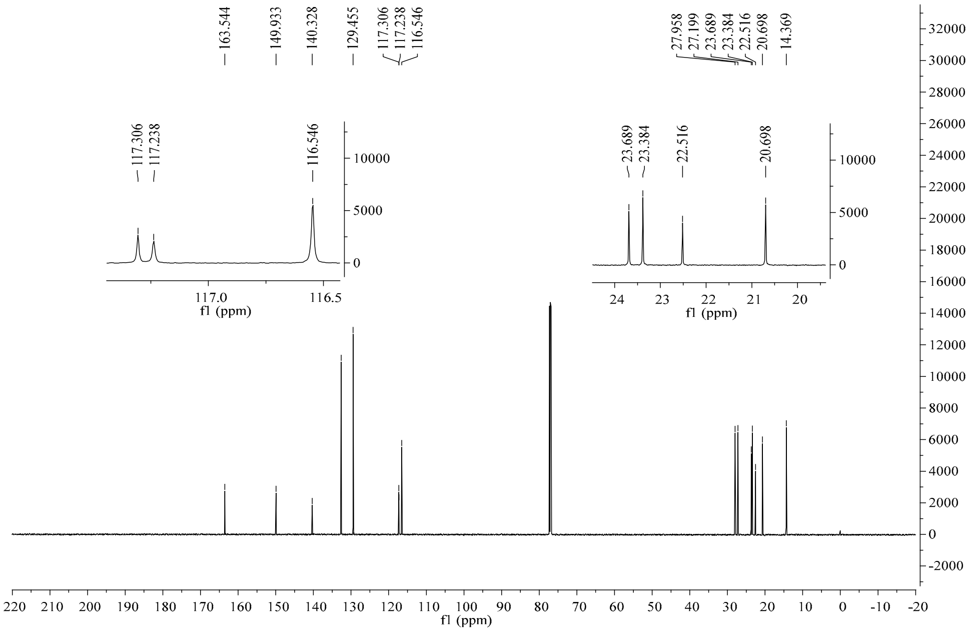

Figure S102. ^{13}C-NMR spectrum of compound (**4q)** in $CDCl_3$.

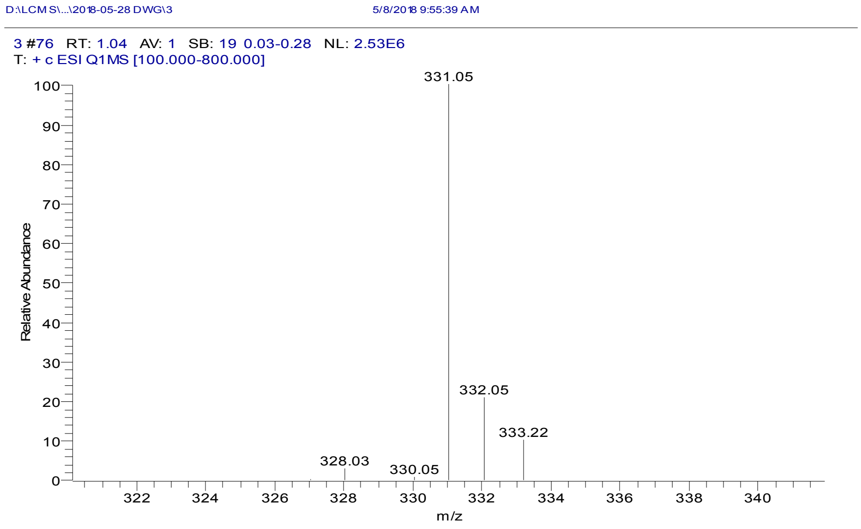

Figure S103. ESI-MS spectrum of compound (**4q).**

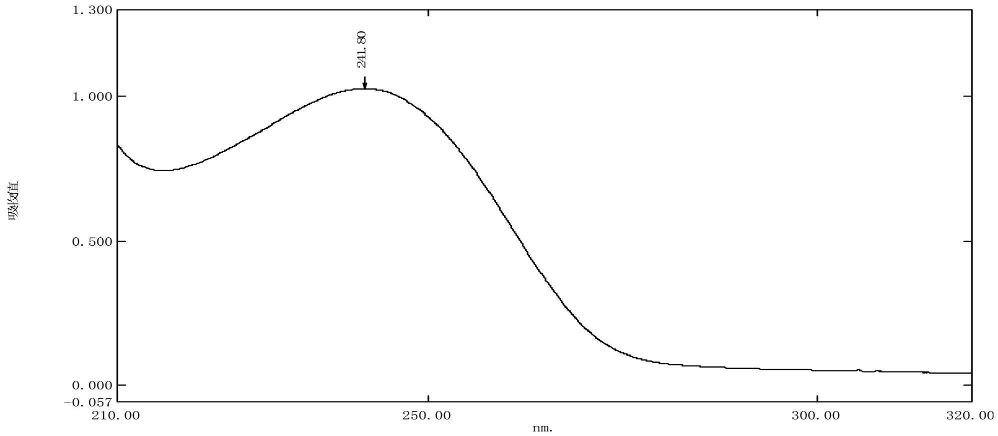

Figure S104. UV-vis spectrum of compound (**4r**) in EtOH.

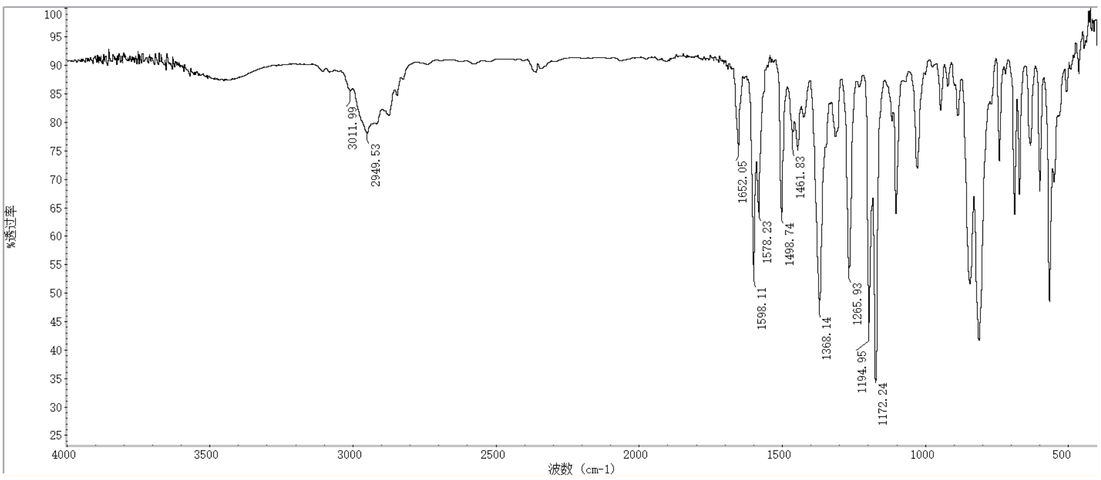

Figure S105. FTIR spectrum of compound (**4r**).

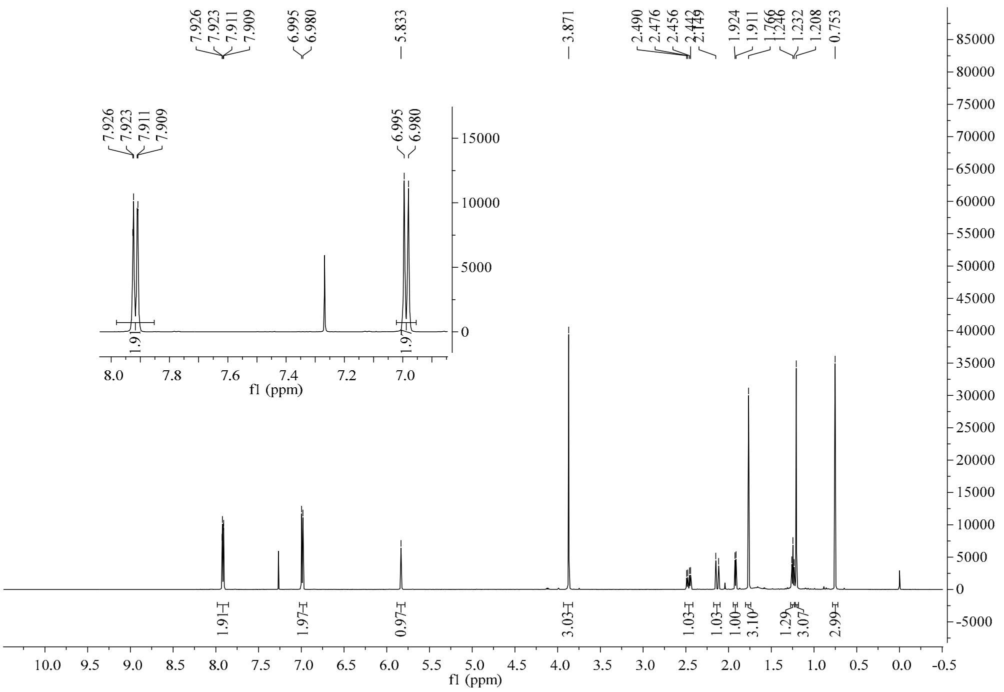

Figure S106. ^{1}H-NMR spectrum of compound (**4r**) in $CDCl_3$.

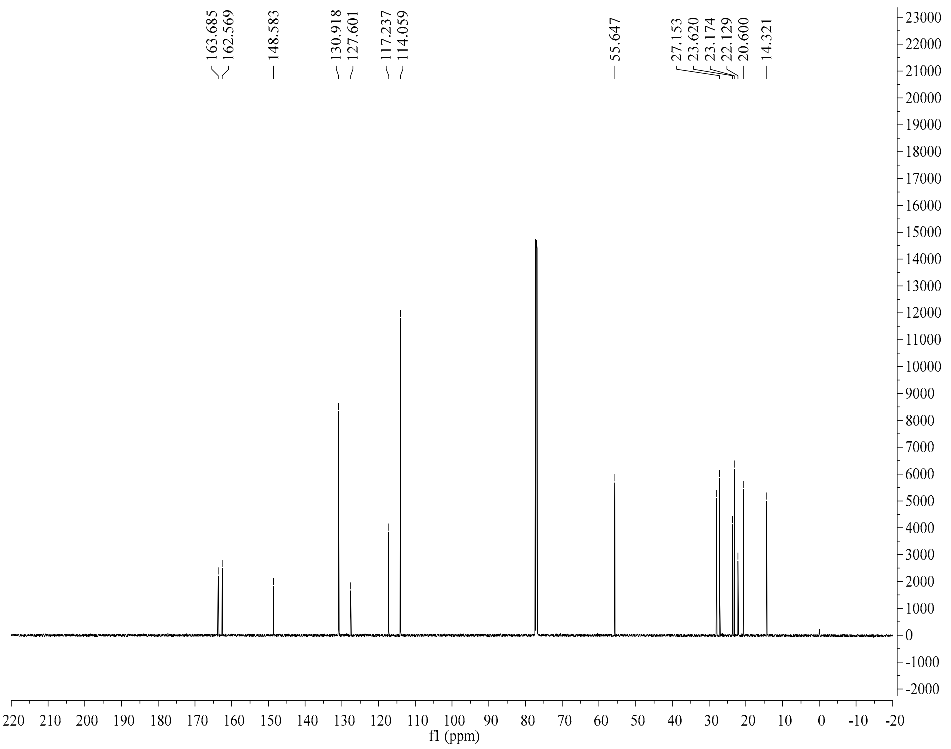

Figure S107. ^{13}C-NMR spectrum of compound (**4r**) in $CDCl_3$.

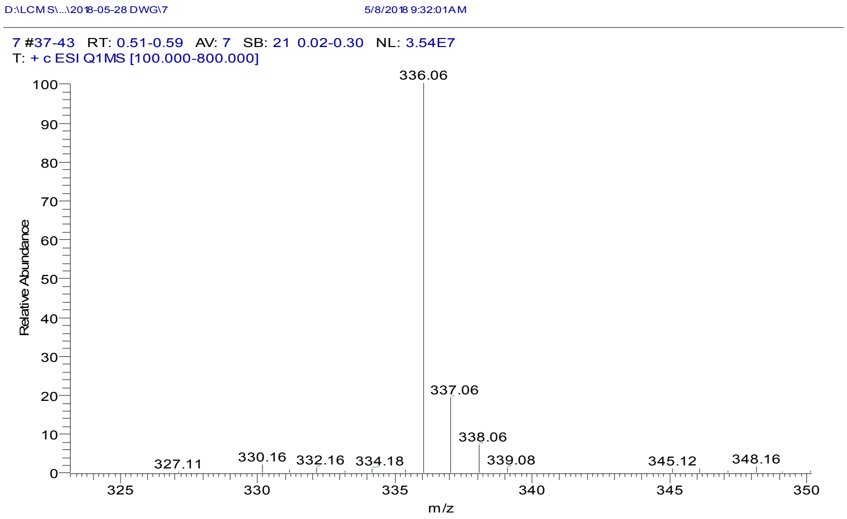

Figure S108. ESI-MS spectrum of compound (**4r).**

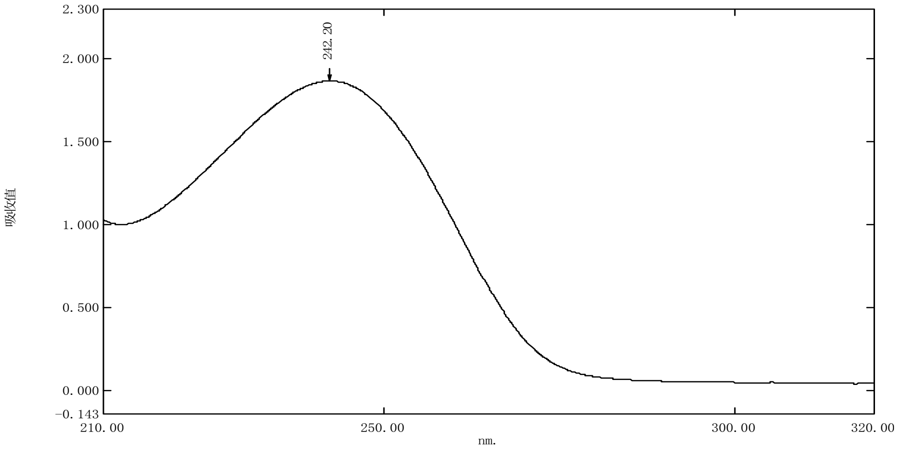

Figure S109. UV-vis spectrum of compound (**4s**) in EtOH.

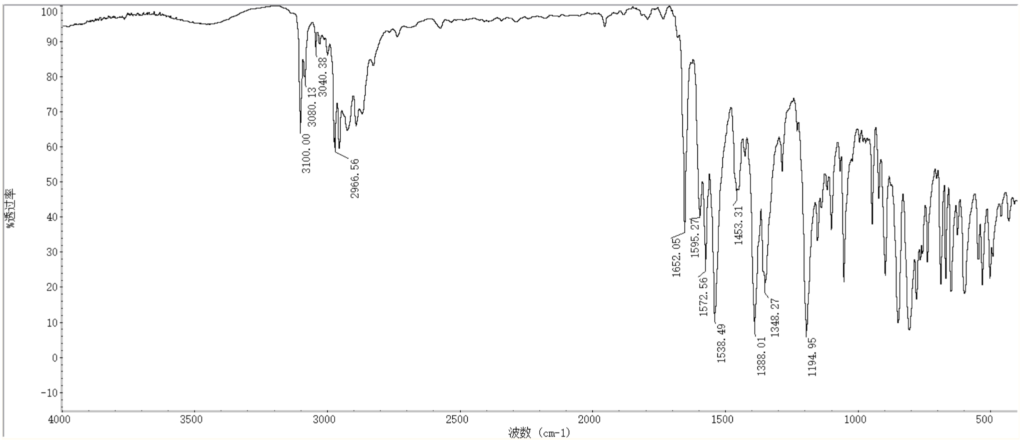

Figure S110. FTIR spectrum of compound (**4s**).

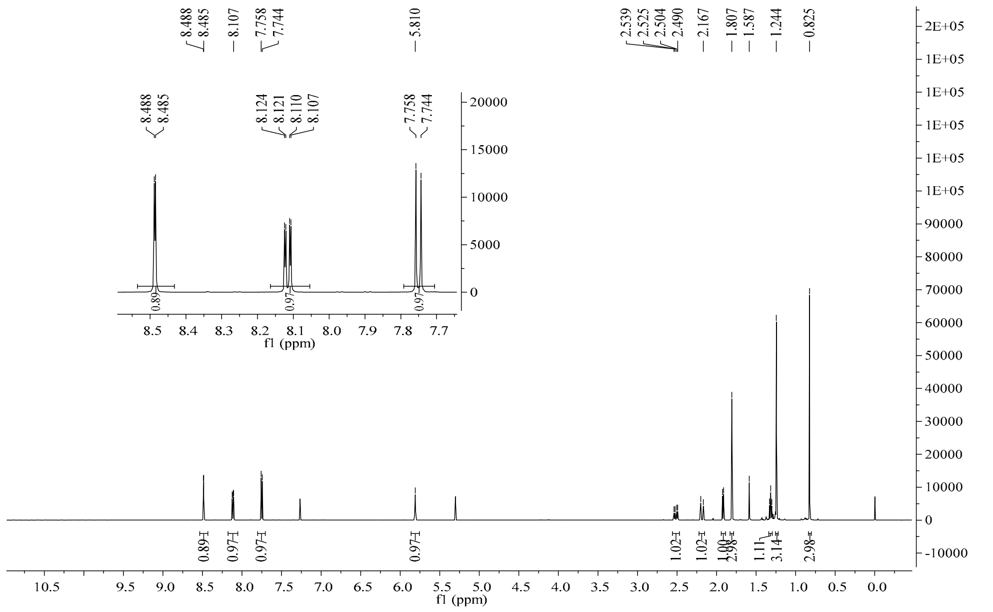

Figure S111. ^{1}H-NMR spectrum of compound (**4s)** in $CDCl_3$.

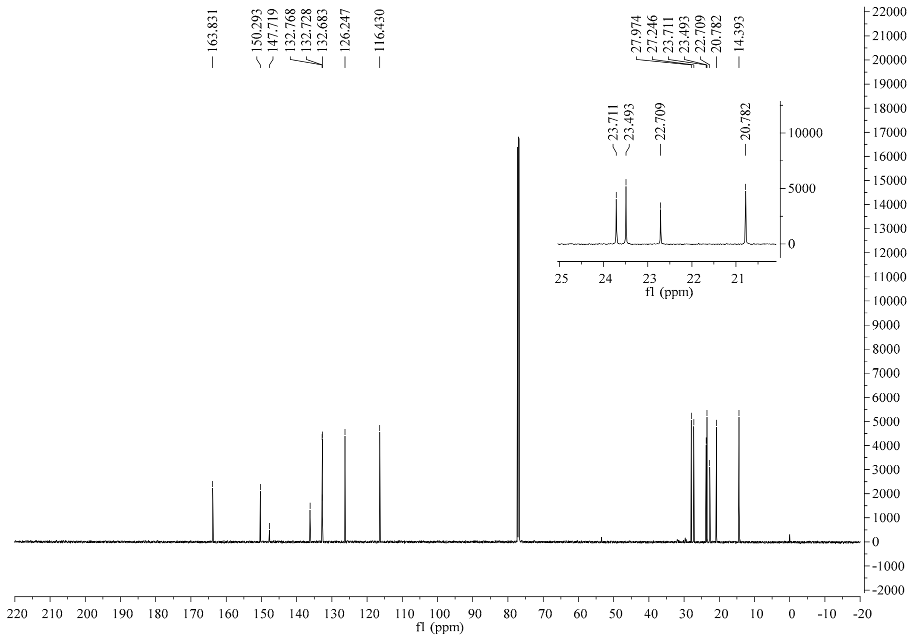

Figure S112. ^{13}C-NMR spectrum of compound (**4s)** in $CDCl_3$.

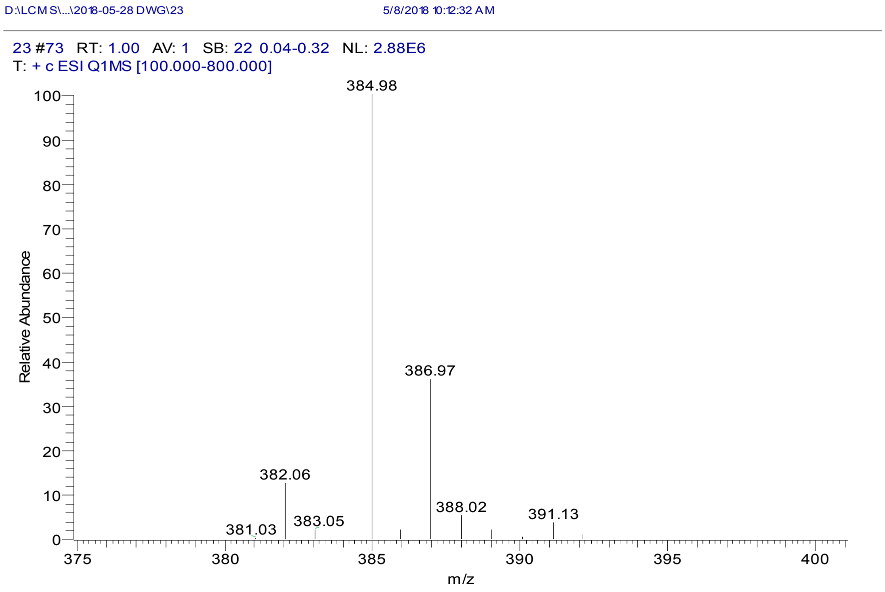

Figure S113. ESI-MS spectrum of compound (**4s).**

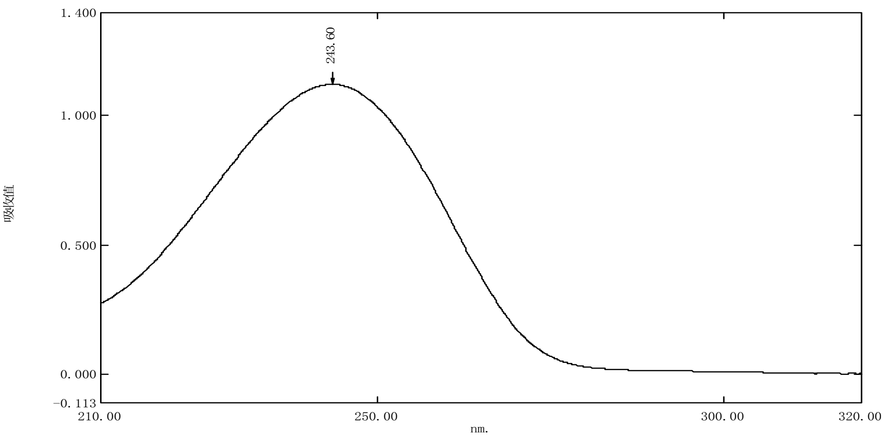

Figure S114. UV-vis spectrum of compound (**4t**) in EtOH.

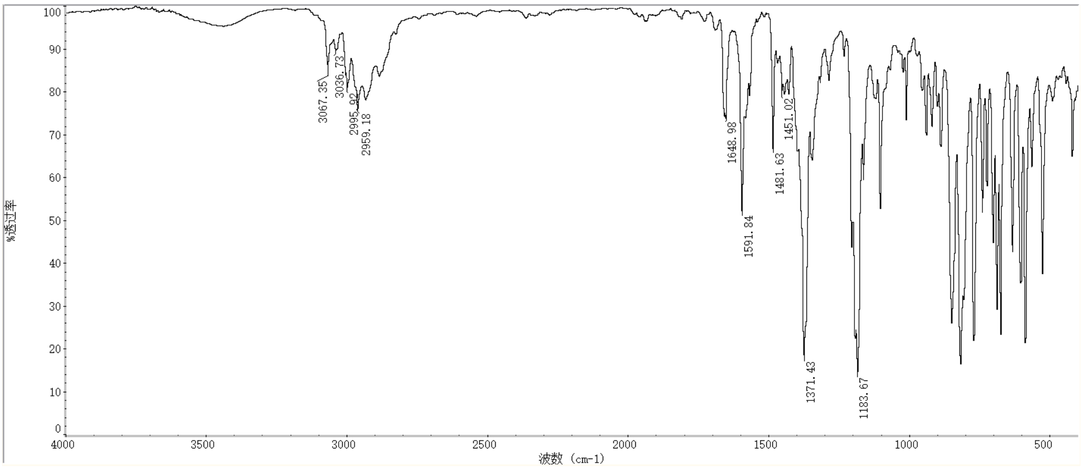

Figure S115. FTIR spectrum of compound (**4t**).

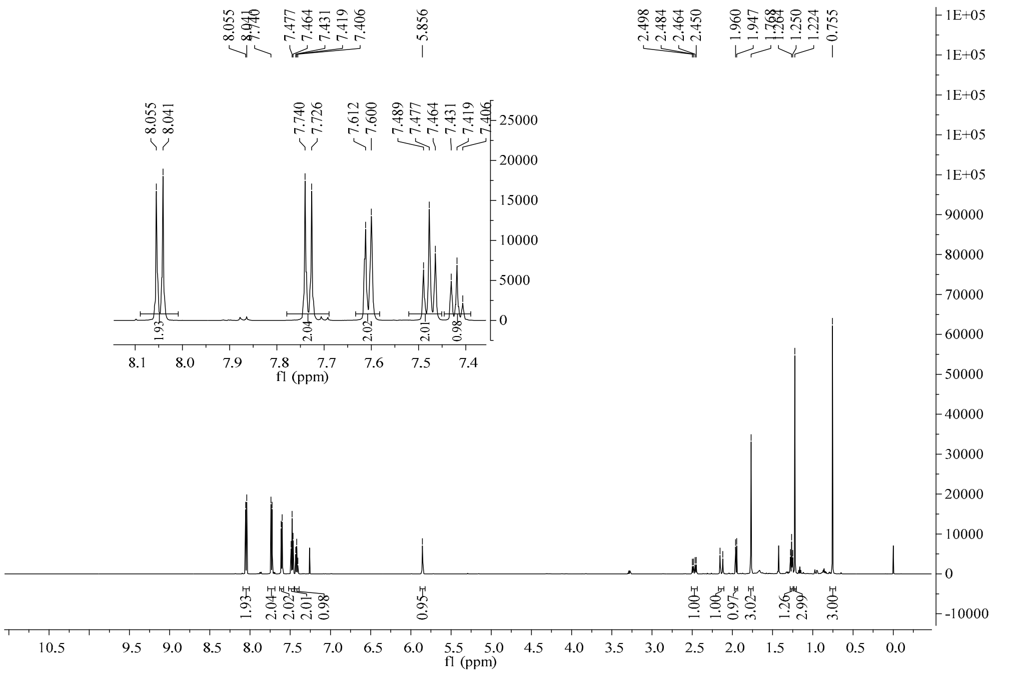

Figure S116. ^{1}H-NMR spectrum of compound (**4t**) in $CDCl_3$.

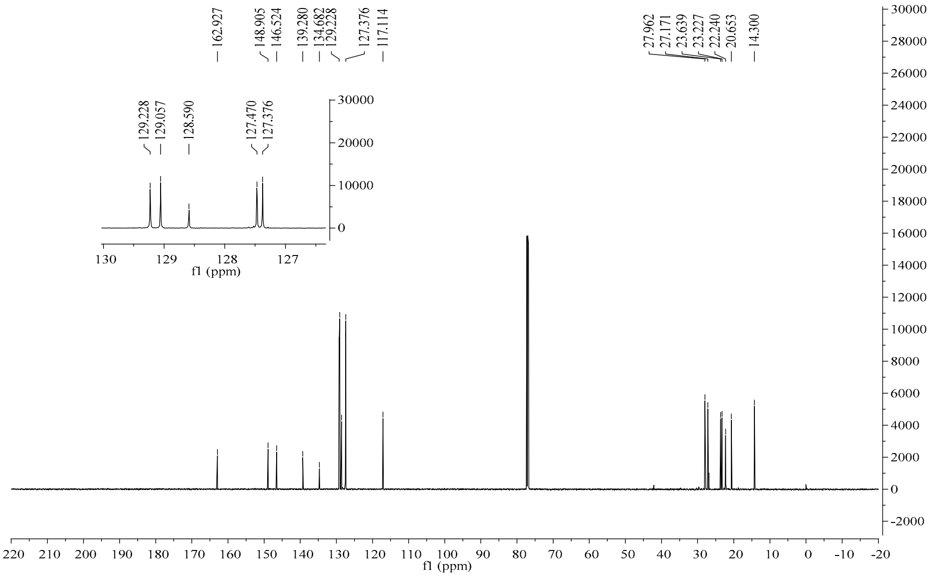

Figure S117 ^{13}C-NMR spectrum of compound (**4t**) in $CDCl_3$.

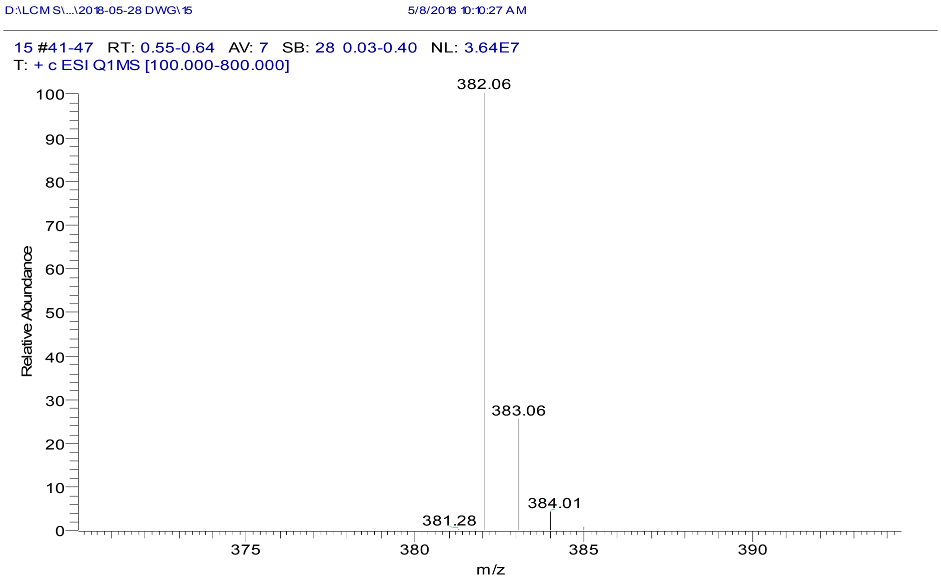

Figure S118. ESI-MS spectrum of compound (**4t**).

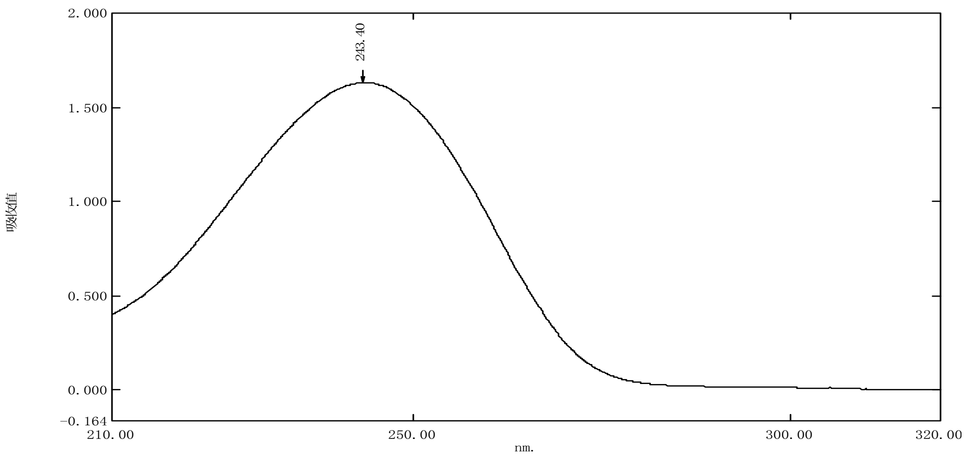

Figure S119. UV-vis spectrum of compound (**4u**) in EtOH.

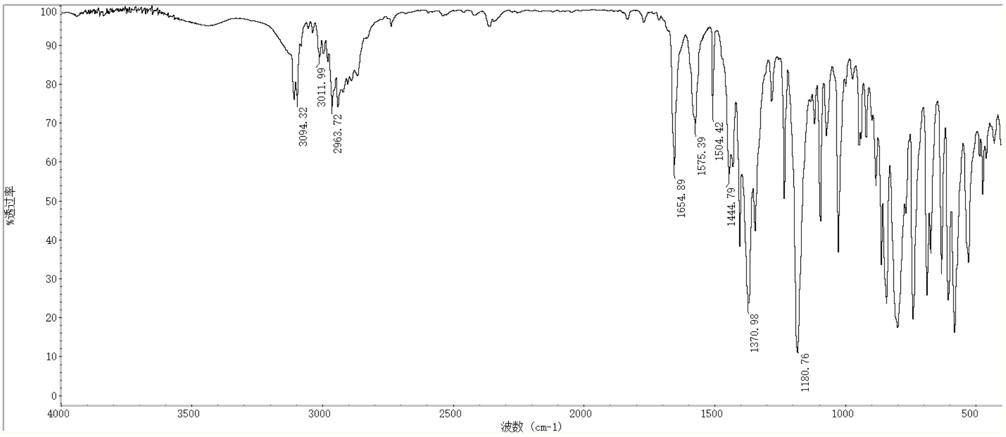

Figure S120. FTIR spectrum of compound (**4u**).

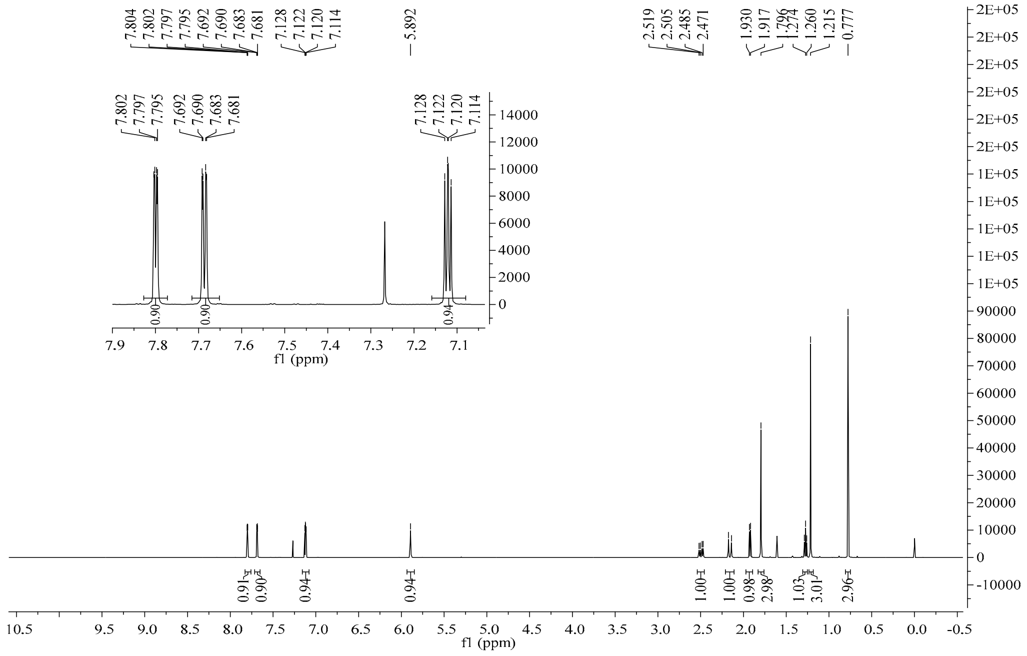

Figure S121. ^{1}H-NMR spectrum of compound (**4u**) in $CDCl_3$.

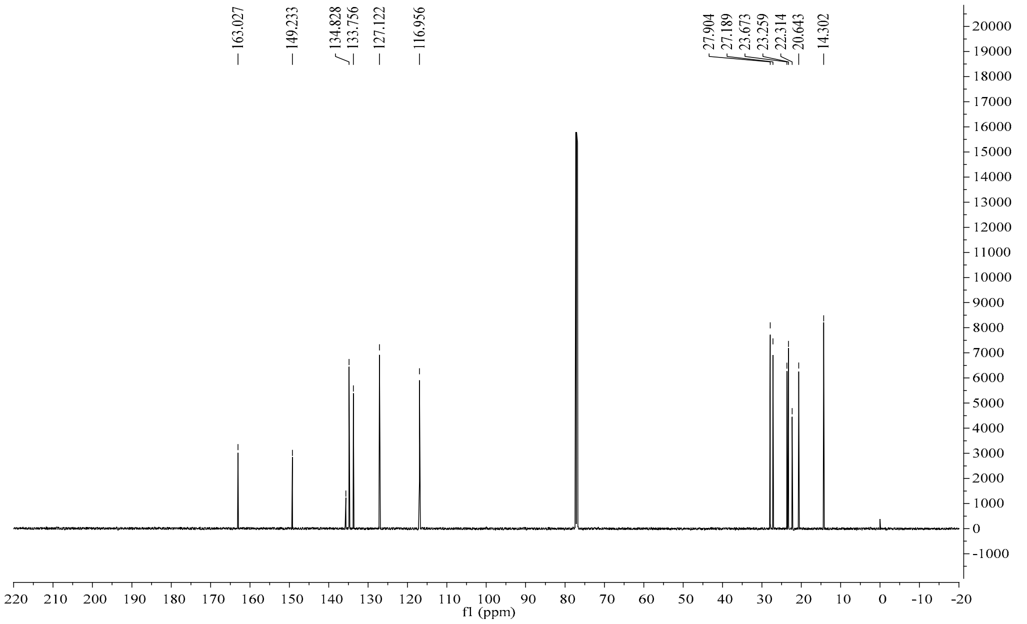

Figure S122. ^{13}C-NMR spectrum of compound (**4u**) in $CDCl_3$.

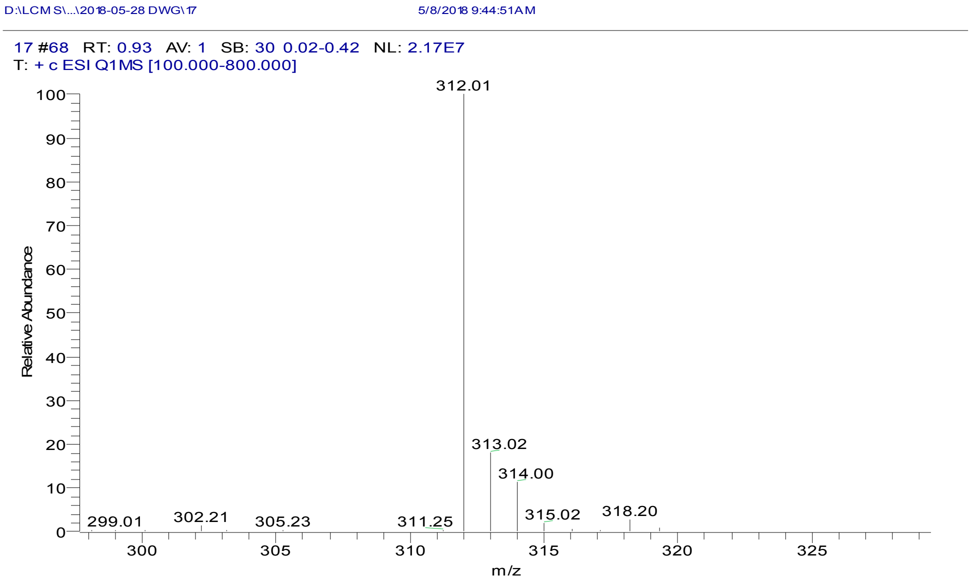

Figure S123. ESI-MS spectrum of compound (**4u).**

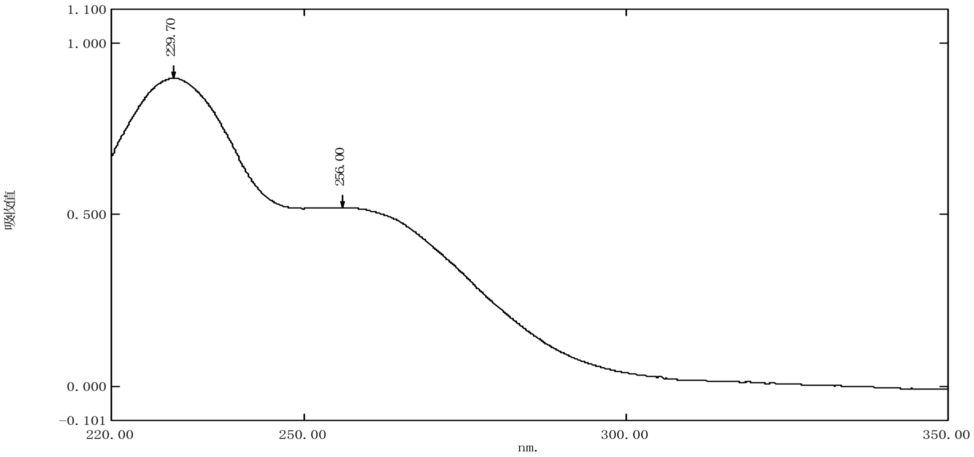

Figure S124. UV-vis spectrum of compound (**4v**).

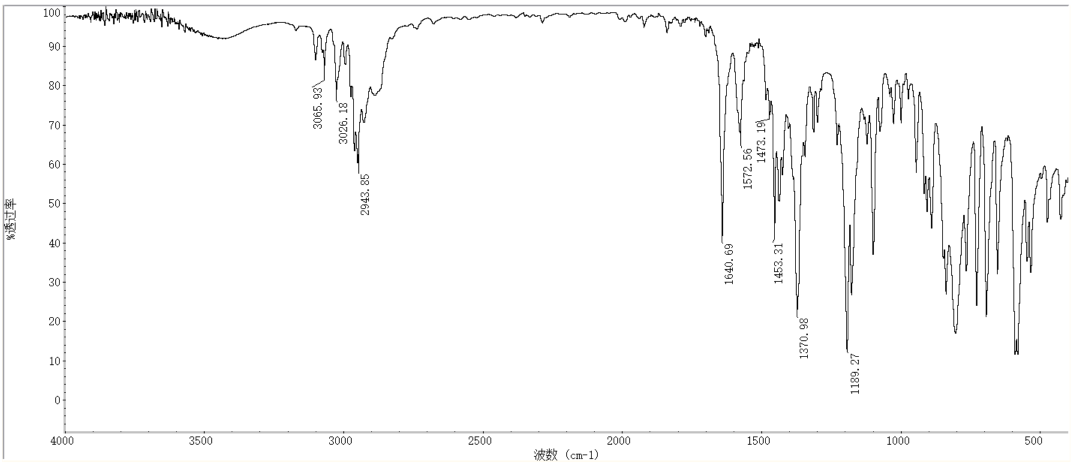

Figure S125. FTIR spectrum of compound (**4v).**

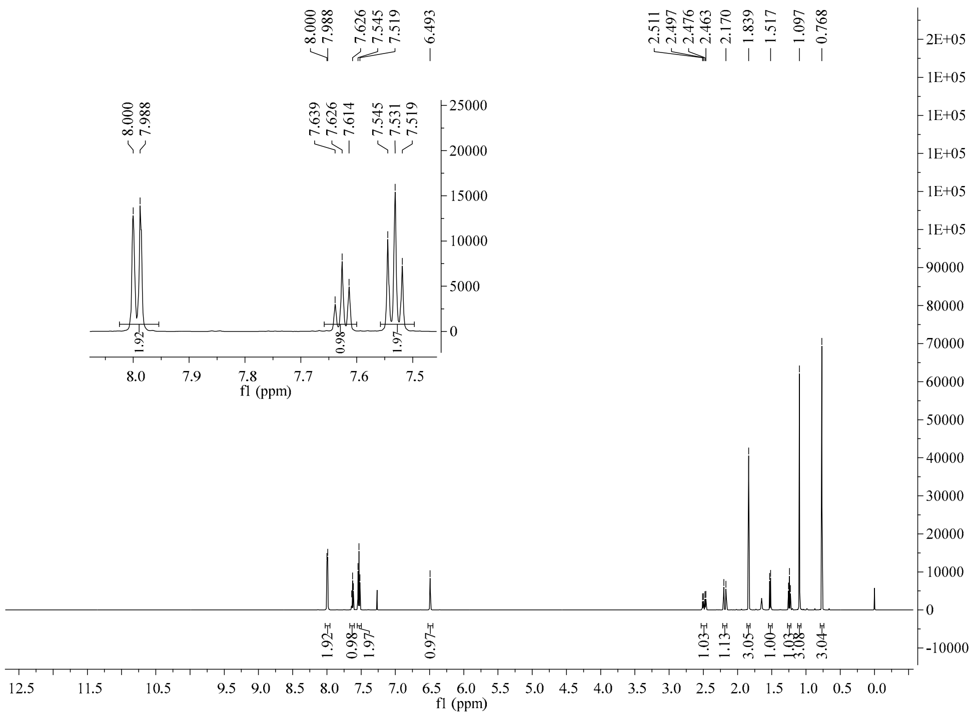

Figure S126. ^{1}H-NMR spectrum of compound (**4v)** in $CDCl_3$.

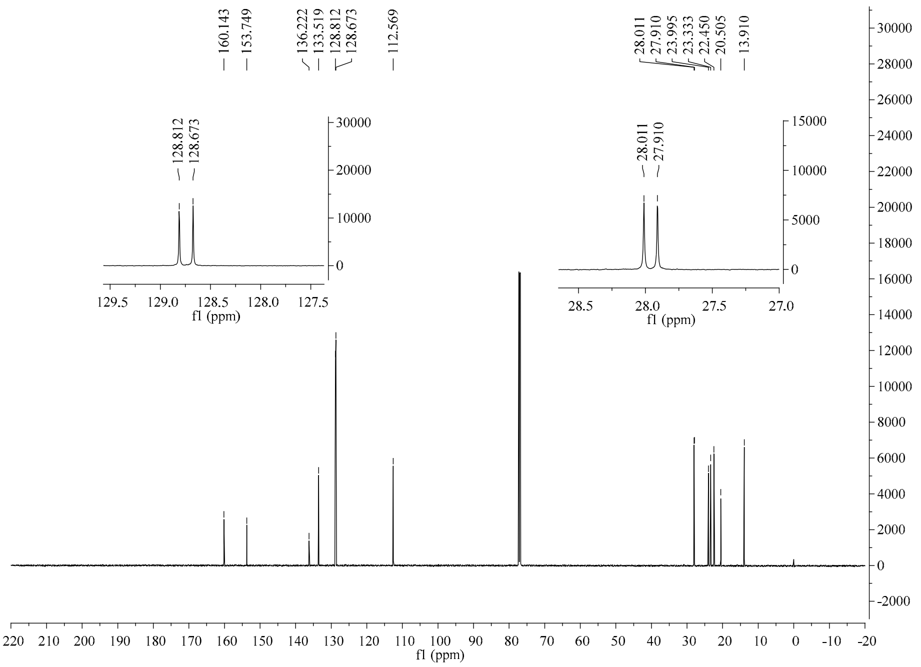

Figure S127. ^{13}C-NMR spectrum of compound (**4v)** in $CDCl_3$.

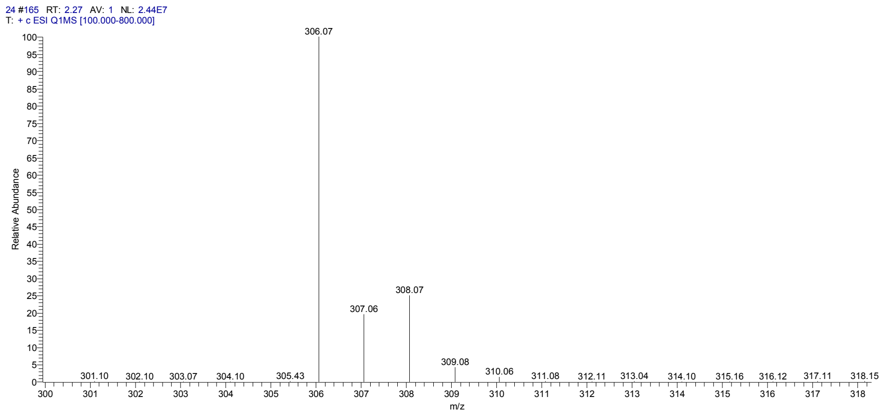

Figure S128. ESI-MS spectrum of compound (**4v**).

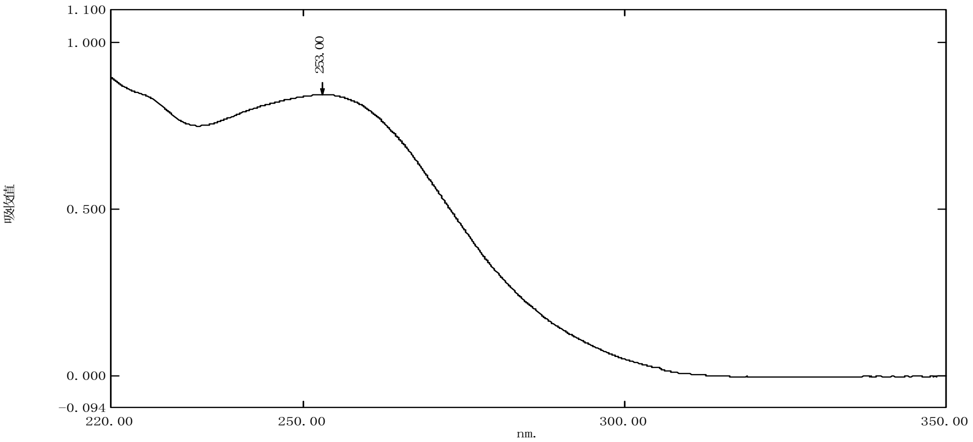

Figure S129. UV-vis spectrum of compound (**4w**).

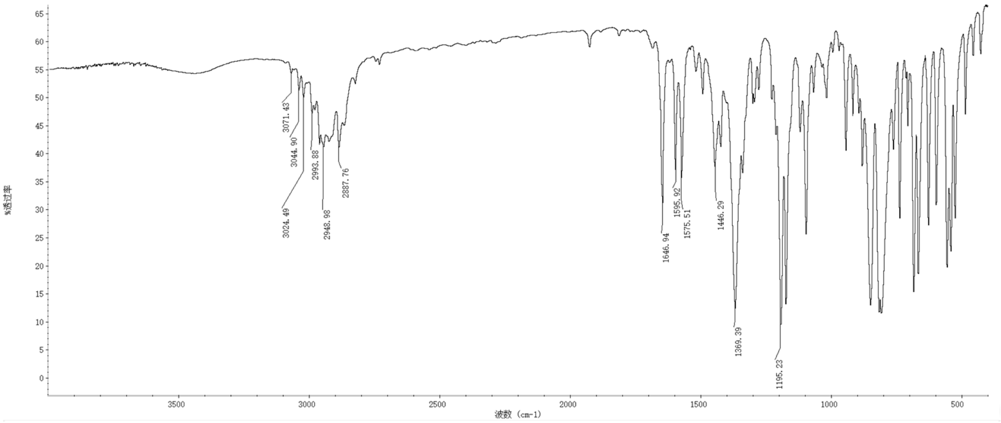

Figure S130. FTIR spectrum of compound (**4w**).

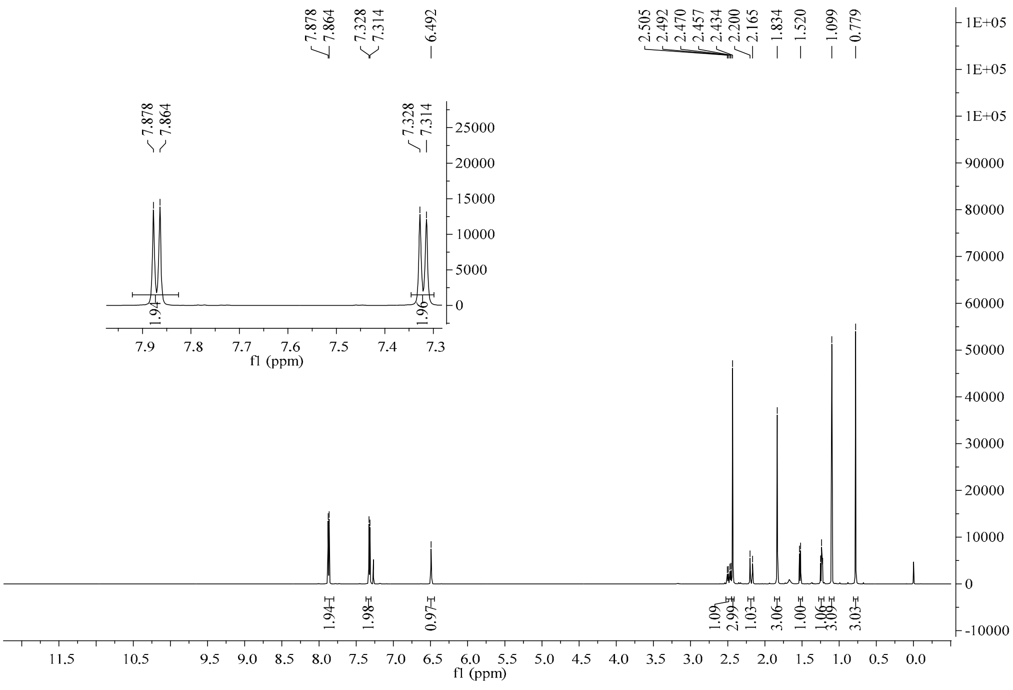

Figure S131. ^{1}H-NMR spectrum of compound (**4w)** in $CDCl_3$.

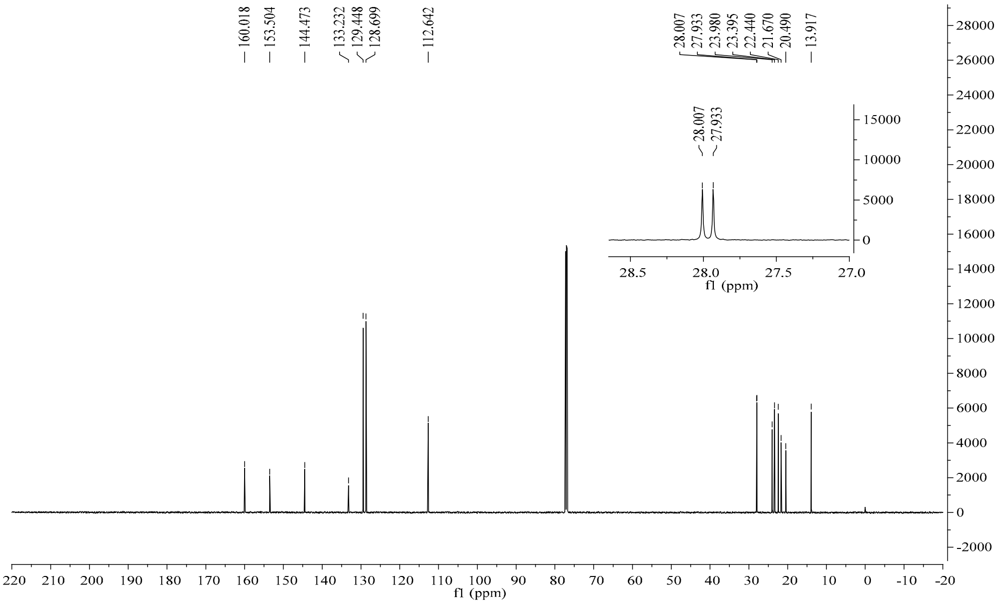

Figure S132. ^{13}C-NMR spectrum of compound (**4w)** in $CDCl_3$.

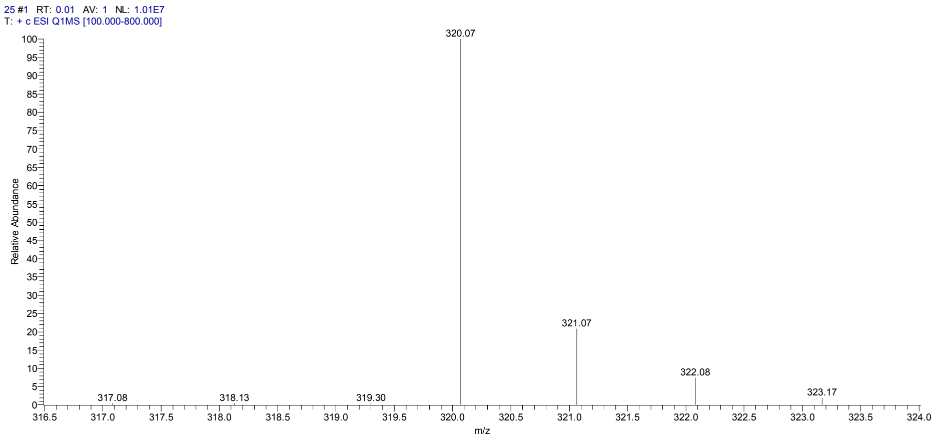

Figure S133. ESI-MS spectrum of compound (**4w).**

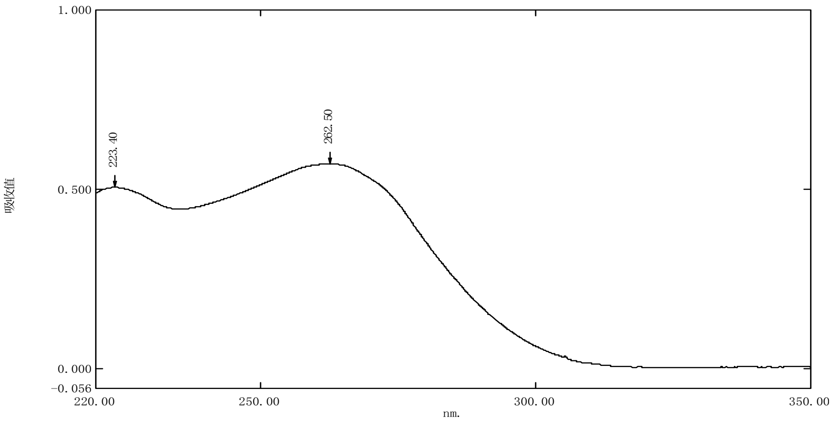

Figure S134. UV-vis spectrum of compound (**4x**).

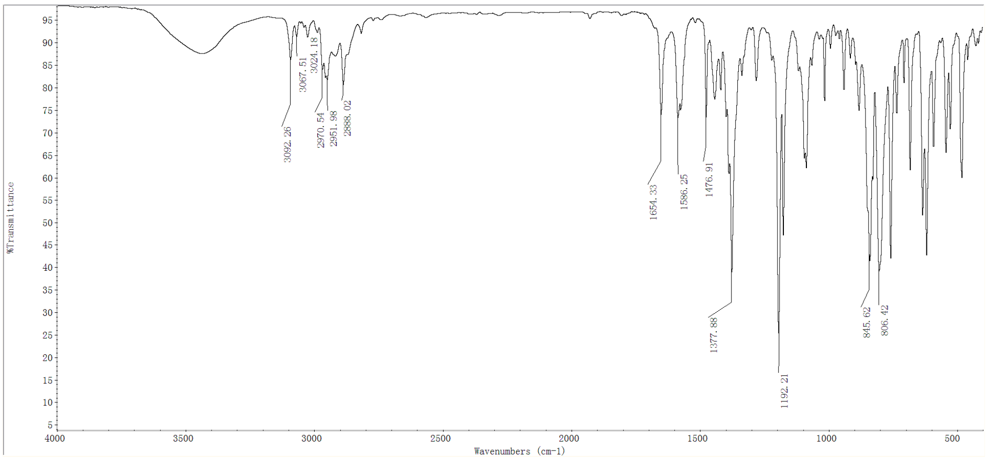

Figure S135. FTIR spectrum of compound (**4x).**

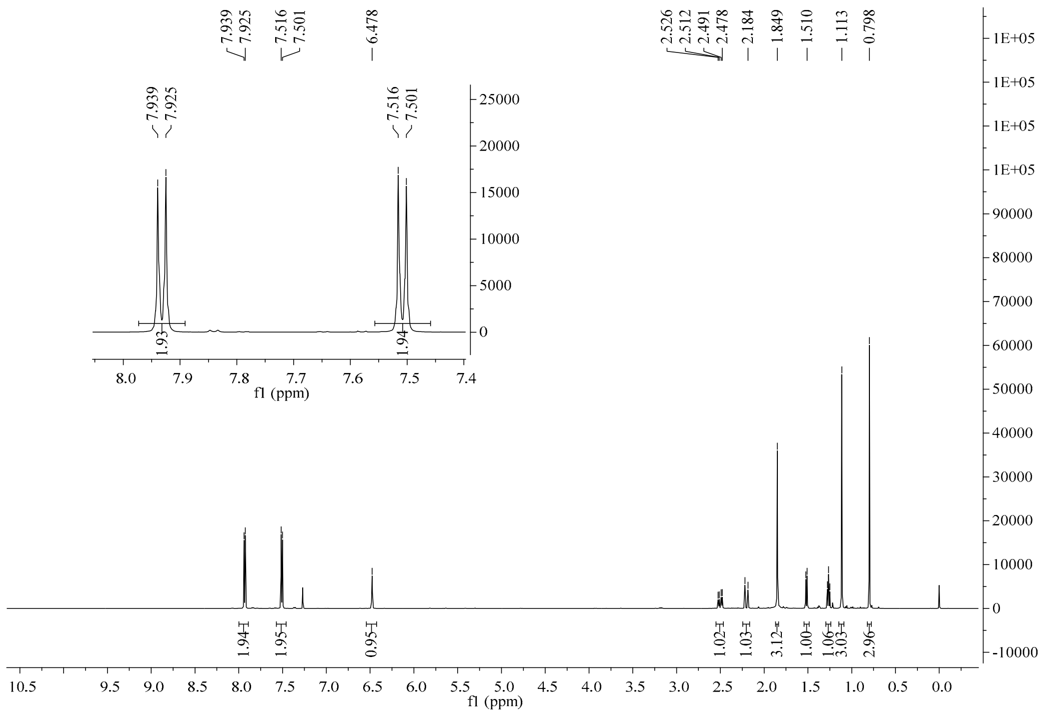

Figure S136. ^{1}H-NMR spectrum of compound (**4x)** in $CDCl_3$.

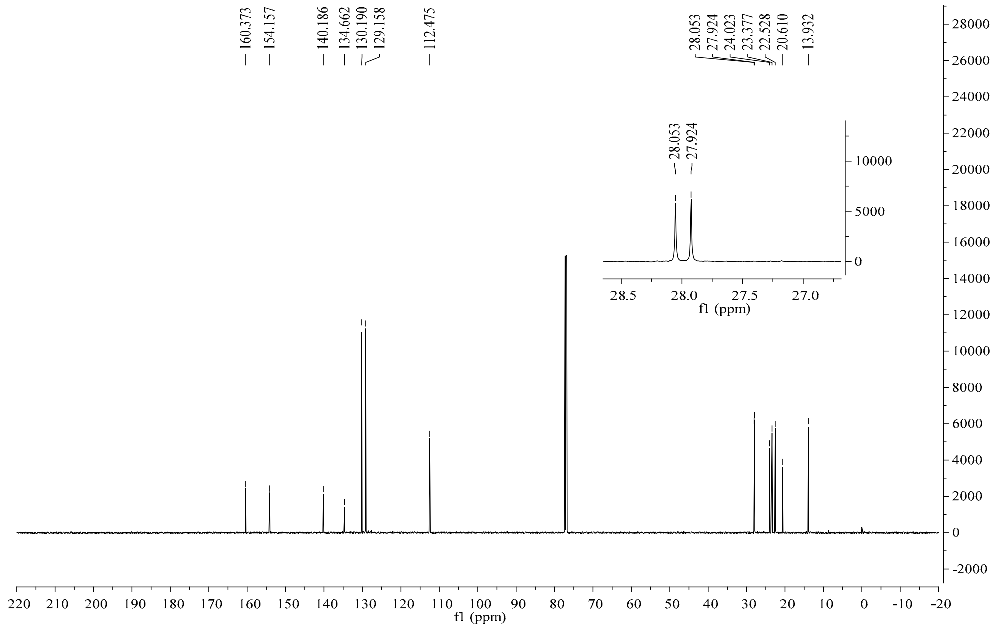

Figure S158. ^{13}C-NMR spectrum of compound (**4x)** in $CDCl_3$.

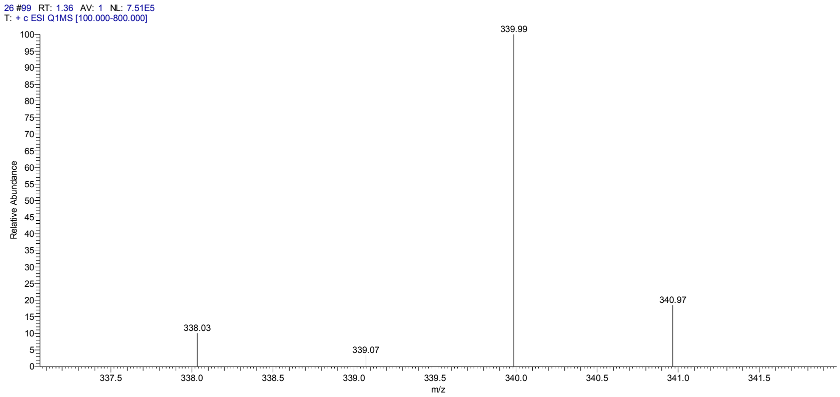

Figure S138. ESI-MS spectrum of compound (**4x).**

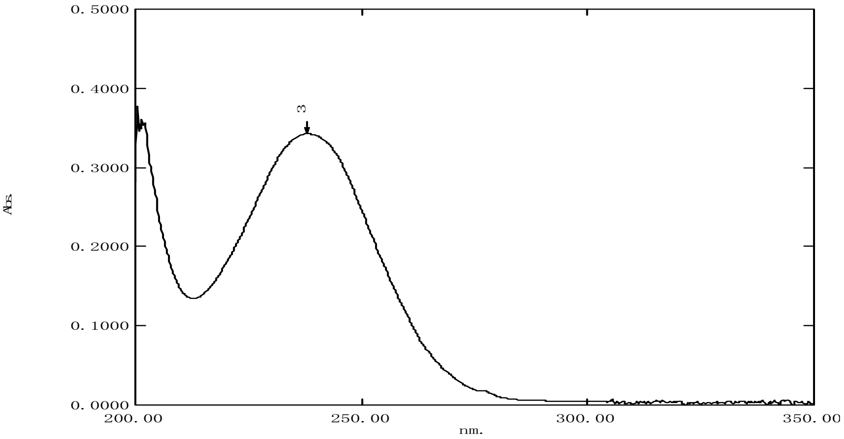

Figure S139. UV-vis spectrum of compound (**4y)**.

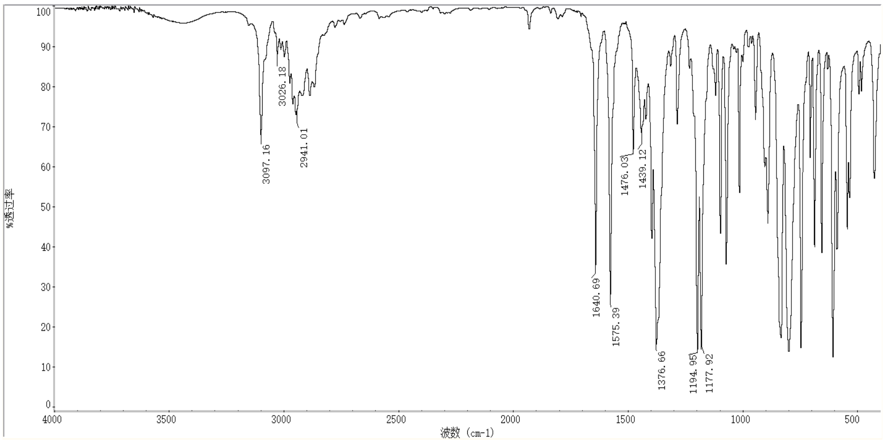

Figure S140. FTIR spectrum of compound (**4y).**

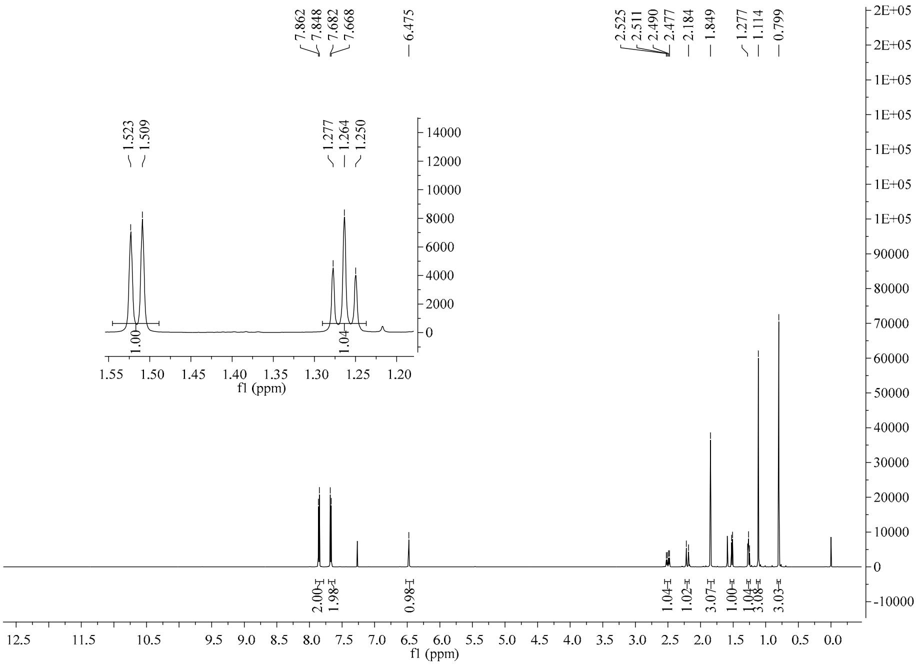

Figure S141. ¹H-NMR spectrum of compound (**4y)** in $CDCl_3$.

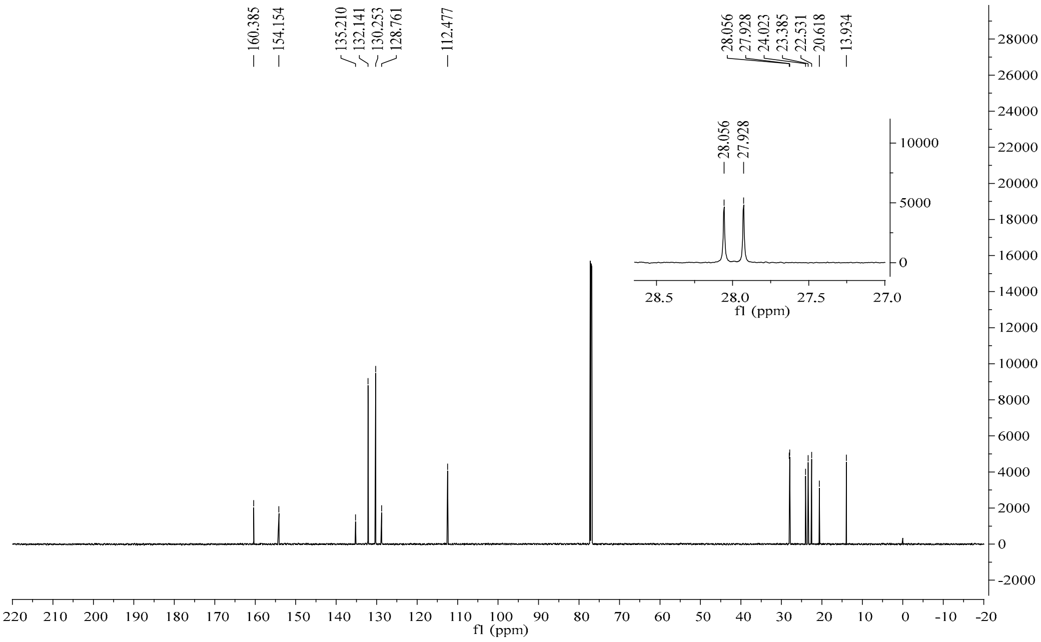

Figure S142. ¹³C-NMR spectrum of compound (**4y**) in $CDCl_3$.

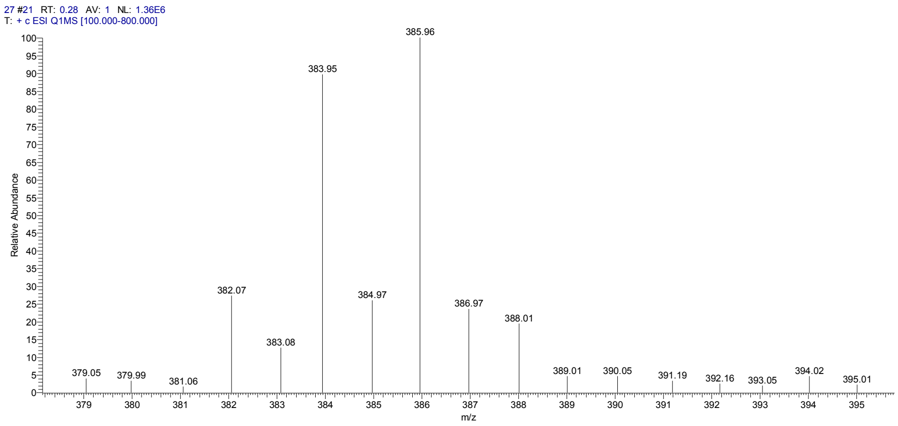

Figure S143. ESI-MS spectrum of compound (**4y).**
